# Stability and C–H Bond Activation Reactions of Palladium(I) and Platinum(I) Metalloradicals: Carbon-to-Metal H-Atom Transfer and an Organometallic Radical Rebound Mechanism

**DOI:** 10.1021/jacs.3c04167

**Published:** 2023-06-15

**Authors:** Tobias Krämer, Matthew R. Gyton, Itxaso Bustos, Matthew J. G. Sinclair, Sze-yin Tan, Christopher J. Wedge, Stuart A. Macgregor, Adrian B. Chaplin

**Affiliations:** † Institute of Chemical Sciences, Heriot-Watt University, Edinburgh EH14 4AS, U.K.; ‡ Department of Chemistry, Maynooth University, Maynooth W23 F2K8, Ireland; § Department of Chemistry, University of Warwick, Gibbet Hill Road, Coventry CV4 7AL, U.K.; ∥ Facultad de Química de San Sebastián, Universidad del País Vasco (UPV/EHU), Apartado 1072, 20080 San Sebastián, Spain; ⊥ Department of Chemical Engineering, Imperial College London, London SW7 2AZ, U.K.; # Department of Physics, University of Warwick, Gibbet Hill Road, Coventry CV4 7AL, U.K.; g Department of Chemical Sciences, University of Huddersfield, Queensgate, Huddersfield HD1 3DH, U.K.

## Abstract

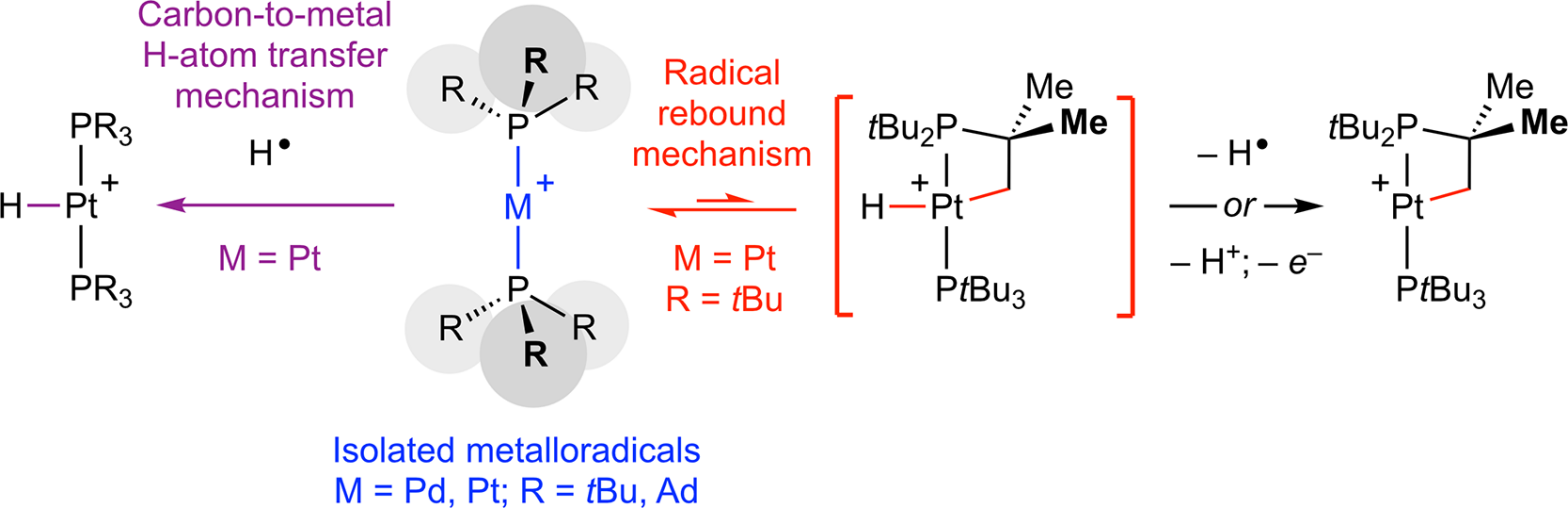

One-electron oxidation of palladium(0) and platinum(0) bis(phosphine) complexes enables isolation of a homologous series of linear d^9^ metalloradicals of the form [M(PR_3_)_2_]^+^ (M = Pd, Pt; R = *t*Bu, Ad), which are stable in 1,2-difluorobenzene (DFB) solution for >1 day at room temperature when partnered with the weakly coordinating [BAr^F^_4_]^−^ (Ar^F^ = 3,5-(CF_3_)_2_C_6_H_3_) counterion. The metalloradicals exhibit reduced stability in THF, decreasing in the order palladium(I) > platinum(I) and PAd_3_ > P*t*Bu_3_, especially in the case of [Pt(P*t*Bu_3_)_2_]^+^, which is converted into a 1:1 mixture of the platinum(II) complexes [Pt(P*t*Bu_2_CMe_2_CH_2_)(P*t*Bu_3_)]^+^ and [Pt(P*t*Bu_3_)_2_H]^+^ upon dissolution at room temperature. Cyclometalation of [Pt(P*t*Bu_3_)_2_]^+^ can also be induced by reaction with the 2,4,6-tri-*tert-*butylphenoxyl radical in DFB, and a common radical rebound mechanism involving carbon-to-metal H-atom transfer and formation of an intermediate platinum(III) hydride complex, [Pt(P*t*Bu_2_CMe_2_CH_2_)H(P*t*Bu_3_)]^+^, has been substantiated by computational analysis. Radical C–H bond oxidative addition is correlated with the resulting M^II^–H bond dissociation energy (M = Pt > Pd), and reactions of the metalloradicals with 9,10-dihydroanthracene in DFB at room temperature provide experimental evidence for the proposed C–H bond activation manifold in the case of platinum, although conversion into platinum(II) hydride derivatives is considerably faster for [Pt(P*t*Bu_3_)_2_]^+^ (*t*_1/2_ = 1.2 h) than [Pt(PAd_3_)_2_]^+^ (*t*_1/2_ ∼ 40 days).

## Introduction

**Scheme 1:**
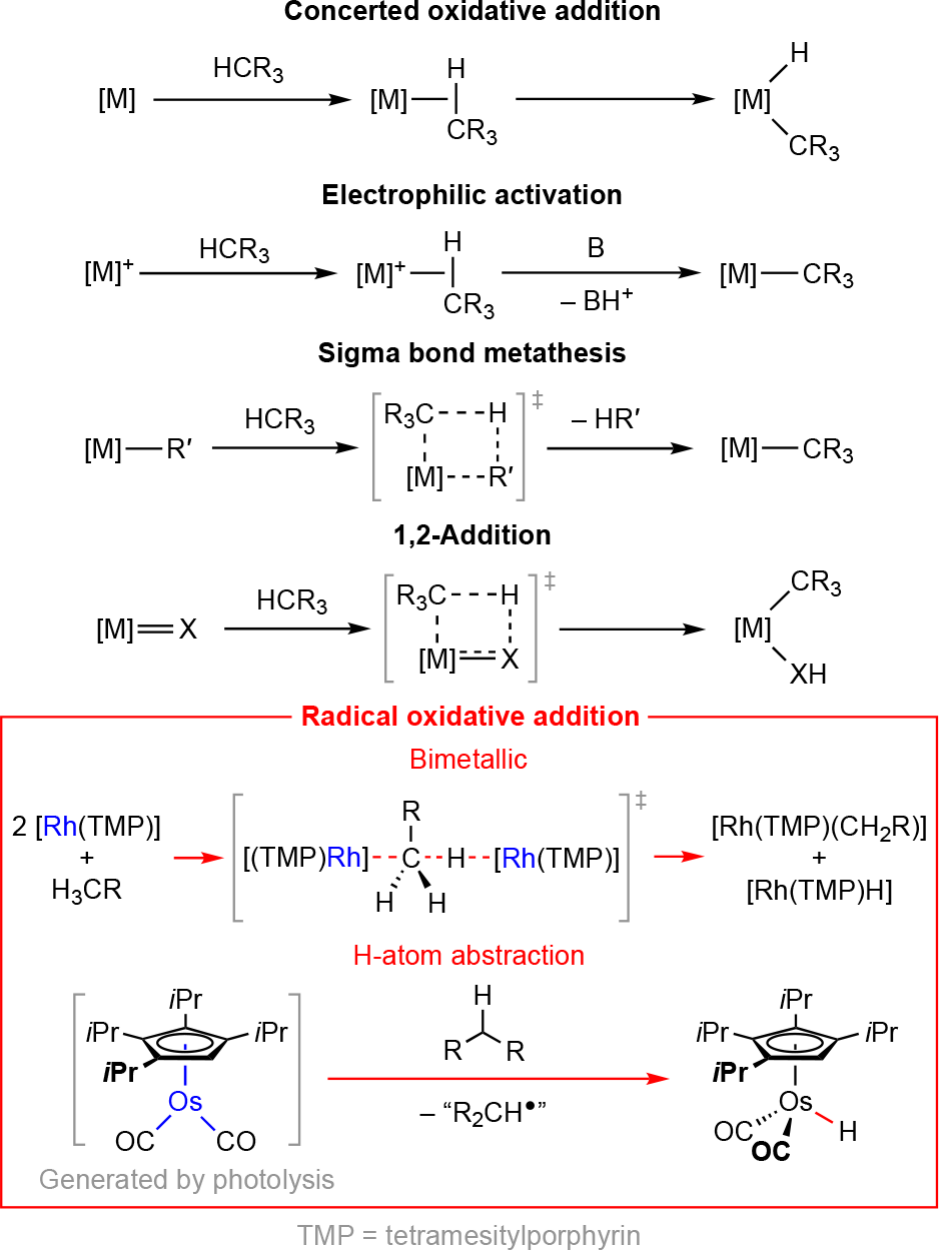
Activation of C(sp^3^)–H Bonds by Late Transition Metal Complexes

With many applications in synthetic organic chemistry,^1^ the development of methods for enacting the cleavage of C(sp^3^)–H bonds is an important facet of contemporary organometallic chemistry.^2^ Building on pioneering work by Bergman and Graham,^2,3^ the activation of these robust and nonpolar σ-bonds by concerted oxidative addition to electron-rich, low-valent platinum group metals is a well-established and exploited mechanism. These reactions proceed via transient three-center-two-electron M–H–C adducts and involve +2 changes in the formal oxidation state of the metal (ΔOS = +2; Scheme 1).^4^ Other distinct manifolds include electrophilic activation (ΔOS = 0), σ-bond metathesis (ΔOS = 0), 1,2-addition across polar metal–ligand multiple bonds (ΔOS = 0), and radical oxidative addition (ΔOS = +1).^2,5^ The last is typically associated with the homolysis of appreciably polar σ-bonds by metalloradicals and is an underdeveloped C(sp^3^)–H bond activation strategy. The most long-standing precedent emerged from Wayland’s work with rhodium(II) porphyrins in the early 1990s, in which a mechanism involving addition of C(sp^3^)–H bonds across two metalloradicals was established experimentally (Scheme 1).^6^ Related bimetallic reactivity has also been invoked in the cyclometalation of a triphenylphosphine-ligated rhodium(II) metalloradical and allylic C(sp^3^)–H bond activation reactions of M^II^(cyclooctadiene) complexes (M = Rh, Ir).^7^ Carbon-to-metal H-atom transfer reactions of a photochemically generated osmium(I) cyclopentadienyl metalloradical with allylic and benzylic substrates is a more recent and notable precedent (Scheme 1).^8^

Low-valent paramagnetic derivatives of palladium and platinum are intriguing candidates to participate in radical oxidative addition reactions; however, the chemistry of complexes of this nature is significantly underdeveloped, especially with reference to the advances being made with d^9^-complexes of nickel.^9−11^ Mononuclear palladium(I) and platinum(I) complexes have been invoked as transient intermediates or generated electrochemically *in situ*,^12,13^ but only a handful have been isolated to date.^14−17^ Instead adoption of the formal +1 oxidation state for palladium and platinum is almost exclusively limited to bimetallic adducts where formation of a metal–metal bond confers a closed-shell electronic configuration.^18^

**Scheme 2:**
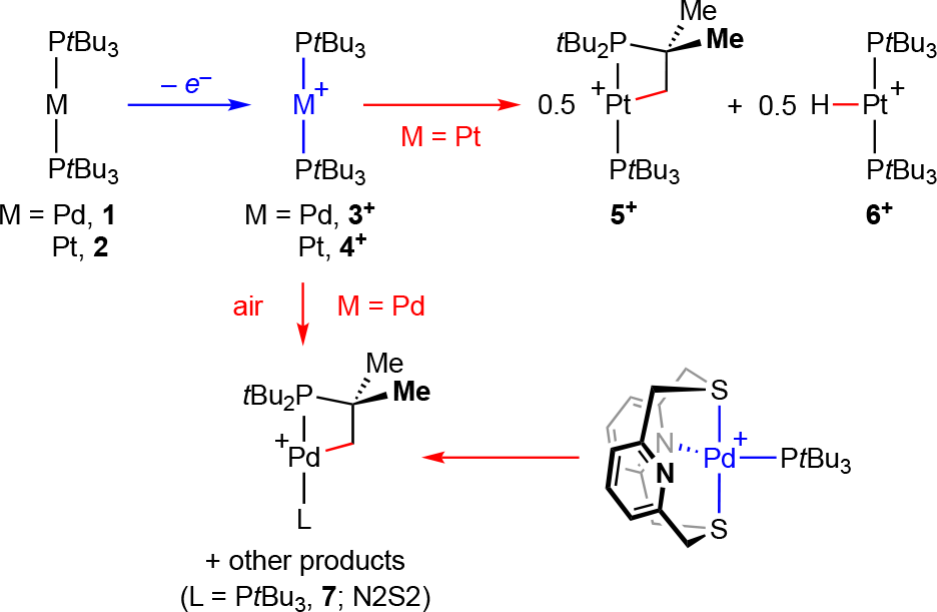
One-Electron Oxidation of [M(P*t*Bu_3_)_2_] and Subsequent Reactivity

Building upon work by Stalke, Frenking, Roesky, and co-workers, who examined the electrochemical oxidation of two-coordinate cyclic alkyl(amino) carbene complexes of palladium(0) and platinum(0),^12^ some of us demonstrated the facile and reversible one-electron oxidation of [M(P*t*Bu_3_)_2_] (M = Pd, **1**; Pt, **2**) using cyclic voltammetry in 2016.^14^ Subsequent reaction of **1** with Fc[PF_6_] (Fc = FeCp_2_) in 1,2-difluorobenzene (DFB) enabled isolation of [Pd(P*t*Bu_3_)_2_][PF_6_] (**3**[PF_6_]) in high yield, which proved to be stable in solution for prolonged periods of time under an inert atmosphere at room temperature (Scheme 2). Under the same conditions, however, the corresponding platinum(I) metalloradical [Pt(P*t*Bu_3_)_2_][PF_6_] (**4**[PF_6_]) could not be isolated, and instead a 1:1 mixture of platinum(II) metallocycle [Pt(P*t*Bu_2_CMe_2_CH_2_)(P*t*Bu_3_)][PF_6_] (**5**[PF_6_]) and platinum(II) hydride [Pt(P*t*Bu_3_)_2_H][PF_6_] (**6**[PF_6_]) was observed. This outcome corresponds to *net* radical oxidative addition of a C(sp^3^)–H bond across two transient platinum(I) metalloradicals, although the precise mechanism was not resolved at the time. Zhou, Meyer, Hughes, Ozerov, and co-workers later reported the isolation of both metalloradicals using fluorobenzene as a solvent and [Ph_3_C][HCB_11_Cl_11_] as the one-electron oxidant, viz. **3**[HCB_11_Cl_11_] and **4**[HCB_11_Cl_11_].^15^ Pairing the platinum(I) metalloradical with the weakly coordinating [HCB_11_Cl_11_]^−^ counterion curbs onward reactivity but did not prevent complete conversion of **4**^**+**^ into a 1:1 mixture of **5**^**+**^ and **6**^**+**^, which occurred within 48 h at room temperature in fluorobenzene. Underscoring the propensity for cyclometalation, however, dissolution of **4**[HCB_11_Cl_11_] in acetonitrile at room temperature resulted in instantaneous conversion into a 1:1 mixture of [Pt(P*t*Bu_2_CMe_2_CH_2_)(P*t*Bu_3_)(NCMe)][HCB_11_Cl_11_] and [Pt(P*t*Bu_3_)_2_H(NCMe)][HCB_11_Cl_11_].

Under certain conditions, we have since observed onward reactivity of **3**[PF_6_] that results in formation of [Pd(P*t*Bu_2_CMe_2_CH_2_)(P*t*Bu_3_)][PF_6_] (**7**), most notably upon exposure of the palladium(I) metalloradical to air.^19^ Similar reactivity has recently been noted by Mirica and co-workers while exploring the use of dithiapyridinophane-ligated palladium(I) complexes in Kumada cross-coupling reactions (Scheme 2).^16^ Both reactions invoke radical oxidative addition of a C(sp^3^)–H bond but, compared to cyclometalation of **4**^**+**^, are less well defined.

We herein present further findings from our work exploring the chemistry of two coordinate palladium(I) and platinum(I) metalloradicals,^20^ focused on uncovering the mechanism of C(sp^3^)–H bond activation by late transition metal metalloradicals. Examples with enhanced solution stability are described along with a detailed computational examination of different radical-based mechanisms of C(sp^3^)–H bond cyclometalation and other onward reactivity of **4**^**+**^.

## Results and Discussion

### Isolation and Stability of Tri-*tert*-butylphosphine-Ligated Metalloradicals

Preceding work has highlighted the instability of **4**^**+**^, for which isolation requires careful consideration of the counterion and solvent used. We chose to focus our attention on the partially fluorinated tetraarylborate counterion [BAr^F^_4_]^−^ (Ar^F^ = 3,5-(CF_3_)_2_C_6_H_3_). This weakly coordinating anion finds widespread utility for the stabilization of reactive and low-coordinate metal cations,^21^ while its use is practically convenient, as the sodium salt is commercially available. Straightforward synthetic procedures that have been optimized for the isolation of solvent-free anhydrous M[BAr^F^_4_] (M = Li, Na, K) have also been reported recently.^22^

**Figure 1:**
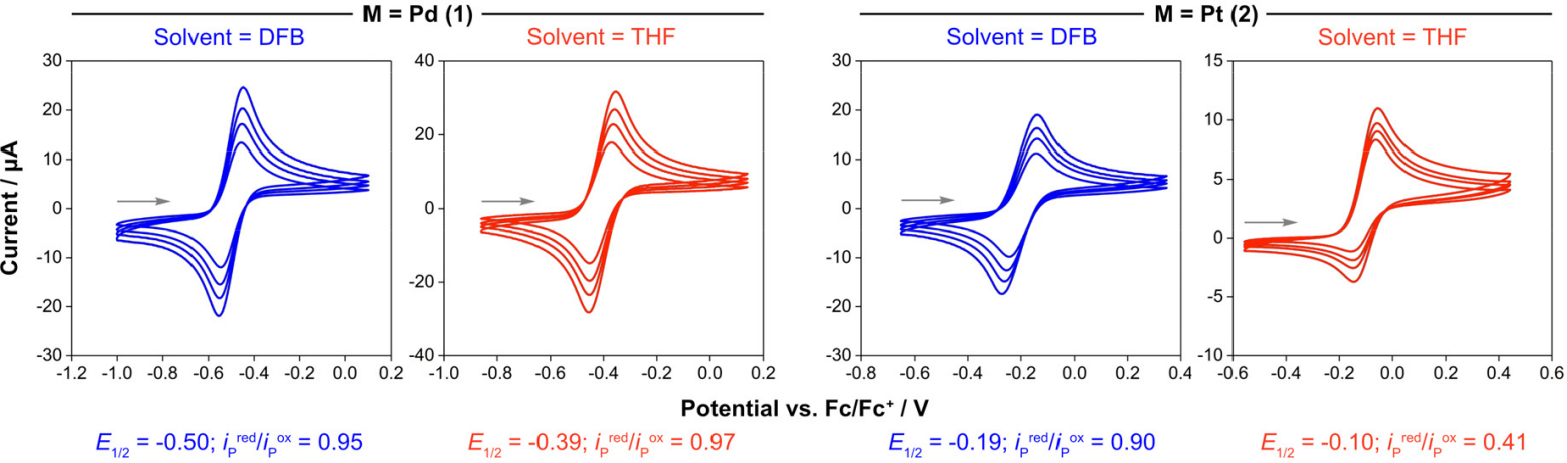
Cyclic voltammograms for the oxidation of [M(P*t*Bu_3_)_2_] in DFB and THF at room temperature (2 mM complex; 0.2 M [*n*Bu_4_N][BAr^F^_4_] electrolyte; glassy carbon working electrode, coiled Pt wire counter electrode, and Ag wire quasi-reference electrode; scan rates = 30, 50, 70, and 100 mV·s^–1^).

To probe the role of solvent on the stability of **3**^**+**^ and **4**^**+**^, we began by re-evaluating the oxidation of **1** and **2** by cyclic voltammetry using [*n*Bu_4_N][BAr^F^_4_] as the electrolyte and DFB and tetrahydrofuran (THF) as solvents (Figure 1). Quasi-reversible one-electron oxidation was observed in all cases and, noting that considerable variance is to be expected when changing the electrolyte and solvent,^23^ the resulting redox potentials are in line with what we have previously determined under different conditions (*E*_1/2_ = −0.44 V, **1**; −0.10 V **2**; DFB/[*n*Bu_4_N][PF_6_], relative to Fc/Fc^+^). Using deviation of the peak current ratio (*i*_P_^red^/*i*_P_^ox^) from unity as a gauge, it is apparent that the platinum metalloradical (*i*_P_^red^/*i*_P_^ox^ ≤ 0.90) is considerably less stable than the palladium congener (*i*_P_^red^/*i*_P_^ox^ ≥ 0.95). Moreover, use of THF as the solvent considerably accelerates the onward reactivity of **4**^**+**^ (*i*_P_^red^/*i*_P_^ox^ = 0.41 cf. 0.90): to the point that the stability of this metalloradical is limited to seconds at room temperature (*vide infra*).

**Scheme 3:**
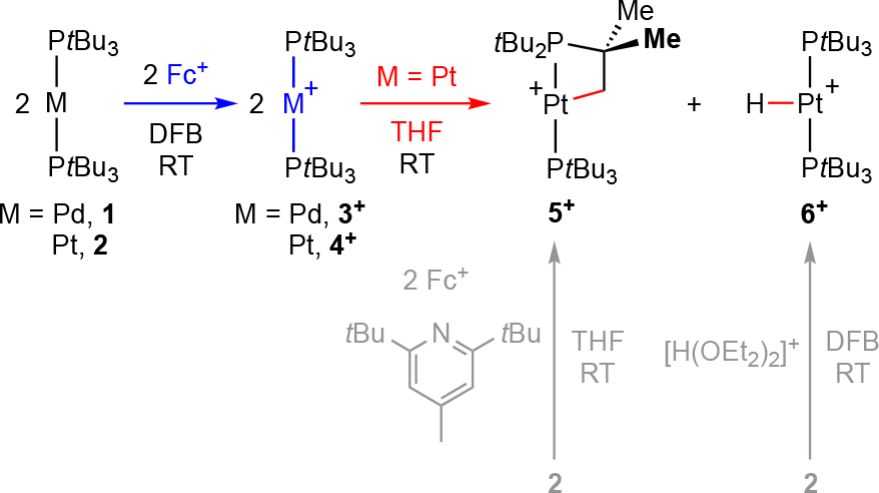
Synthesis and Onward Reactivity of [M(P*t*Bu_3_)_2_][BAr^F^_4_] [BAr^F^_4_]^−^ counterions omitted for clarity.

Subsequent to the electrochemical study, **3**[BAr^F^_4_] and **4**[BAr^F^_4_] were prepared by chemical oxidation of **1** and **2** using Fc[BAr^F^_4_] in rigorously dried DFB at room temperature (Scheme 3).^24^ Although the palladium metalloradical was readily isolated in high purity and 87% yield, obtaining analytically pure samples of the heavier congener using this method was less reproducible and samples were often contaminated with small amounts of the platinum(II) hydride **6**[BAr^F^_4_].^25^ This impurity appears to cocrystallize with **4**[BAr^F^_4_], as subsequent recrystallization did not result in improvements in purity. Consistent with this assertion, independently isolated **6**[BAr^F^_4_] obtained from the reaction between **2** and [H(OEt_2_)_2_][BAr^F^_4_] in DFB (Scheme 3) is isomorphous to **4**[BAr^F^_4_] (see Supporting Information).^26^ In our experience, the most reliable way of obtaining high-purity samples of **4**[BAr^F^_4_] was by performing the reaction in the presence of 0.05 equiv of 2,6-di-*tert*-butyl-4-methylpyridine (62% yield, >99% purity).

Complexes **3**[BAr^F^_4_] and **4**[BAr^F^_4_] were extensively characterized in DFB and unsurprisingly show directly comparable spectroscopic and electrochemical signatures to those previously reported for **3**^**+**^ and **4**^**+**^.^14,15^ Most notably, deep blue **3**[BAr^F^_4_] is characterized by a singlet EPR resonance with axial *g*-tensor, *g*_⊥_*=* 2.343 and *g*_∥_ = 1.978, superimposed with a lower intensity sextet arising from isotropic hyperfine coupling to ^105^Pd (*I* = 5/2, 22% abundance, *a* = 25.2 mT; no superhyperfine coupling to ^31^P was evident; DFB glass @ 200 K), but a ^31^P NMR resonance could not be observed between +500 and −500 ppm. In contrast, for green **4**[BAr^F^_4_] a paramagnetically shifted ^31^P NMR resonance could be located at δ −213.9, while an EPR signal could not be detected down to 100 K (DFB glass). The UV–vis spectrum of **4**^**+**^ has not previously been reported and is most remarkable for a sharp band at 306 nm (ε = 700 M^–1^·cm^–1^) ascribed to a metal-centered transition.^27^ Further details, including analysis by time-dependent density functional theory (TD-DFT) calculations, are provided in the Supporting Information.

**Figure 2:**
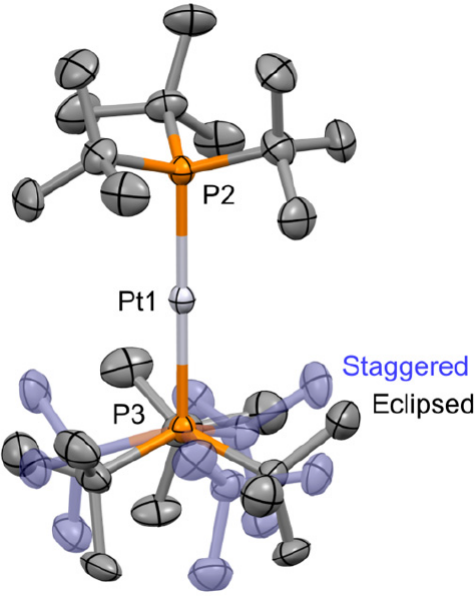
Solid-state structure of **4**[BAr^F^_4_]. Anisotropic displacement ellipsoids drawn at 30% probability; hydrogen atoms and anion omitted for clarity. Symmetry equivalent phosphine substituents are generated using the operations: ^3^/_2_–*z*, 1–*x*, ^1^/_2_+*y* and 1–*y*, −^1^/_2_+*z*, ^3^/_2_–*x*. Selected data: isomorphous **3**[BAr^F^_4_], Pd1–P2, 2.349(3) Å; Pd1–P3, 2.353(3) Å; P2–Pd1–P3, 180°; 57.5(10)% eclipsed; **4**[BAr^F^_4_], Pt1–P2, 2.333(4) Å; Pt1–P3, 2.338(4) Å; P2–Pt1–P3, 180°; 52.9(11)% eclipsed.

The solid-state structures of the new metalloradicals have also been determined and are isomorphic, crystallizing in the cubic *P*2_1_3 space group with the P–M–P vector lying along a 3-fold rotation axis (M = Pt, Figure 2). A disordered mixture of staggered and eclipsed cations in approximately equal ratios is evident in both structures, contrasting the solid-state structures of **3**[PF_6_], **3**[HCB_11_Cl_11_], and **4**[HCB_11_Cl_11_], which feature metal centers that lie on an inversion center and crystallographically imposed staggered conformations. There are, however, no statistically significant differences in the associated M–P bond lengths for **3**^**+**^ (2.3470(6) Å, [PF_6_]^−^; 2.3466(5) Å, [HCB_11_Cl_11_]^−^; 2.349(3)/2.353(3) Å, [BAr^F^_4_]^−^; avg 2.349(4) Å) and **4**^**+**^ (2.3362(6), [HCB_11_Cl_11_]^−^; 2.333(4)/2.338(4) Å, [BAr^F^_4_]^−^; avg 2.336(6) Å). As previously noted, these bonds are appreciably elongated relative to the corresponding zerovalent precursors **1** (2.285(3) Å) and **2** (2.249(3) Å).^28,29^ The conclusion of the computational analysis conducted by Ozerov et al. is that the unpaired electrons in **3**^**+**^ and **4**^**+**^ belong to nonbonding orbitals and bond elongation results from a perturbation of the attractive electrostatic and repulsive Pauli forces.^15^ In relation to the bulk purity, the Pt–P distances observed for **4**[BAr^F^_4_] are longer on average than those found in **6**[BAr^F^_4_] (2.314(3)/2.320(3) Å).

**Scheme 4:**
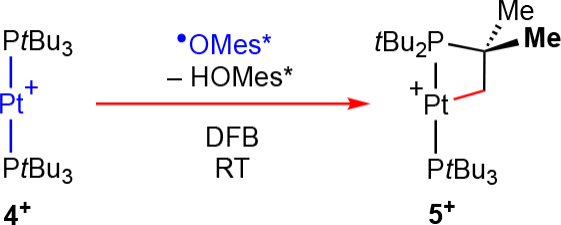
Conversion of **4**[BAr^F^_4_] into **5**[BAr^F^_4_] by Reaction with ^•^OMes* (2,4,6-Tri-*tert-*butylphenoxy) [BAr^F^_4_]^−^ counterions omitted for clarity.

Under an inert atmosphere **3**[BAr^F^_4_] and **4**[BAr^F^_4_] are stable in DFB, with no appreciable onward reactivity observed upon standing at room temperature for 24 h. Formation of green [Pd(P*t*Bu_3_)_2_(NCMe)][HCB_11_Cl_11_] has previously been established for the palladium metalloradical in acetonitrile,^15^ but there are no significant changes to the UV–vis spectrum of **3**[BAr^F^_4_] upon dissolution in THF at room temperature that would suggest formation of a similar adduct in this solvent. Consistent with the electrochemical study, the metalloradicals show contrasting stability in THF at room temperature. Partial decomposition of **3**[BAr^F^_4_] (ca. 20%) was observed upon standing in solution for 24 h, while rapid conversion into a 1:1 mixture of platinum(II) metallocycle **5**[BAr^F^_4_] (δ_31P_ 59.1, ^1^*J*_PtP_ = 2899 Hz; 25.3, ^1^*J*_PtP_ = 1915 Hz; ^2^*J*_PP_ = 317 Hz) and platinum(II) hydride **6**[BAr^F^_4_] (δ_1H_ −35.28, ^1^*J*_PtH_ = 2540 Hz; δ_31P_ 86.5, ^1^*J*_PtP_ = 2631 Hz) was observed upon dissolution of **4**[BAr^F^_4_] (Scheme 3). No deuterium incorporation into the products occurred when the latter was repeated in *d*_8_-THF, and the identity of **5**[BAr^F^_4_] and **6**[BAr^F^_4_] was confirmed by comparison to literature data and independent synthesis from **2** (Scheme 3).^14,26^ While **4**[BAr^F^_4_] persists in DFB, reaction with the 2,4,6-tri-*tert-*butylphenoxyl radical (^•^OMes*) resulted in smooth conversion into **5**[BAr^F^_4_] with high selectivity (ca. 90%) at room temperature within 8 days (Scheme 4). Consumption of the metalloradical occurred with apparent first-order kinetics (*t*_1/2_ = 49 h) under these conditions, suggesting that ^•^OMes* is acting as an H-atom trap.

### Computational Evaluation of Radical Oxidative Addition Pathways

**Figure 3:**
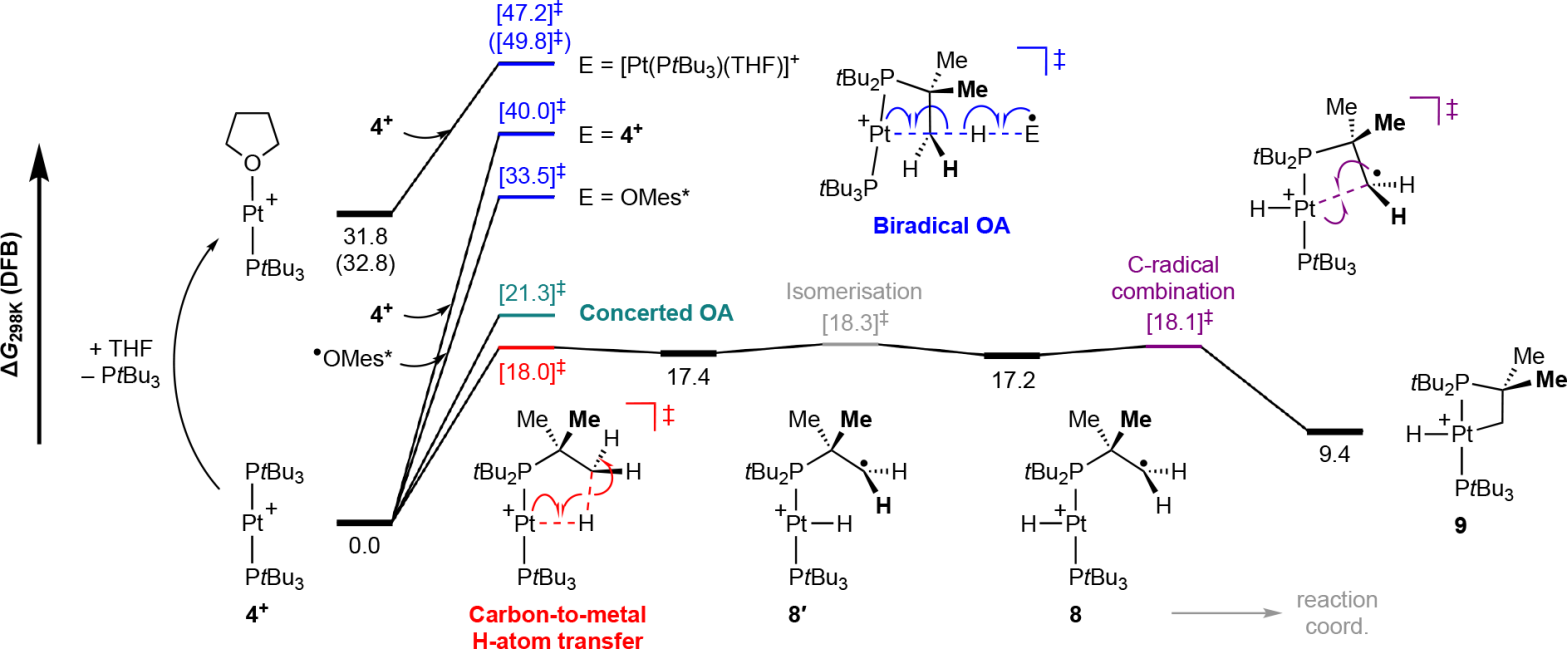
Computed reaction profiles (B2PLYP-D3(BJ)/def2-TZVPP+def-ECP(Pt)//PBEh-3c) for the C–H bond cyclometalation of **4**^**+**^. Energies corrected for DFB solvent in kcal·mol^–1^ (selected THF values given in parentheses).

To further interrogate the mechanism associated with C(sp^3^)–H bond cyclometalation of **4**^**+**^, we turned to unrestricted DFT calculations to analyze the viability of possible reaction pathways (Figure 3). Following benchmarking, geometries were optimized in the gas phase using the PBEh-3c composite method,^30^ and single-point energies were calculated at the B2PLYP-D3(BJ)/def-TZVPP^31,32^ level of theory with corrections included for London dispersion and solvation effects.^33,34^

The prospect for Wayland-like bimetallic radical C–H bond oxidative activation was first examined using an antiferromagnetically spin-polarized four-centered transition state of the form Pt(↓)···C(↑)···H(↓)···Pt(↑) derived from two equivalents of **4**^**+**^ and producing **5**^**+**^ and **6**^**+**^ in one step.^35^ The calculations indicate that this intermolecular process is associated with a prohibitively high activation barrier of Δ*G*^⧧^_298K_ = 40.0 kcal·mol^–1^. The bulky P*t*Bu_3_ ancillary ligands appear to encumber the approach of the two metal centers, and the hydridic character of the transition state suggests homolysis of the C–H bond occurs with a significant degree of asymmetry (Pt···C = 3.30 vs 2.04 Å in **5**^**+**^; H···Pt = 1.62 vs 1.50 Å in **6**^**+**^; Pt···Pt = 6.19 Å). With the former in mind, and in an attempt to reconcile the large solvent dependence of the reaction, the possibility for ligand exchange with THF to generate the less bulky metalloradical [Pt(P*t*Bu_3_)(THF)]^+^ as the H-atom acceptor was also considered. In this scenario, cyclometalation of **4**^**+**^ occurs with more symmetric homolysis of the C–H bond (Pt···C = 2.61 Å; H···Pt = 1.63 Å) and a reduced activation barrier of Δ*G*^⧧^_298K_ = 15.4 kcal·mol^–1^, but initial substitution of P*t*Bu_3_ to form [Pt(P*t*Bu_3_)(THF)]^+^ renders the overall process energetically inaccessible (Δ*G*^⧧^_298K_ = 47.2 kcal·mol^–1^).^36^ Likewise, conversion of **4**^**+**^ into **5**^**+**^ by reaction with ^•^OMes* via a four-centered transition state of the form Pt(↓)···C(↑)···H(↓)···O(↑) can be ruled out on the basis of a large activation barrier of Δ*G*^⧧^_298K_ = 33.5 kcal·mol^–1^.

More promisingly, intramolecular cyclometalation of **4**^**+**^, resulting in the platinum(III) metalloradical [Pt(P*t*Bu_2_CMe_2_CH_2_)H(P*t*Bu_3_)]^+^ (**9**), is calculated to be a moderately endergonic process (Δ*G*_298K_ = +9.4 kcal·mol^–1^). A pathway commencing with concerted C–H bond oxidative addition (Δ*G*^⧧^_298K_ = 21.3 kcal·mol^–1^) to the metalloradical can be located between **4**^**+**^ and **9**, but our calculations suggest stepwise insertion into the C–H bond is considerably more favorable with an activation barrier of only Δ*G*^⧧^_298K_ = 18.3 kcal·mol^–1^ (Figure 3). The latter commences with carbon-to-metal H-atom transfer (Δ*G*^⧧^_298K_ = 18.0 kcal·mol^–1^) and culminates in the formation of **9** following combination of the resulting pendent C-centered radical with the platinum(II) center in [Pt(P*t*Bu_2_CMe_2_CH_2_^•^)H(P*t*Bu_3_)]^+^ (**8**). Radical rebound sequences of this nature were initially considered, but subsequently ruled out, as part of early mechanistic work on C(sp^3^)–H bond activation reactions of diamagnetic iridium(I) cyclopentadienyl complexes by Janowicz and Bergman.^37^ There are, however, strong parallels with the established catalytic action of metal-oxo-based enzymes^38^ and experimental precedent for the formation of mononuclear Pt(III) complexes.^39^ Consistent with the higher relative solution stability of **3**^**+**^ observed, the activation barrier calculated for intramolecular carbon-to-metal H-atom transfer is considerably larger (Δ*G*^⧧^_298K_ = 33.5 kcal·mol^–1^) than for **4**^**+**^. This difference is attributed to the inherently weaker nature of the Pd^II^–H bond (calcd *D*_e_ = 64.4 kcal·mol^–1^) compared to the Pt^II^–H bond (calcd *D*_e_ = 78.5 kcal·mol^–1^).

**Figure 4:**
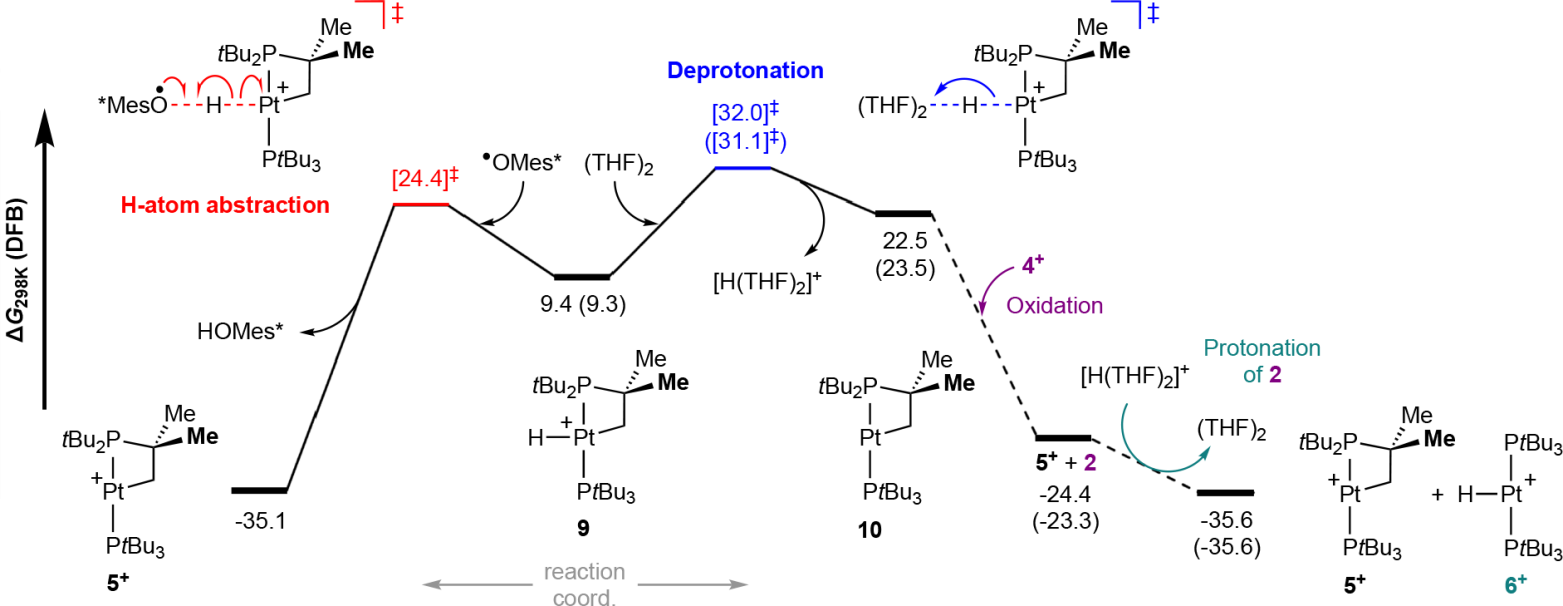
Computed reaction profiles (B2PLYP-D3(BJ)/def2-TZVPP+def-ECP(Pt)//PBEh-3c) for H-atom transfer reactions of **9** resulting in formation of **5**^**+**^. Energies corrected for DFB solvent in kcal·mol^–1^ (selected THF values given parentheses).

Experimentally observed conversion of **4**^**+**^ into **5**^**+**^ by reaction with ^•^OMes* at room temperature can be reconciled by direct H-atom abstraction from **9** (Figure 4). Noting the challenges associated with accurately predicting the entropic contributions of bimolecular transition states using static DFT calculations,^40^ the calculated activation barrier (Δ*G*^⧧^_298K_ = 24.4, Δ*H*^⧧^ = 5.6 kcal·mol^–1^ vs **4**^**+**^) is consistent with the suggested role of ^•^OMes* as an H-atom trap.^41^ In the case of the solvent-induced formation of a 1:1 mixture of **5**^**+**^ and **6**^**+**^ from **4**^**+**^, we propose a reaction sequence commencing with solvent-mediated deprotonation of **9** to give neutral platinum(I) cyclometalated complex [Pt(P*t*Bu_2_CMe_2_CH_2_)(P*t*Bu_3_)] (**10**) (Figure 4; solvent modeled as a THF dimer). Oxidation of **10** by **4**^**+**^ would thereafter give **5**^**+**^, with the reduced product **2** capturing the proton to afford **6**^**+**^. This suggestion is consistent with the product stoichiometry, absence of H/D exchange when conducted in *d*_8_-THF, known reduction potential of **5**^**+**^ (*E*_1/2_ = −1.90 V relative to Fc/Fc^+^),^14^ and synthetic procedures used for preparing **5**^**+**^ and **6**^**+**^ from **2** (Scheme 3). Computational analysis suggests that deprotonation of **9** is the rate-determining step, conferring an overall activation barrier of Δ*G*^⧧^_298K_ = 32.0 kcal·mol^–1^ with respect to **4**^**+**^. When excess THF is factored in, this barrier is lowered to Δ*G*^⧧^_298K_(20 mM **4**^**+**^ in THF) = 24.3 kcal·mol^–1^.

### Isolation and Stability of Tri(1-adamantyl)phosphine-Ligated Metalloradicals

Recognizing the requirement for C-atom planarization in carbon-to-metal H-atom transfer reactions, we speculated that phosphine ligands with caged substituents would be less susceptible to cyclometalation and therefore confer enhanced metalloradical stability in solution. Tri(1-adamantyl)phosphine (PAd_3_) is well suited to test this conjecture and is notable for a similar steric profile to P*t*Bu_3_ about the metal (%*V*_bur_ = 40.5 cf. 40.0%), but appreciably stronger donor characteristics (TEP = 2052.1, cf. 2056.1 cm^–1^).^42^ The bis(phosphine) Au(I) complex [Au(PAd_3_)_2_]^+^ is a notable diamagnetic derivative,^43^ and the nickel metalloradical [Ni(PAd_3_)_2_]^+^ has recently been reported.^10^

**Scheme 5:**
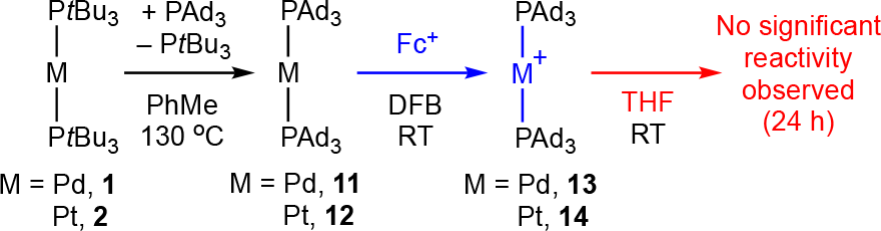
Synthesis and Stability of [M(PAd_3_)_2_][BAr^F^_4_] [BAr^F^_4_]^−^ counterion omitted for clarity.

Exploiting the relative donor strength of the phosphine ligands, zerovalent PAd_3_ complexes [M(PAd_3_)_2_] (M = Pd, **11**;^44^ Pt, **12**) were obtained as analytically pure white powders from ligand substitution reactions of the P*t*Bu_3_ analogues **1** and **2** in toluene (>80% isolated yields, Scheme 5). These neutral complexes are highly insoluble in common organic solvents (including CH_2_Cl_2_, THF, PhMe, and DFB), presumably resulting from abnormally strong intermolecular dispersion forces,^45^ but the target and considerably more soluble metalloradical derivatives [M(PAd_3_)_2_][BAr^F^_4_] (M = Pd, **13**; Pt, **14**) were obtained in >80% yield by treatment of suspensions of **11** and **12** in DFB with Fc[BAr^F^_4_] and extensively characterized. While the insolubility of **11** and **12** prevented direct measurement of the M(0)/M(I) redox potentials by cyclic voltammetry, these values can be estimated from the half-peak potentials for the reduction of **13** and **14**, *E*_P/2_ = −0.70 and −0.33 V, respectively (DFB/[*n*Bu_4_N][BAr^F^_4_], relative to Fc/Fc^+^).^46,47^ These values are ca. 0.15 V more anodic than the corresponding P*t*Bu_3_ systems under equivalent conditions (*E*_1/2_ = −0.50 V, **1**/**3**^**+**^; −0.19 V, **2**/**4**^**+**^; DFB/[*n*Bu_4_N][BAr^F^_4_], relative to Fc/Fc^+^), congruent with installation of stronger phosphine donors.

Very broad paramagnetically shifted adamantyl resonances are observed by ^1^H NMR spectroscopy for both new metalloradicals in DFB solution. No ^31^P NMR resonance was observed for **13** between +500 and −500 ppm, but a broad signal can be identified for **14** at δ −252.4 (fwhm = 120 Hz), upfield of that observed for **4**[BAr^F^_4_] (δ −213.9). Analysis of **13** by EPR spectroscopy confirms the assignment as a metal-centered radical, with observation of a singlet resonance arising from an axial *g*-tensor, *g*_⊥_*=* 2.333 and *g*_∥_ = 1.979, that is superimposed with a lower intensity sextet arising from isotropic hyperfine coupling to ^105^Pd (*I* = 5/2, 22% abundance, *a* = 24.6 mT; DFB glass @ 200 K). The magnitude of the hyperfine coupling constant is similar to that recorded for **3**[BAr^F^_4_], implying only small changes in the character of the singly occupied molecular orbital. Weak shoulders on the 338 mT hyperfine line could be an indication of an unresolved superhyperfine interaction, but this was not modeled. As for **4**[BAr^F^_4_], no metal-centered EPR spectrum was observed for **14** down to 100 K (DFB glass). The UV–vis spectra of the new metalloradicals (blue, **13**; green, **14**) are comparable to those of the respective P*t*Bu_3_ analogues, with the main bands slightly red-shifted.

**Figure 5:**
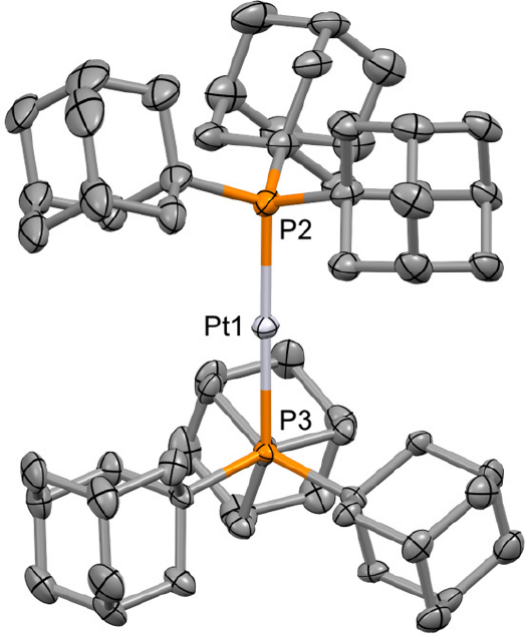
Solid-state structure of **14**. Anisotropic displacement ellipsoids drawn at 30% probability; hydrogen atoms, solvent molecules, and anion omitted for clarity. Selected bond lengths and angles: isomorphous **13**, Pd1–P2, 2.3383(14) Å; Pd1–P3, 2.3368(13) Å; P2–Pd1–P3, 178.94(6)°; **14**, Pt1–P2, 2.309(3) Å; Pt1–P3, 2.324(2) Å; P2–Pt1–P3, 179.29(9)°.

The formulations of **13** and **14** have been corroborated in the solid state by X-ray diffraction. The structures were obtained using samples recrystallized from DFB/hexane and are isomorphic (monoclinic *C*2/*c*) with no crystallographically imposed cation or anion symmetry (M = Pt, Figure 5).^48^ The cations are well-ordered and adopt near-ideal linear geometries (P2–M1–P3 = 178.94(6)°, **13**; 179.29(9)°, **14**) and eclipsed phosphine conformations, with the dihedral angles < 11°. Isostructural gold(I) and nickel(I) complexes also adopt this geometry in the solid state.^10,43^ In line with donor strength arguments the M–P bond lengths for **13** (2.3368(13)/2.3383(14) Å) and **14** (2.309(3)/2.324(2) Å) are on average shorter than those found in the solid state for **3**^**+**^ (avg 2.349(4) Å) and **4**^**+**^ (avg 2.336(6) Å), but the difference is not statistically significant.

As for their P*t*Bu_3_-ligated congeners, both new metalloradicals are stable in DFB with no onward reactivity observed upon standing at room temperature for 24 h. Consistent with our hypothesis, however, **13** and **14** show considerably enhanced stability in THF. The former is fully retained after 24 h in solution, while only ca. 10% decomposition was observed for the latter, in marked contrast to **4**[BAr^F^_4_], which was instantaneously converted into a 1:1 mixture of **5**[BAr^F^_4_] and **6**[BAr^F^_4_] under these conditions.

### Carbon-to-Metal H-Atom Transfer Reactions

**Scheme 6:**
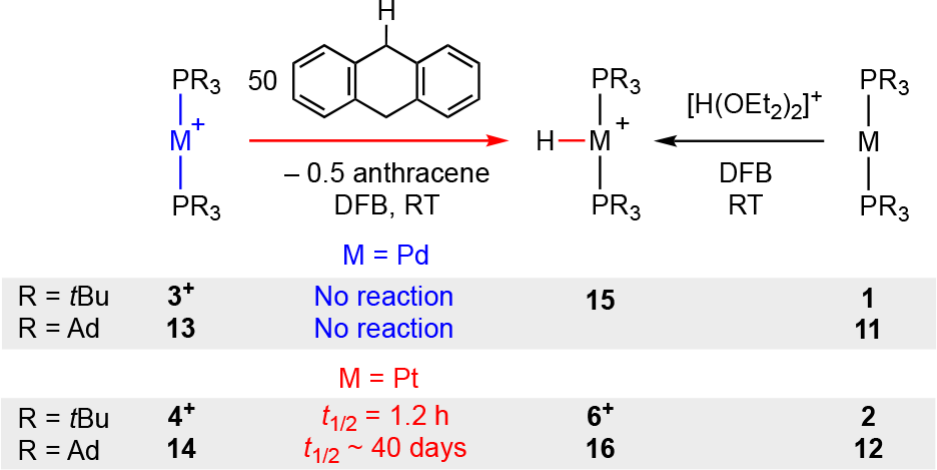
Carbon-to-Metal H-Atom Transfer Reactions of [M(PR_3_)_2_]^+^ [BAr^F^_4_]^−^ counterion omitted for clarity.

With a homologous series of isolated examples in hand we sought to systematically evaluate the propensity of late transition metal metalloradicals to activate C(sp^3^)–H bonds by carbon-to-metal H-atom transfer. Building on the precedent set by Bullock, Fujita, Grills, and co-workers using a transient osmium(I) metalloradical, **3**[BAr^F^_4_], **4**[BAr^F^_4_], **13**, and **14** were reacted with 50 equiv of 9,10-dihydroanthracene [*D*_e_(C(sp^3^)–H ∼ 77 kcal·mol^–1^] in DFB at room temperature (Scheme 6).^49^

Under these conditions, pseudo-first-order conversion of **4**[BAr^F^_4_] into **6**[BAr^F^_4_] was observed within 5 h (*t*_1/2_ = 1.2 h), with concomitant generation of *∼*0.5 equiv of anthracene by NMR spectroscopy. An equivalent carbon-to-metal H-atom transfer reaction was, however, not apparent for the palladium congener after 48 h, with only 5% decomposition of **3**[BAr^F^_4_] observed. The corresponding palladium(II) hydride [Pd(P*t*Bu_3_)_2_H][BAr^F^_4_] **15** (δ_31P_ 81.9; δ_1H_ −21.18, ^2^*J*_PH_ = 14.1 Hz) is not an inherently unstable product and was independently prepared in 80% isolated yield by reaction of **1** with [H(OEt_2_)_2_][BAr^F^_4_] at low temperature in DFB and shown to be sufficiently persistent to be observed in solution at room temperature (30% decomposition after 24 h). In line with these findings and the enhanced solution stability of the PAd_3_-ligated metalloradicals, no onward reactivity was observed in the case of **13** after 48 h, while only a very slow carbon-to-metal H-atom transfer reaction was detected for **14**. After 6 weeks, ca. 45% conversion of **12** into the new platinum(II) hydride [Pt(PAd_3_)_2_H][BAr^F^_4_] **16** (δ_31P_ 75.0, ^1^*J*_PtP_ = 2612 Hz; δ_1H_ −37.32, ^2^*J*_PH_ = 8.0 Hz, ^1^*J*_PtH_ = 2539 Hz) was observed alongside a small amount of [HPAd_3_]^+^ (*t*_1/2_ ∼ 40 days). The identity of the hydride was subsequently confirmed by independent synthesis from **12** by reaction with [H(OEt_2_)_2_][BAr^F^_4_] and obtained in 80% isolated yield (see Supporting Information for solid-state structure).

The experimental trends are well reproduced computationally using a direct H-atom transfer mechanism between the metalloradicals and 9,10-dihydroanthracene (Table 1). Reactions of the palladium complexes are characterized by an activation barrier ca. 12 kcal·mol^–1^ larger than their platinum counterparts. This difference is inversely correlated with the calculated M^II^–H bond dissociation energies, with those of palladium (64.4/64.1 kcal·mol^–1^) substantially lower than the bond dissociation energy of the C(sp^3^)–H bonds in 9,10-dihydroanthracene. The phosphine ligand substituent has a less pronounced effect, but the overall reaction kinetics and thermodynamics are less favorable for the PAd_3_-ligated metalloradicals. This difference appears to be steric in origin, with the transition states in this case characterized by smaller distortions of the P–M–P angles from linearity.

**Table 1:**
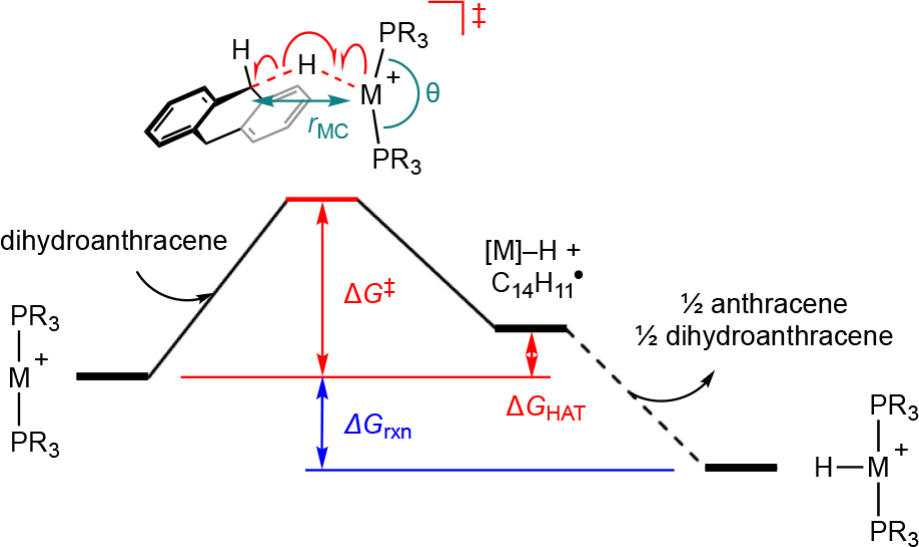
Kinetic and Thermodynamic Parameters for the Carbon-to-Metal H-Atom Transfer Reaction between [M(PR_3_)_2_]^+^ and 9,10-Dihydroanthracene (kcal·mol^–1^)^a^

**M**	**R**	**Δ*****G***^**‡**^	**Δ*****G***_**HAT**_	**Δ*****G***_**rxn**_	***r***_**MC**_ **/Å**	***θ*****/°**	***D***_**e**_**(M**^**II**^**–H)**^b^
Pd	*t*Bu	35.5	+13.0	–6.7	3.38	162.0	64.4
Pt	*t*Bu	22.7	–1.4	–21.0	3.25	160.4	78.5
Pd	Ad	35.7	+14.0	–5.6	3.32	164.0	64.1
Pt	Ad	24.2	+0.6	–19.1	3.21	161.9	77.9

a Calculated at the B2PLYP-D3(BJ)/def2-TZVPP+def-ECP(Pd,Pt)//PBEh-3c level of theory with reaction energies at 298K and corrected for DFB solvent.

b Gas phase values.

## Conclusions

One-electron oxidation of palladium(0) and platinum(0) bis(phosphine) complexes enables isolation of a homologous series of linear d^9^ metalloradicals of the form [M(PR_3_)_2_]^+^ (M = Pd, Pt; R = *t*Bu, Ad), which are stable in 1,2-difluorobenzene (DFB) solution for >1 day at room temperature when partnered with the weakly coordinating [BAr^F^_4_]^−^ (Ar^F^ = 3,5-(CF_3_)_2_C_6_H_3_) counterion. The metalloradicals exhibit reduced stability in THF solution, decreasing in the order palladium(I) > platinum(I) and PAd_3_ > P*t*Bu_3_, especially in the case of [Pt(P*t*Bu_3_)_2_]^+^ (**4**^**+**^), which was converted rapidly into a 1:1 mixture of platinum(II) metallocycle [Pt(P*t*Bu_2_CMe_2_CH_2_)(P*t*Bu_3_)]^+^ (**5**^**+**^) and platinum(II) hydride [Pt(P*t*Bu_3_)_2_H]^+^ (**6**^**+**^) upon dissolution at room temperature.

**Scheme 7:**
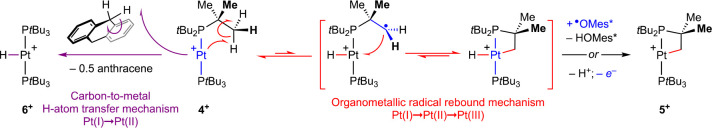
Summary of Mechanistic Proposals ^•^OMes* = 2,4,6-tri-*tert-*butylphenoxy.

Cyclometalation of **4**^**+**^ and generation of **5**^**+**^ can also be achieved by reaction with the 2,4,6-tri-*tert-*butylphenoxyl radical (^•^OMes*) in DFB and *in situ* through reaction with 2,6-di-*tert*-butyl-4-methylpyridine and ferrocenium in THF. Computational analysis of the onward reactivity of **4**^**+**^ rules out mechanisms involving Wayland-like four-center-two-electron and concerted C–H bond oxidative activation, but an energetically feasible pathway involving carbon-to-metal H-atom transfer, combination of the resulting C-centered radical with the metal, and formation of the intermediate platinum(III) hydride complex [Pt(P*t*Bu_2_CMe_2_CH_2_)H(P*t*Bu_3_)]^+^ (**9**) was identified (Scheme 7). This organometallic radical rebound mechanism reconciles the experimental observations, with conversion of **9** into **5**^**+**^ proposed to proceed by H-atom abstraction by ^•^OMes* or sequentially by solvent-mediated deprotonation of **9** to the form neutral platinum(I) complex [Pt(P*t*Bu_2_CMe_2_CH_2_)(P*t*Bu_3_)] (**10**), which can be oxidized to **5**^**+**^ by **4**^**+**^ or ferrocenium. The platinum(0) byproduct **2** or an added base thereafter mops up the solvated proton, affording platinum(II) complex **6**^**+**^ in the former case. Carbon-to-metal H-atom transfer is less accessible for the palladium(I) congener [Pd(P*t*Bu_3_)_2_]^+^ (**3**^**+**^), due to an inherently weaker M^II^–H bond, and the PAd_3_-ligated metalloradicals, where the caged phosphine substituents encumber C-atom planarization.

Reactions of the metalloradicals with 9,10-dihydroanthracene in DFB at room temperature provide direct experimental evidence for carbon-to-metal H-atom transfer in the case of the platinum, although conversion into platinum(II) hydride derivatives is considerably faster for [Pt(P*t*Bu_3_)_2_]^+^ (**4**^**+**^) (*t*_1/2_ = 1.2 h) than [Pt(PAd_3_)_2_]^+^ (**14**) (*t*_1/2_ ∼ 40 days). Paralleling their solution stability, the calculated barriers decrease in the order palladium(I) ≫ platinum(I) and PAd_3_ > P*t*Bu_3_, reflecting inherent periodic trends in M^II^–H bond strength and steric constraints, respectively.

These findings demonstrate (a) the synthetic accessibly of low-valent paramagnetic palladium and platinum complexes and (b) the ability of late transition metal-based radicals of this nature to activate C–H bonds in a manner that is mechanistically distinct from their more widely investigated diamagnetic counterparts. Insights of this nature may help inspire the development of new and more effective catalysts for the functionalization of C–H bonds in organic synthesis.

## Experimental Section

### 1 General Methods

All manipulations were performed under an atmosphere of argon using Schlenk and glovebox techniques unless otherwise stated. Glassware was oven-dried at 150 °C overnight and flame-dried under vacuum prior to use. Molecular sieves were activated by heating at 300 °C *in vacuo* overnight. DFB was predried over Al_2_O_3_, distilled from calcium hydride, and dried over two successive batches of 3 Å molecular sieves.^24^ THF was vacuum distilled from sodium/benzophenone and stored over 3 Å molecular sieves. CD_2_Cl_2_ was freeze–pump–thaw degassed and dried over 3 Å molecular sieves. *d*_8_-THF was dried over sodium, vacuum distilled, freeze–pump–thaw degassed, and stored over molecular sieves (3 Å). All other anhydrous solvents were purchased from Acros or Sigma-Aldrich, freeze–pump–thaw degassed, and stored over 3 Å molecular sieves. [M(P*t*Bu_3_)_2_] (M = Pd, **1**; Pt, **2**) were purchased from Sigma-Aldrich or Strem Chemicals and recrystallized from hexane before use. [*n*Bu_4_N][BAr^F^_4_],^50^ [FeCp_2_][BAr^F^_4_] (recrystallized from Et_2_O/pentane),^51^ [H(OEt_2_)_2_][BAr^F^_4_],^52^ the 2,4,6-tri-*tert*-butylphenoxyl radical,^53^ and PAd_3_^42^ were prepared using literature procedures. 2,6-Di-*tert*-butyl-4-methylpyridine was purchased from Sigma-Aldrich and used as received.

Cyclic voltammetry (CV) experiments were carried out in an inert atmosphere glovebox under argon using a PalmSens EmStat3+ Blue potentiostat and a three-electrode setup comprising a glassy carbon (CH Instruments, 3.0 mm diameter) working electrode (WE), coiled platinum wire counter electrode (CE), and silver wire quasi-reference electrode (RE). All potentials are calibrated to the ferrocene/ferrocenium (Fc/Fc^+^) redox couple, which was used as an internal standard. The half-wave potentials, *E*_1/2_, were determined from *E*_1/2_ = (*E*_P_^red^ + *E*_P_^ox^)/2, where *E*_P_^red^ and *E*_P_^ox^ are the reduction and oxidation peak potential values, respectively. For the irreversible electrochemical process, the half-peak potential, *E*_P/2_, was used as an approximation for *E*_1/2_.^47^

NMR spectra were recorded on Bruker spectrometers under argon at 298K unless otherwise stated. Chemical shifts are quoted in ppm, and coupling constants in Hz. Virtual coupling constants are reported as the separation between the first and third lines.^54^ NMR spectra in DFB and THF were recorded using an internal capillary of C_6_D_6_.^24^ EPR spectra were acquired on a Bruker EMX spectrometer using a Bruker High Sensitivity cavity (ER 4119 HS). Samples were cooled by nitrogen gas flow through a standard quartz insert from a nitrogen evaporator with a B-VT 2000 temperature control unit. To limit the dielectric loss arising from the solvent, all samples were contained in 2.2 mm i.d. quartz tubes (Wilmad 705-SQ), and the quartz insert was removed for room-temperature operation. The reported *g*-factors are referenced to a DPPH standard (*g* = 2.0036(3)).^55^ EPR data were obtained using a 200 mW microwave power at 9.51 GHz, with a 0.5 mT field modulation at 100 kHz. The simulation was performed using the pepper routine in EasySpin^56^ with the data fitted directly using a genetic algorithm in the esfit routine. Due to the high spectral width, it was necessary to remove a broad nonlinear cavity baseline prior to fitting by subtraction of a smoothed cubic spline derived from the experimental cavity background recorded under identical conditions. Convolution broadening was applied in the simulations with a combination of Lorentzian and Gaussian line width components necessary to adequately reproduce the observed spectral shape. With this phenomenological line shape model there was no further improvement in the quality of fit from allowing a nonisotropic hyperfine interaction. UV–vis spectra were recorded on an Agilent Cary 3500 UV–vis spectrometer compact Peltier system.

High-resolution (HR) ESI-MS analyses were recorded on a Bruker Maxis Impact instrument. Microanalyses were performed at the London Metropolitan University by Stephen Boyer.

### 2 Preparation of [Pd(P*t*Bu_3_)_2_][BAr^F^_4_] (**3**[BAr^F^_4_])

A solution of [FeCp_2_][BAr^F^_4_] (99.6 mg, 94.9 μmol) in DFB (5 mL) was added to a solution of [Pd(P*t*Bu_3_)_2_] (53.4 mg, 104 μmol) in DFB (5 mL), and the resulting blue solution stirred at room temperature for 15 min. Volatiles were removed *in vacuo*, and the residue was washed with hexane (3 × 5 mL) and then recrystallized from DFB/hexane at −30 °C to afford the product as ultramarine blocks. Yield: 113 mg (82.2 μmol, 87%). Spectroscopic data are consistent with literature data for **3**[PF_6_] and **3**[HCB_11_Cl_11_].^14,15^^1^H NMR (400 MHz, DFB): δ 19.3 (vbr, fwhm = 950 Hz, 54H, *t*Bu), 8.26 (br, 8H, Ar^F^), 7.62 (s, 4H, Ar^F^). ^1^H NMR (500 MHz, THF): δ 19.7 (vbr, fwhm = 940 Hz, 54H, *t*Bu), 8.27 (br, 8H, Ar^F^), 8.06 (s, 4H, Ar^F^). ^31^P{^1^H} NMR (162 MHz, DFB): No signals observed over the range δ −500 to + 500. ^31^P{^1^H} NMR (162 MHz, THF): No signals observed over the range δ −500 to + 500. UV–vis (DFB): λ_max_ 330 (br, ε = 300 M^–1^ cm^–1^), 668 (br, ε = 4700 M^–1^ cm^–1^) nm. UV–vis (THF): λ_max_ 337 (br, ε = 700 M^–1^ cm^–1^), 668 (br, ε = 4400 M^–1^ cm^–1^) nm. EPR (15 mM in DFB, 200 K): *g*_⊥_*=* 2.343, *g*_∥_ = 1.978, *a*_iso_(^105^Pd) = 25.2 mT. HR ESI-MS (positive ion, 4 kV): 511.2785 ([*M* + H]^+^, calcd 511.2818) *m*/*z*. Anal. Calcd for C_56_H_66_BF_24_P_2_Pd (1374.28 g·mol^–1^): C, 48.94; H, 4.84; N, 0.00. Found: C, 48.86; H, 4.68; N, 0.00.

### 3 Preparation of [Pt(P*t*Bu_3_)_2_][BAr^F^_4_] (**4**[BAr^F^_4_])

*Method A*: A solution of [FeCp_2_][BAr^F^_4_] (127.8 mg, 121.8 μmol) in DFB (5 mL) was added to a cooled (−10 °C) solution of [Pt(P*t*Bu_3_)_2_] (80.6 mg, 134.4 μmol) in DFB (10 mL). The resulting green solution was warmed to room temperature and stirred for 5 min. Volatiles were removed *in vacuo*, and the residue was washed with hexane (4 × 2 mL) and then recrystallized from DFB/hexane at −30 °C to afford the product as baby blue blocks. Yield: 123.0 mg (84.1 μmol, 69%).

*Method B*: A mixture of [FeCp_2_][BAr^F^_4_] (52.5 mg, 50 μmol), [Pt(P*t*Bu_3_)_2_] (31.5 mg, 52.5 μmol), and 2,6-di-*tert*-butyl-4-methylpyridine (1.03 mg, 5 μmol) was dissolved in cold DFB (4 mL, −30 °C). The resulting green solution was layered with an excess of cold hexane (−30 °C) to afford the product as baby blue crystals upon diffusion at −30 °C. Yield: 45.0 mg (30.8 μmol, 62%).

Spectroscopic data are consistent with literature data for **4**[HCB_11_Cl_11_].^15^^1^H NMR (400 MHz, DFB): δ 11.4 (vbr, fwhm = 260 Hz, 54H, *t*Bu), 8.16 (br, 8H, Ar^F^), 7.46 (s, 4H, Ar^F^). ^31^P{^1^H} NMR (162 MHz, DFB): δ −213.9 (vbr, fwhm = 90 Hz). UV–vis (DFB): λ_max_ 306 (sharp, ε = 700 M^–1^ cm^–1^), ∼320 (br shoulder, ε = 200 M^–1^ cm^–1^), 680 (ε = 200 M^–1^ cm^–1^) nm. EPR (15 mM in DFB, 100 K): observed signal not significantly different from cavity background. Anal. Calcd for C_56_H_66_BF_24_P_2_Pt (1462.95 g·mol^–1^): C, 45.98; H, 4.55; N, 0.00. Found: C, 45.92; H, 4.39; N, 0.00.

### 4 Solution Stability of **3**[BAr^F^_4_] and **4**[BAr^F^_4_]

Solutions of the metalloradicals (10.0 μmol) were prepared by dissolution in DFB or THF/*d*_8_-THF (0.5 mL) within J. Young valve NMR tubes at room temperature, and the stability/onward reactivity monitored *in situ* by NMR spectroscopy. After standing at room temperature for 24 h, no onward reactivity was apparent for **3**[BAr^F^_4_] or **4**[BAr^F^_4_] in DFB. Partial decomposition of **3**[BAr^F^_4_] (ca. 20%) was observed after standing at room temperature for 24 h in THF, while instantaneous formation of a 1:1 mixture of [Pt(P*t*Bu_2_CMe_2_CH_2_)(P*t*Bu_3_)][BAr^F^_4_] and [Pt(P*t*Bu_3_)_2_H][BAr^F^_4_] in quantitative spectroscopic yield was apparent upon dissolution of **4**[BAr^F^_4_] in THF at room temperature. The identity of these species was thereafter established *in situ* by comparison to literature values and analysis of isolated samples in THF. When this experiment was repeated in *d*_8_-THF, no D-atom incorporation was apparent by analysis of the product mixture by ^2^H NMR spectroscopy in CH_2_Cl_2_ (with 20 μL of CD_2_Cl_2_).

### 5 Preparation of [Pt(P*t*Bu_2_CMe_2_CH_2_)(P*t*Bu_3_)][BAr^F^_4_] (**5**[BAr^F^_4_])

A solution of [FeCp_2_][BAr^F^_4_] (104.9 mg, 100 μmol) in THF (2 mL) was added to a solution of [Pt(P*t*Bu_3_)_2_] (30.0 mg, 50.0 μmol) and 2,6-di-*tert*-butyl-4-methylpyridine (52.0 mg, 253 μmol) in THF (2 mL). The deep blue solution was stirred at room temperature for 36 h. Volatiles were removed *in vacuo*, and the residue was washed with hexane (3 × 5 mL) and then recrystallized from CH_2_Cl_2_/PhMe at room temperature to afford the product as pale-yellow blocks. Yield: 40.9 mg (28.0 μmol, 56%). Spectroscopic data are consistent with literature data for **5**[PF_6_].^14^^1^H NMR (500 MHz, CD_2_Cl_2_): δ 7.71–7.75 (m, 8H, Ar^F^), 7.56 (br, 4H, Ar^F^), 2.74 (dd′, ^3^*J*_PH_ = 10.4, ^3^*J*_PH_ = 4.0, ^2^*J*_PtH_ = 110, 2H, PtCH_2_), 1.56 (d, ^3^*J*_PH_ = 13.1, 6H, P*t*Bu_2_CMe_2_CH_2_), 1.55 (d, ^3^*J*_PH_ = 14.1, 18H, P*t*Bu_2_CMe_2_CH_2_), 1.44 (d, ^3^*J*_PH_ = 13.1, 27H, P*t*Bu_3_). ^31^P{^1^H} NMR (202 MHz, CD_2_Cl_2_): δ 59.5 (d′, ^2^*J*_PP_ = 316, ^1^*J*_PtP_ = 2902, 1P, P*t*Bu_3_), 25.2 (d′, ^2^*J*_PP_ = 316, ^1^*J*_PtP_ = 1916, 1P, P*t*Bu_2_CMe_2_CH_2_). ^31^P{^1^H} NMR (162 MHz, DFB): δ 59.0 (d′, ^2^*J*_PP_ = 316, ^1^*J*_PtP_ = 2898, 1P, P*t*Bu_3_), 24.4 (d′, ^2^*J*_PP_ = 316, ^1^*J*_PtP_ = 1918, 1P, P*t*Bu_2_CMe_2_CH_2_). ^31^P{^1^H} NMR (162 MHz, THF): δ 59.1 (d′, ^2^*J*_PP_ = 317, ^1^*J*_PtP_ = 2899, 1P, P*t*Bu_3_), 25.3 (d′, ^2^*J*_PP_ = 317, ^1^*J*_PtP_ = 1915, 1P, P*t*Bu_2_CMe_2_CH_2_). HR ESI-MS (positive ion, 4 kV): 598.3268 ([*M*]^+^, calcd 598.3268) *m*/*z*.

### 6 Preparation of [Pt(P*t*Bu_3_)_2_H][BAr^F^_4_] (**6**[BAr^F^_4_])

A cold (−30 °C) solution of [H(OEt_2_)_2_][BAr^F^_4_] (45.8 mg, 45.2 μmol) in DFB (2 mL) was added to a cold (−30 °C) solution of [Pt(P*t*Bu_3_)_2_] (27.1 mg, 45.2 μmol) in DFB (2 mL). The yellow solution was warmed to room temperature and stirred for 15 min. Volatiles were removed *in vacuo*, and the residue was recrystallized from DFB/hexane at −30 °C to afford the product as yellow blocks. Yield: 51.0 mg (34.8 μmol, 77%). Spectroscopic data are consistent with literature data.^26^ This complex is stable in DFB solution at room temperature for 24 h. ^1^H NMR (400 MHz, CD_2_Cl_2_): δ 7.70–7.75 (m, 8H, Ar^F^), 7.56 (br, 4H, Ar^F^), 1.51 (vt, *J*_PH_ = 13.2, 54H, *t*Bu), −36.53 (t′, ^2^*J*_PH_ = 8.5, ^1^*J*_PtH_ = 2602 Hz, 1H, PtH). ^1^H NMR (400 MHz, DFB, selected data): δ −36.41 (t′, ^2^*J*_PH_ = 8.6, ^1^*J*_PtH_ = 2598, 1H, PtH). ^1^H NMR (400 MHz, THF, selected data): δ −35.28 (br′, ^1^*J*_PtH_ = 2540, 1H, PtH). ^31^P{^1^H} NMR (162 MHz, CD_2_Cl_2_): δ 86.9 (s′, ^1^*J*_PtP_ = 2624). ^31^P{^1^H} NMR (162 MHz, DFB): δ 86.5 (s′, ^1^*J*_PtP_ = 2623). ^31^P{^1^H} NMR (162 MHz, THF): δ 86.5 (s′, ^1^*J*_PtP_ = 2631). HR ESI-MS (positive ion, 4 kV): 600.3407 ([*M*]^+^, calcd 600.3424) *m*/*z*.

### 7 Reaction of **4**[BAr^F^_4_] with ^•^OMes*

To a J. Young valve NMR tube charged with 2,4,6-*tri*-*tert*-butylphenoxyl radical (3.14 mg, 12.0 μmol) was added a solution of **4**[BAr^F^_4_] (10.0 μmol) in DFB (0.5 mL) at room temperature, and the onward reactivity monitored *in situ* by NMR spectroscopy at room temperature. Smooth and apparent first-order conversion of **4**[BAr^F^_4_] into [Pt(P*t*Bu_2_CMe_2_CH_2_)(P*t*Bu_3_)][BAr^F^_4_] with ca. 90% selectivity was observed over 8 days (*t*_1/2_ = 49 h).

### 8 Preparation of [Pd(PAd_3_)_2_] (**11**)

A suspension of [Pd(P*t*Bu_3_)_2_] (23.7 mg, 46.4 μmol) and PAd_3_ (60.8 mg, 139 μmol) in PhMe (5 mL) was heated at 130 °C for 18 h. The precipitate was isolated by filtration at room temperature, washed with PhMe (3 × 2 mL) and hexane (5 × 1 mL), and dried *in vacuo* to afford the product as a fine colorless powder. Yield: 40.4 mg (41.2 μmol, 89%). Acquisition of NMR data was not possible due to the insolubility of this complex. An alternative procedure has been deposited in a preprint archive.^44^ Anal. Calcd for C_60_H_90_P_2_Pd (979.75 g·mol^–1^): C, 73.56; H, 9.26. Found: C, 73.36; H, 9.17.

### 9 Preparation of [Pt(PAd_3_)_2_] (**12**)

A suspension of [Pt(P*t*Bu_3_)_2_] (180 mg, 300 μmol) and PAd_3_ (288 mg, 660 mmol) in PhMe (5 mL) was heated at 130 °C for 18 h. The precipitate was isolated by filtration at room temperature, washed with PhMe (3 × 5 mL), and dried *in vacuo* to afford the product as a fine colorless powder. Yield: 265 mg (248 μmol, 83%). Acquisition of NMR data was not possible due to the insolubility of this complex. Anal. Calcd for C_60_H_90_P_2_Pt (1068.41 g·mol^–1^): C, 67.45; H, 8.49. Found: C, 67.50; H, 8.62.

### 10 Preparation of [Pd(PAd_3_)_2_][BAr^F^_4_] (**13**)

A suspension of [Pd(PAd_3_)_2_] (99.2 mg, 101 μmol) and [FeCp_2_][BAr^F^_4_] (103 mg, 98.1 μmol) in DFB (5 mL) was vigorously stirred at room temperature for 15 min. Volatiles were removed *in vacuo*, and the residue was washed with hexane (3 × 1 mL) and then extracted with DFB (3 × 1 mL). The combined DFB extracts were layered with excess hexane to afford the product as turquoise blocks upon diffusion at −30 °C. Yield: 167 mg (90.6 μmol, 92%). ^1^H NMR (400 MHz, DFB): δ 8.27 (br, 8H, Ar^F^), 7.63 (s, 4H, Ar^F^), 4.64 (vbr, fwhm = 140 Hz, 18H, Ad{3-CH}), 1.66 (vbr, fwhm = 230 Hz, 36H, Ad{4-CH_2_}). The 2-Ad signal was not observed over the range −50 to +50. ^1^H NMR (400 MHz, THF): δ 8.23 (br, 8H, Ar^F^), 8.00 (s, 4H, Ar^F^), 5.11 (vbr, fwhm = 130 Hz, 18H, Ad{3-CH}). The 4-Ad signal is obscured by the solvent and the 2-Ad signal was not observed over the range −50 to +50. ^13^C{^1^H} NMR (126 MHz, DFB): δ 162.7 (q, ^1^*J*_CB_ = 50, Ar^F^), 135.3 (s, Ar^F^), 129.9 (qq, ^2^*J*_FC_ = 32, ^2^*J*_CB_ = 3, Ar^F^), 125.1 (q, ^1^*J*_FC_ = 272, Ar^F^), 117.8 (br, Ar^F^). No Ad signals observed. ^31^P{^1^H} NMR (162 MHz, DFB): No signals observed over the range δ −500 to +500. ^31^P{^1^H} NMR (162 MHz, THF): No signals observed over the range δ −500 to +500. UV–vis (DFB): λ_max_ 310 (ε = 800 M^–1^ cm^–1^), 694 (ε = 3100 M^–1^ cm^–1^) nm. EPR (15 mM in DFB, 200 K): *g*_⊥_*=* 2.333, *g*_∥_ = 1.979, *a*_iso_(^105^Pd) = 24.6 mT. HR ESI-MS (positive ion, 4 kV): 978.5568 ([*M*]^+^, calcd 978.5568) *m*/*z*. Anal. Calcd for C_92_H_102_BF_24_P_2_Pd (1842.97 g·mol^–1^): C, 59.96; H, 5.58; N, 0.00. Found: C, 60.04; H, 5.69; N, 0.00.

### 11 Preparation of [Pt(PAd_3_)_2_][BAr^F^_4_] (**14**)

A suspension of [Pt(PAd_3_)_2_] (53.4 mg, 50.0 μmol) and [FeCp_2_][BAr^F^_4_] (47.2 mg, 45.0 μmol) in DFB (2 mL) was vigorously stirred at room temperature for 15 min. Volatiles were removed *in vacuo*, and the residue was washed with hexane (3 × 1 mL) and then extracted with DFB (3 × 1 mL). The combined DFB extracts were layered with excess hexane to afford the product as malachite blocks in >99% purity upon diffusion at −30 °C. Yield: 71.3 mg (36.9 μmol, 82%). Trace amounts of [HPAd_3_][BAr^F^_4_] can be observed, but removed by recrystallization from CH_2_Cl_2_/heptane. ^1^H NMR (400 MHz, DFB): δ 8.28 (br, 8H, Ar^F^), 7.63 (s, 4H, Ar^F^). A continuous broad resonance in the baseline is observed over the range δ −5 to 15 and attributed to Ad resonances. ^1^H NMR (400 MHz, THF): Only anion signals observed. ^13^C{^1^H} NMR (126 MHz, DFB): δ 162.8 (q, ^1^*J*_CB_ = 50, Ar^F^), 135.3 (s, Ar^F^), 129.9 (qq, ^2^*J*_FC_ = 32, ^2^*J*_CB_ = 3, Ar^F^), 125.0 (q, ^1^*J*_FC_ = 272, Ar^F^), 117.8 (sept., ^3^*J*_FC_ = 4, Ar^F^). No Ad signals observed. ^31^P{^1^H} NMR (162 MHz, DFB): δ −252.4 (vbr, fwhm = 120 Hz). ^31^P{^1^H} NMR (162 MHz, THF): δ −245.2 (vbr, fwhm = 95 Hz). UV–vis (DFB): λ_max_ 309 (sharp, ε = 1100 M^–1^ cm^–1^), ∼325 (br shoulder, ε = 700 M^–1^ cm^–1^), 703 (ε = 200 M^–1^ cm^–1^) nm. EPR (15 mM in DFB, 100 K): observed signal not significantly different from cavity background. HR ESI-MS (positive ion, 4 kV): 1068.6242 ([*M*]^+^, calcd 1068.6244) *m*/*z*. Anal. Calcd for C_92_H_102_BF_24_P_2_Pt (1931.63 g·mol^–1^): C, 57.21; H, 5.32; N, 0.00. Found: C, 57.40; H, 5.35; N, 0.00.

### 12 Solution Stability of **13** and **14**

Solutions were prepared by dissolution of the metalloradicals (10.0 μmol) in DFB or THF (0.5 mL) within J. Young valve NMR tubes at room temperature, and thereafter the stability/onward reactivity was analyzed *in situ* by NMR spectroscopy. After standing at room temperature for 24 h, no onward reactivity was apparent for **13** and **14** in DFB and **13** in THF. Partial decomposition of **14** (10%) was observed after standing at room temperature for 24 h in THF, generating a mixture containing [Pt(PAd_3_)_2_H][BAr^F^_4_] and [HPAd_3_][BAr^F^_4_]. The former was subsequently confirmed by independent synthesis.

### 13 Reactions of **3**[BAr^F^_4_], **4**[BAr^F^_4_], **13**, and **14** with 9,10-Dihydroanthracene

Solutions of **3**[BAr^F^_4_], **4**[BAr^F^_4_], **13**, or **14** (10.0 μmol) in DFB (0.5 mL) were added into a J. Young valve NMR tube charged with 9,10-dihydroanthracene (92.0 mg, 500 μmol) and the resulting solutions analyzed *in situ* by NMR spectroscopy over time at room temperature. There was no evidence for the formation of anthracene or hydride derivatives after 48 h for both palladium metalloradicals, but ca. 5% decomposition of **3**[BAr^F^_4_] was observed over this time frame. Pseudo-first-order conversion of **4**[BAr^F^_4_] into [Pt(P*t*Bu_3_)_2_H][BAr^F^_4_] was observed within 5 h (*t*_1/2_ = 1.2 h), alongside ca. 0.4 equiv of anthracene. Trace amounts of anthracene and [Pt(PAd_3_)_2_H][BAr^F^_4_] were observed after 48 h in the case of **14**, the identity of which was subsequently confirmed by independent synthesis. After 6 weeks in solution, ca. 45% conversion of **14** (*t*_1/2_ ∼ 40 days) into [Pt(PAd_3_)_2_H][BAr^F^_4_] was observed alongside anthracene and a small amount of [HPAd_3_][BAr^F^_4_].

### 14 Preparation of [Pd(P*t*Bu_3_)_2_H][BAr^F^_4_] (**15**)

A cold (−30 °C) solution of [H(OEt_2_)_2_][BAr^F^_4_] (45.8 mg, 45.2 μmol) in DFB (1 mL) was added to a cold (−30 °C) solution of [Pd(P*t*Bu_3_)_2_] (22.9 mg, 44.8 μmol) in DFB (2 mL). The yellow solution was stirred for 5 min, then layered with excess cold hexane to afford the product as yellow blocks upon diffusion at −30 °C. Yield: 49.0 mg (35.6 μmol, 80%). Partial decomposition (30%) of this complex was observed after standing in DFB solution at room temperature for 24 h. ^1^H NMR (500 MHz, DFB): δ 8.13 (br, 8H, Ar^F^), 7.49 (s, 4H, Ar^F^), 1.28 (vt, *J*_PH_ = 13.6, 54H, *t*Bu), −21.18 (t, ^2^*J*_PH_ = 14.1, 1H, PdH). ^13^C{^1^H} NMR (126 MHz, DFB): δ 162.6 (q, ^1^*J*_CB_ = 50, Ar^F^), 135.1 (s, Ar^F^), 129.7 (qq, ^2^*J*_FC_ = 32, ^2^*J*_CB_ = 3, Ar^F^), 124.9 (q, ^1^*J*_FC_ = 273, Ar^F^), 117.6 (sept., ^3^*J*_FC_ = 4, Ar^F^), 39.0 (vt, *J*_PC_ = 12, *t*Bu{C}), 31.6 (vt, *J*_PC_ = 5, *t*Bu{CH_3_}). ^31^P{^1^H} NMR (162 MHz, DFB): δ 81.9 (s). HR ESI-MS (positive ion, 4 kV): 511.2798 ([*M*]^+^, calcd 511.2818) *m*/*z*. Anal. Calcd for C_56_H_67_BF_24_P_2_Pd (1375.29 g·mol^–1^): C, 48.91; H, 4.91; N, 0.00. Found: C, 48.98; H, 4.84; N, 0.00.

### 15 Preparation of [Pt(PAd_3_)_2_H][BAr^F^_4_] (**16**)

A cold (−30 °C) solution of [H(OEt_2_)_2_][BAr^F^_4_] (20.6 mg, 20.3 μmol) in DFB (1 mL) was added to a cold (−30 °C) suspension of [Pt(PAd_3_)_2_] (22.0 mg, 20.6 μmol) in DFB (1 mL). The yellow suspension was warmed to room temperature, stirred for 18 h, and filtered, and the precipitate extracted with additional DFB (3 × 0.5 mL). The combined filtrate and washings were layered with excess hexane, and the product was obtained as yellow blocks upon diffusion at −30 °C. Yield: 31.4 mg (16.3 μmol, 80%). This complex is stable in DFB solution at room temperature for 24 h. ^1^H NMR (400 MHz, DFB): δ 8.11 (br, 8H, Ar^F^), 7.48 (s, 4H, Ar^F^), 2.37 (br, 36H, Ad{2-CH_2_}), 1.94 (s, 18H, Ad{3-CH}), 1.60–1.73 (m, 36H, 2 × Ad{4-CH_2_}), −37.32 (t′, ^2^*J*_PH_ = 8.0, ^1^*J*_PtH_ = 2539,1H, PtH). ^1^H NMR (400 MHz, THF, selected data): δ −36.58 (t′, ^2^*J*_PH_ = 7.8, ^1^*J*_PtH_ = 2531,1H, PtH). ^13^C{^1^H} NMR (126 MHz, DFB): δ 162.6 (q, ^1^*J*_CB_ = 50, Ar^F^), 135.1 (s, Ar^F^), 129.7 (qq, ^2^*J*_FC_ = 32, ^2^*J*_CB_ = 3, Ar^F^), 124.8 (q, ^1^*J*_FC_ = 272, Ar^F^), 117.6 (sept., ^3^*J*_FC_ = 4, Ar^F^), 48.9 (vt, *J*_PC_ = 13, Ad{1-C}), 43.3 (br, Ad{2-CH_2_}), 36.2 (s, Ad{4-CH_2_}), 29.4 (vt, *J*_PC_ = 8, Ad{3-CH}). ^31^P{^1^H} NMR (162 MHz, DFB): δ 75.0 (s′, ^1^*J*_PtP_ = 2612). ^31^P{^1^H} NMR (162 MHz, THF): δ 75.2 (s′, ^1^*J*_PtP_ = 2609). HR ESI-MS (positive ion, 4 kV): 1068.6244 ([*M*]^+^, calcd 1068.6244) *m*/*z*. Anal. Calcd for C_92_H_103_BF_24_P_2_Pt (1932.64 g·mol^–1^): C, 57.18; H, 5.37; N, 0.00. Found: C, 57.26; H, 5.18; N, 0.00.

### 16 Computational Details

All electronic structure calculations presented in this paper were carried out using the ORCA 5.0.3 program package.^57^ To find energetically most favorable conformers, initial conformer searches of selected complexes were conducted using the automated *crest* approach,^58^ which employs the efficient semiempirical extended tight binding method (GFN2-xTB) with a specially adapted implicit solvation model (ALPB).^59^ Unconstrained geometry optimizations in *C*_1_ symmetry and analytical (or numerical for the largest systems) frequency calculations of all compounds were carried out at the DFT level, using the PBEh-3c composite method.^30^ The orbitals are expanded in a modified valence double-ζ Gaussian basis corresponding to the Ahlrichs-type def2-mSVP set, in conjunction with the def2/J auxiliary basis set for the RI approximation to the Coulomb term.^60^ The calculations utilized the def2-ECP for Pd (replacing 28 core electrons) and Pt (replacing 60 core electrons).^61^ To account for inter- and intramolecular basis set superposition error (BSSE) and long-range London dispersion effects, the geometrical counterpoise correction (gCP)^62^ and atom-pairwise DFT-D3 (Becke–Johnson damping) schemes are utilized.^33^ All optimized stationary points were characterized by analysis of their analytical second derivatives, with minima having only positive eigenvalues and transition states having one imaginary eigenvalue. The nature of transition states was confirmed via intrinsic reaction coordinate (IRC) calculations in both forward and reverse direction of the reaction coordinate.^63^ Subsequent geometry optimizations of the IRC end points yielded the nearest minima linked by a transition state. The frequency calculations also provided thermal and entropic corrections to the total energy in the gas phase at *T* = 298.15 K and *p* = 1 atm within the quasi rigid-rotor/harmonic oscillator (QRRHO) approximation.^64^ Open-shell singlet states (*M*_S_ = 0) corresponding to antiferromagnetically coupled metal centers were modeled with the spin-unrestricted broken-symmetry (BS) formalism.^65^ The FlipSpin feature of ORCA was used to generate initial guesses for the BS calculations. Geometries with these states were fully optimized, and convergence to the desired BS solution was confirmed by inspection of magnetic orbitals, spin populations, and the expectation value of the ⟨*S*^2^⟩ operator. The energies of the BS states were used without spin projection. Single-point energies were computed using the B2PLYP-D3(BJ) double-hybrid functional^31^ (including Grimme’s D3 atom-pairwise dispersion correction and Becke–Johnson damping) in combination with the def2-TZVPP basis set.^32^ Full details of calculations are provided in the Supporting Information. Effects due to the presence of a solvent were treated implicitly with a conductor-like polarizable continuum (CPCM) and Truhlar’s SMD model.^34^ Solvent parameters corresponded to those of tetrahydrofuran (ε = 7.4, refractive index = 1.000), or, in the absence of defined parameters for DFB solvent, default SMD parameters were selected for fluorobenzene and the dielectric constant adjusted to that of DFB (ε = 13.4, refractive index = 1.443). Geometries were visualized using the ChemCraft software package.^68^

## Supporting Information Available

NMR, EPR, UV–vis, and ESI-MS spectra of complexes and selected reactions; additional electrochemical data and analysis; further computational details, data, and analysis (PDF)
Optimized geometries of species examined computationally (XYZ)
Animations of selected transition states (MP4)

## Supplementary Material

ja3c04167_si_001.pdf

ja3c04167_si_002.xyz
