# Supplementary material for: Stability and C–H Bond Activation Reactions of Palladium(I) and Platinum(I) Metalloradicals: Carbon-to-Metal H-Atom Transfer and an Organometallic Radical Rebound Mechanism
Source: J Am Chem Soc. 2023 Jun 15;145(25):14087–100. doi: 10.1021/jacs.3c04167 (PMC10311531; doi:10.1021/jacs.3c04167)
Supplement: Supplementary file 1 — ja3c04167_si_001.pdf [file ja3c04167_si_001.pdf]

Supporting information for:

Stability and C–H Bond Activation Reactions of Palladium(I) and Platinum(I) Metalloradicals: Carbon-to-Metal H-Atom Transfer and an Organometallic Radical Rebound Mechanism

Tobias Krämer,[a,b] Matthew R. Gyton,[c] Itxaso Bustos,[c,d] Matthew J. G. Sinclair,[c] Sze-yin Tan,[c,e] Christopher J. Wedge,[f,g] Stuart A. Macgregor,[a] and Adrian B. Chaplin[c]

[a] *Institute of Chemical Sciences, Heriot-Watt University, Edinburgh EH14 4AS, UK*; [b] *Department of Chemistry, Maynooth University, Maynooth, Co. Kildare W23 F2K8, Ireland*; [c] *Department of Chemistry, University of Warwick, Gibbet Hill Road, Coventry CV4 7AL, UK*; [d] *Facultad de Química de San Sebastián, Universidad del País Vasco (UPV/EHU), Apartado 1072, 20080 San Sebastián, Spain*; [e] *Department of Chemical Engineering, Imperial College London, London SW7 2AZ, UK*; [f] *Department of Physics, University of Warwick, Gibbet Hill Road, Coventry CV4 7AL, UK*; [g] *Department of Chemical Sciences, University of Huddersfield, Queensgate, Huddersfield HD1 3DH, UK.*

Table of contents

1 Data for the Fc/Fc^+ redox couple **2**

2 Characterisation of [M(P*t*Bu_3)$_2$][BAr^F_4] (M = Pd, **3**[BAr^F_4]; Pt, **4**[BAr^F_4]) **2**

3 Solution stability of **3**[BAr^F_4] and **4**[BAr^F_4] **7**

4 Characterisation of [Pt(P*t*$Bu_2CMe_2CH_2$)(P*t*Bu_3)][BAr^F_4] (**5**[BAr^F_4]) **11**

5 Characterisation of [Pt(P*t*Bu_3)$_2$H][BAr^F_4] (**6**[BAr^F_4]) **12**

6 Reaction of **4**[BAr^F_4] with •OMes* **14**

7 Characterisation of [M(PAd_3)$_2$][BAr^F_4] (M = Pd, **13**; Pt, **14**) **15**

8 Solution stability of [M(PAd_3)$_2$][BAr^F_4] (M = Pd, **13**; Pt, **14**) **21**

9 Reactions of **3**[BAr^F_4], **4**[BAr^F_4], **13** and **14** with 9,10-dihydroanthracene **25**

10 Characterisation of [Pd(P*t*Bu_3)$_2$H][BAr^F_4] (**15**) **30**

11 Characterisation of [Pt(PAd_3)$_2$H][BAr^F_4] (**16**) **32**

12 Computational details **34**

12.1 Full details of Computational Methods 34

*12.2 Structure and properties of [M(PtBu$_3$)$_2$]$^+$ (M = Pd, **3**$^+$; Pt, **4**$^+$). 36*

*12.3 Reactions of [M(PtBu$_3$)$_2$]$^+$ (M = Pd, **3**$^+$; Pt, **4**$^+$). 40*

*12.4 Structure and properties of [M(PAd$_3$)$_2$]$^+$ (M = Pd, **13**; Pt, **14**). 57*

12.5 Reactions between [M(PR$_3$)$_2$]$^+$ and 9,10-dihydroantracene (9,10-AnH$_2$) 58

12.6 Bond dissociation energies of [M(PR$_3$)$_2$H]$^+$ 66

13 References **67**

1 Data for the Fc/Fc⁺ redox couple

CVs for the oxidation of Fc in DFB and THF solution at different scan rates are depicted below. The peak-to-peak separation values are ΔE_p = 110 mV and 78 mV at 100 mV·s^{-1} for DFB and THF, respectively. These values deviate from what is expected for reversible behaviour of the Fc/Fc^+ redox couple (60 mV) and attributed to internal resistance of the solution arising from the incomplete ionic dissociation of the supporting electrolyte.

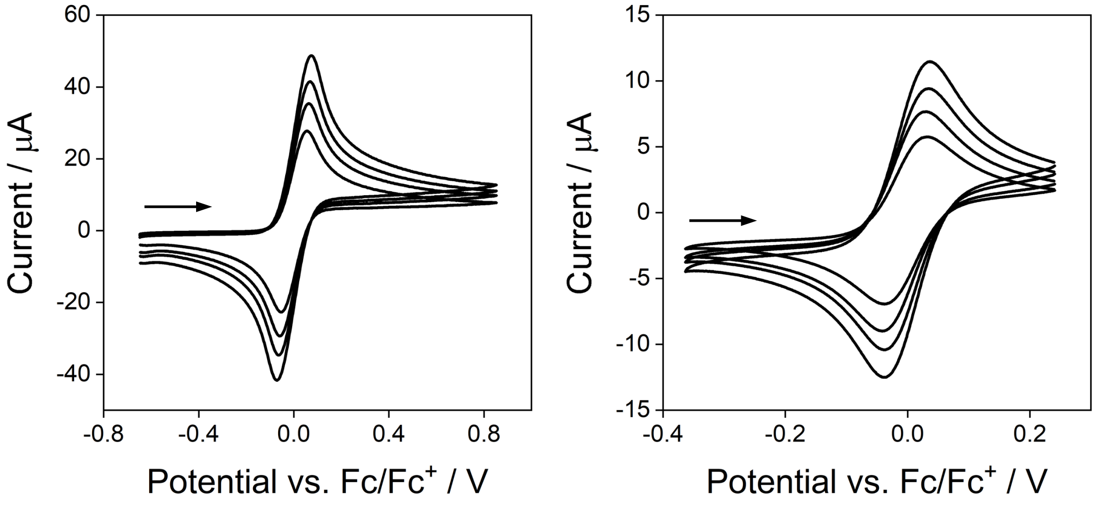

Figure S1. CVs for the oxidation of Fc in DFB (left) and THF (right): 2 mM complex; 0.2 M [nBu$_4$N][BAr$^F_4$] electrolyte; glassy carbon working electrode, coiled Pt wire counter electrode and Ag wire quasi-reference electrode; scan rates = 30, 50, 70 and 100 mV·s^{-1}).

2 Characterisation of [M(P*t*Bu$_3$)$_2$][BAr$^F_4$] (M = Pd, 3[BAr$^F_4$]; Pt, 4[BAr$^F_4$])

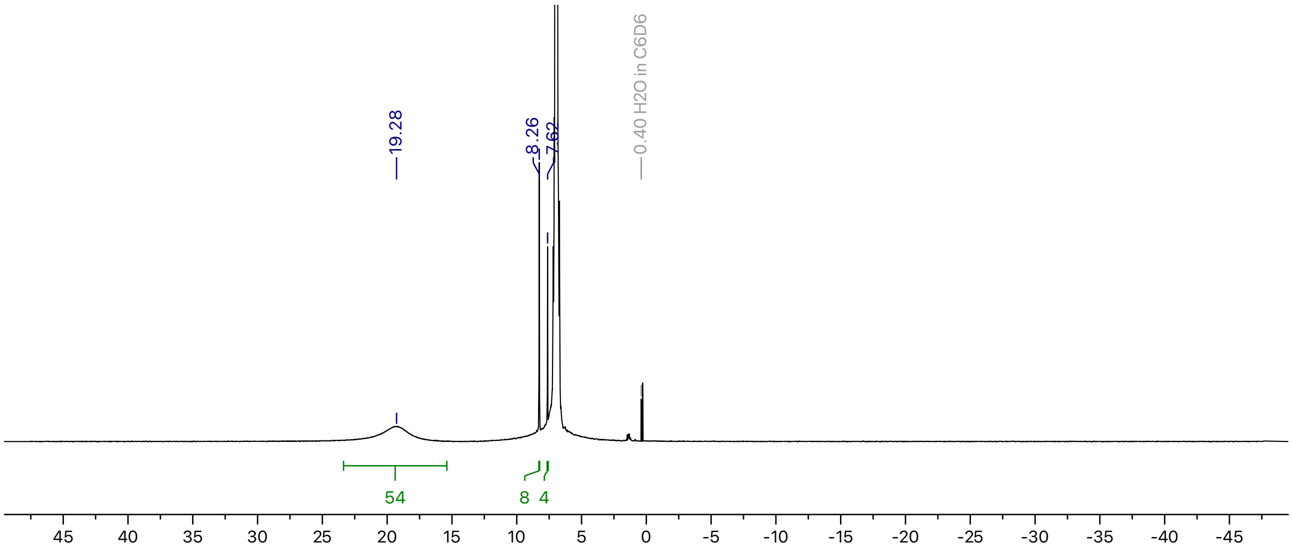

Figure S2. ^{1}H NMR spectrum of **3**[BAr$^F_4$] in DFB (400 MHz).

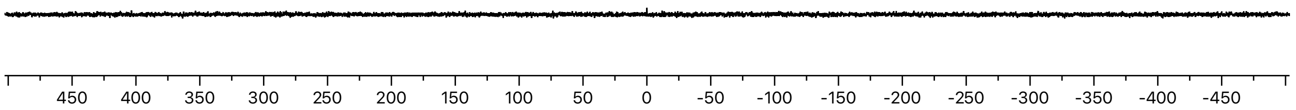

Figure S3. $^{31}P\{^1H\}$ NMR spectrum of **3**[BAr$^F{}_4$] in DFB (162 MHz).

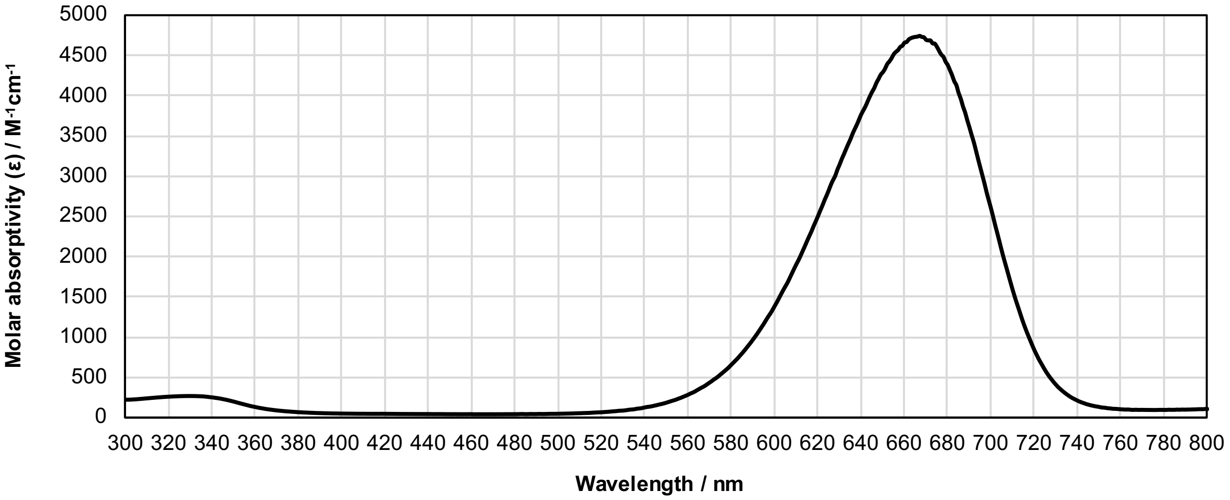

Figure S4. UV-vis spectrum of **3**[BAr$^F{}_4$] in DFB.

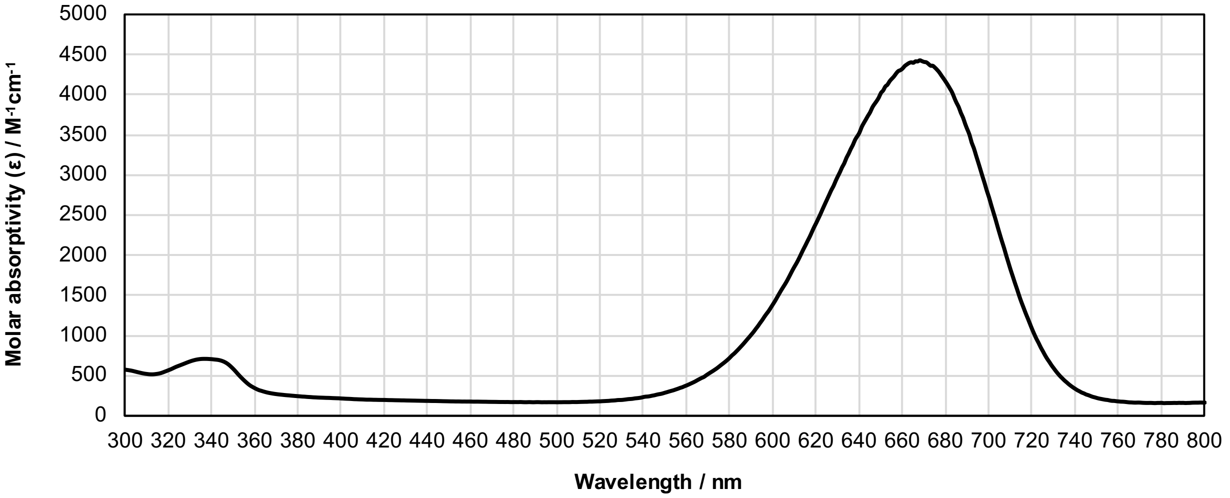

Figure S5. UV-vis spectrum of **3**[BAr$^F{}_4$] in THF.

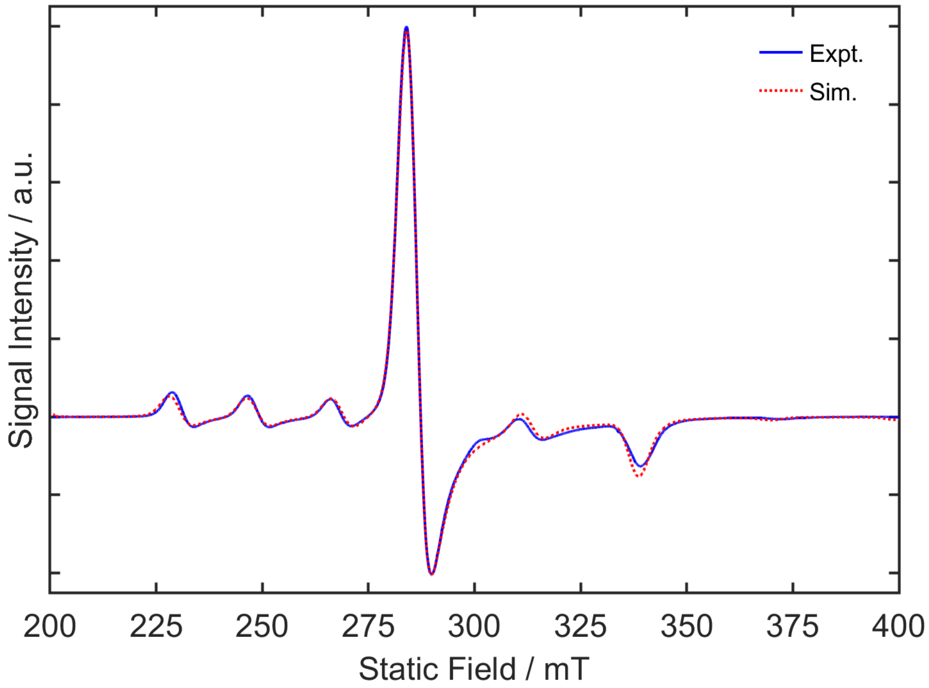

Figure S6. EPR spectrum of **3**[BAr$^F{}_4$] (DFB glass, 200 K, a.u. = arbitrary units) after baseline subtraction. Fitting gave $g_\perp$ = 2.343, $g_\parallel$ = 1.978 and A_{iso} = 25.2 mT, using phenomenological line broadenings of 5.11 mT (Gaussian) and 1.59 mT (Lorentzian).

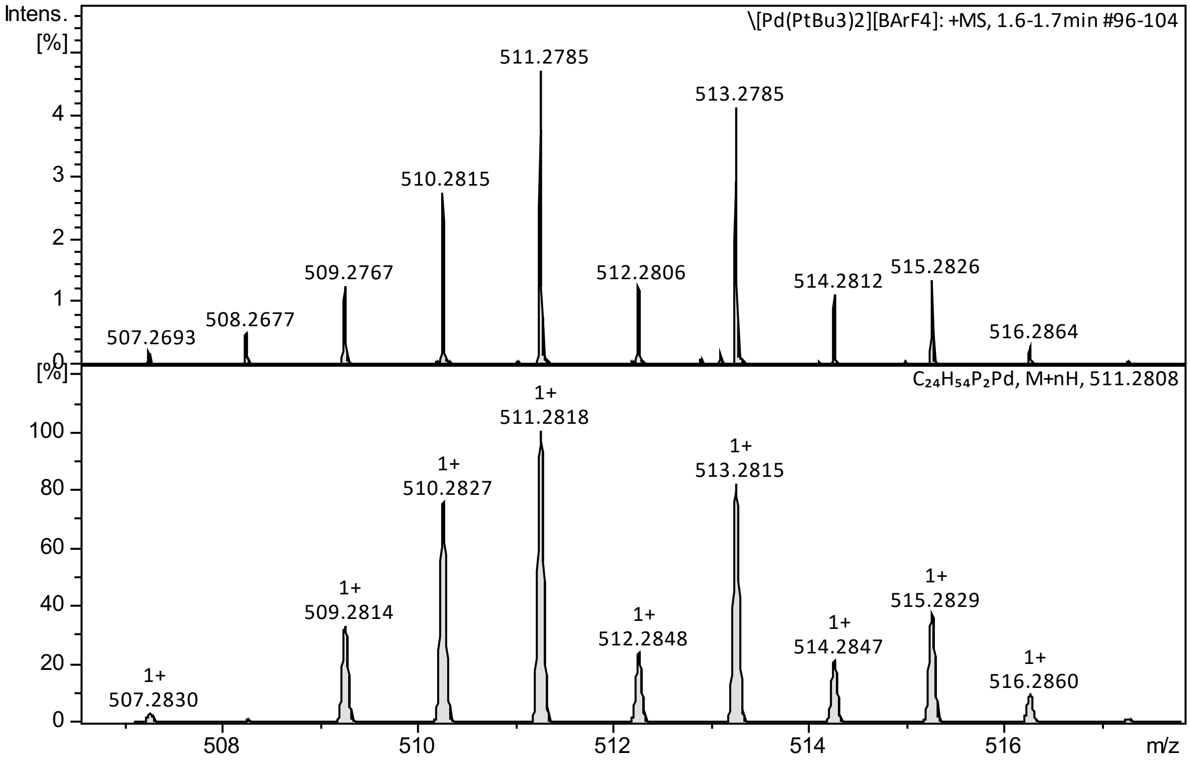

Figure S7. HR ESI-MS of **3**[BAr$^F{}_4$].

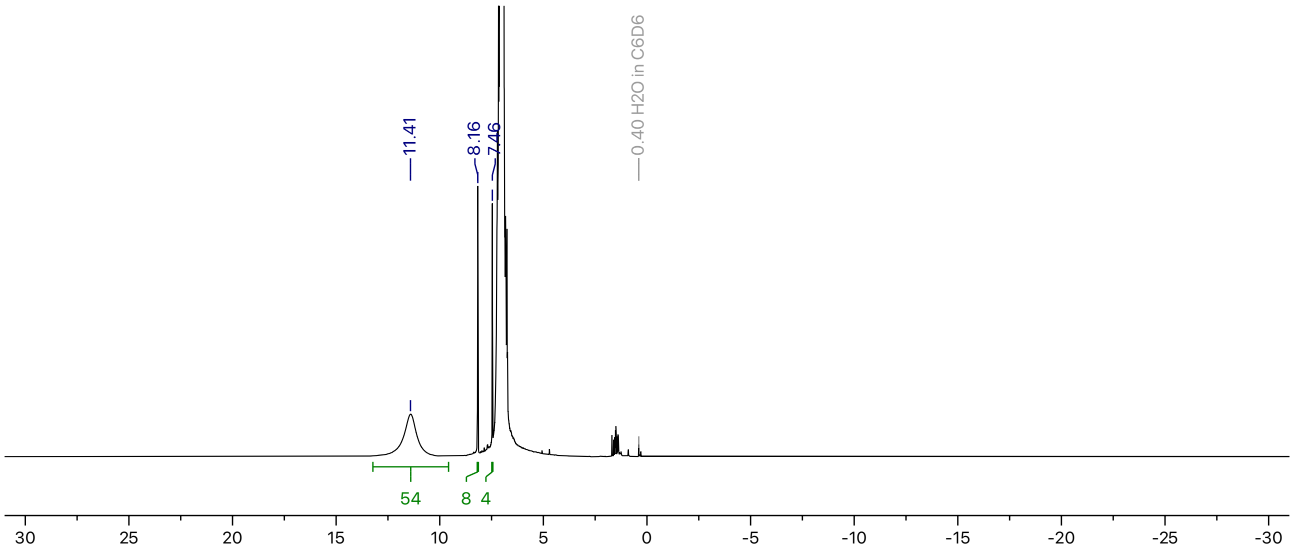

Figure S8. ^{1}H NMR spectrum of **4**[BAr$^{F}_{4}$] in DFB (400 MHz).

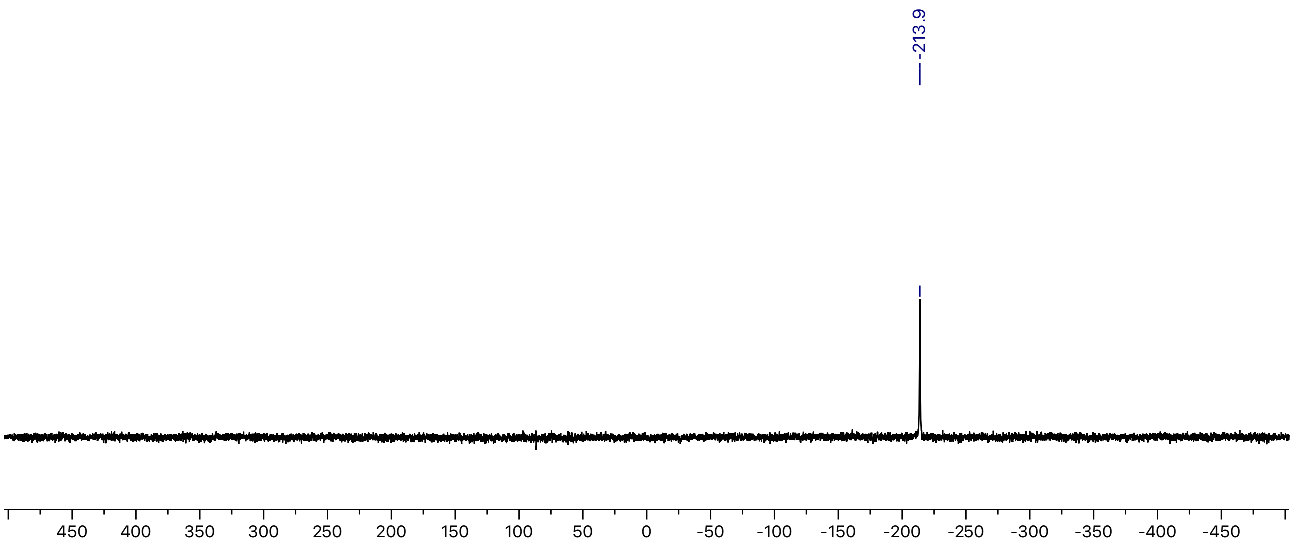

Figure S9. $^{31}P\{^{1}H\}$ NMR spectrum of **4**[BAr$^{F}_{4}$] in DFB (162 MHz).

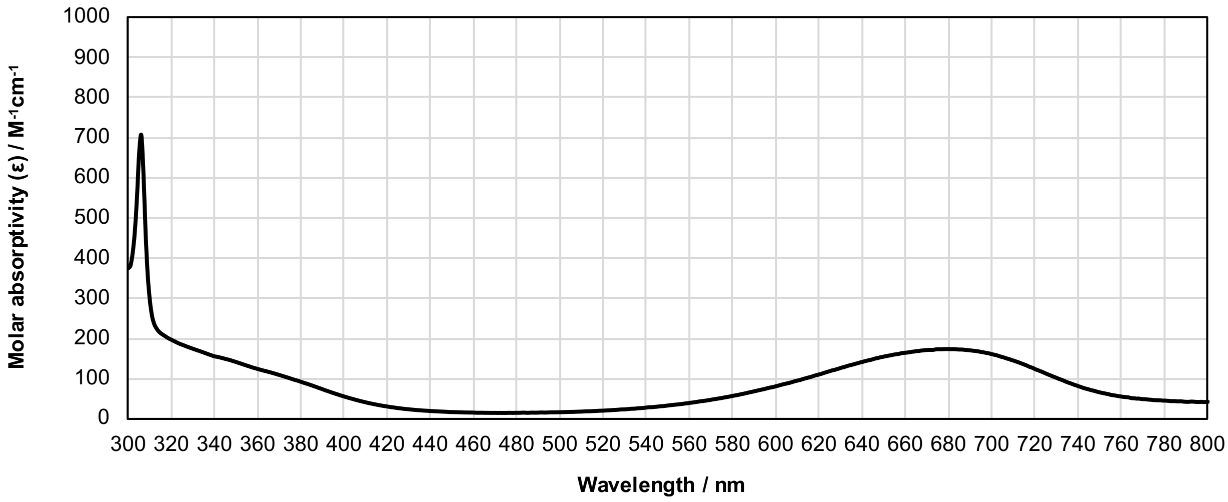

Figure S10. UV-vis spectrum of **4**[BAr$^{F}_{4}$] in DFB.

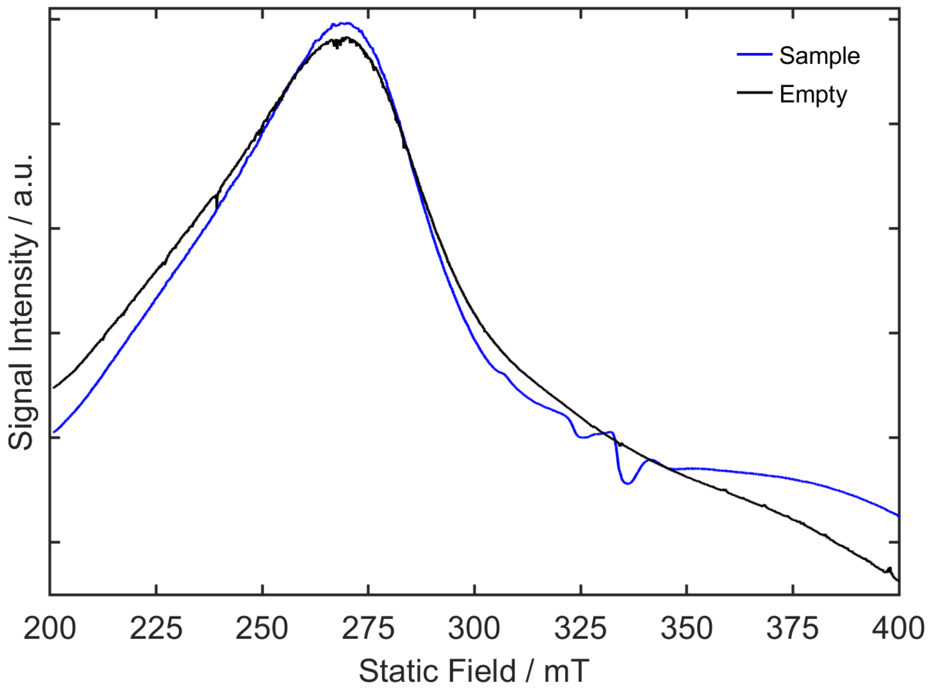

Figure S11. EPR spectrum of **4**[BAr$^F{}_4$] (DFB glass, 100 K, a.u. = arbitrary units). The spectrum of an empty sample tube recorded under identical conditions is shown for comparison, indicating the apparent signal arises only from the cavity background. The signal seen above at ~334 mT corresponds to the free electron *g*-factor and is attributed to trace quantities of an organic paramagnetic impurity.

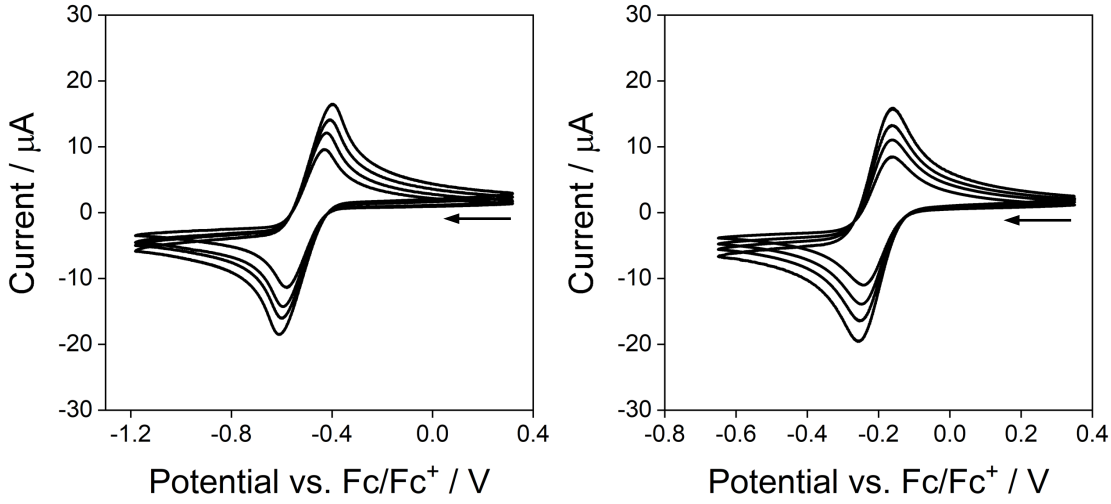

Figure S12. CVs for the reduction of **3**[BAr$^F{}_4$] (left) and **4**[BAr$^F{}_4$] (right) in DFB (2 mM complex; 0.2 M [*n*Bu$_4$N][BAr$^F{}_4$] electrolyte; glassy carbon working electrode, coiled Pt wire counter electrode and Ag wire quasi-reference electrode; scan rates = 30, 50, 70 and 100 mV·s^{-1}). $E_{1/2}$ = -0.51 V and -0.18 V, respectively. i_P^{red}/i_P^{ox} = 0.88 in both cases.

3 Solution stability of 3[BAr$^F_4$] and 4[BAr$^F_4$]

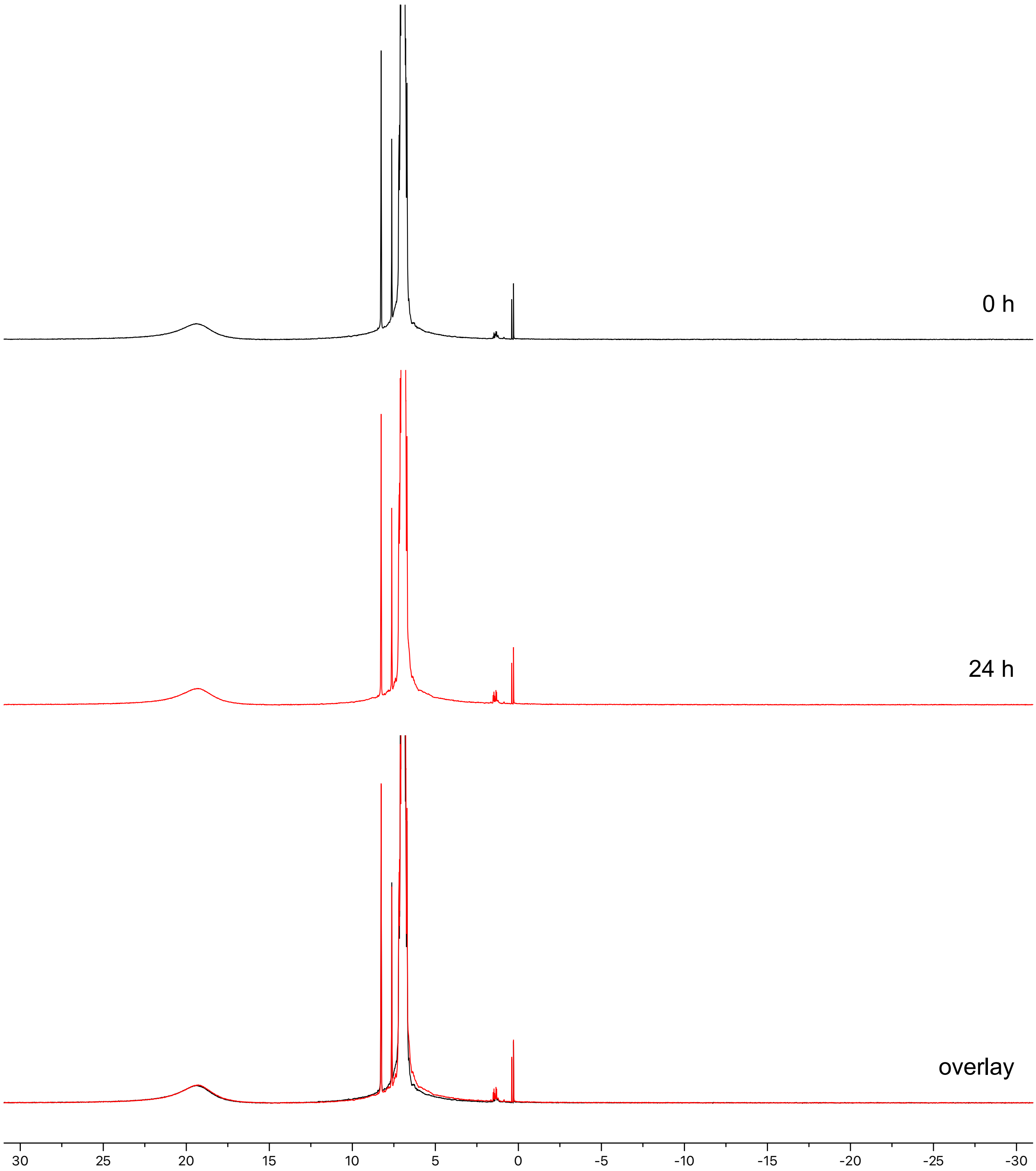

Figure S13. ^{1}H NMR spectra of **3**[BAr$^F_4$] collected over time in DFB (400 MHz).

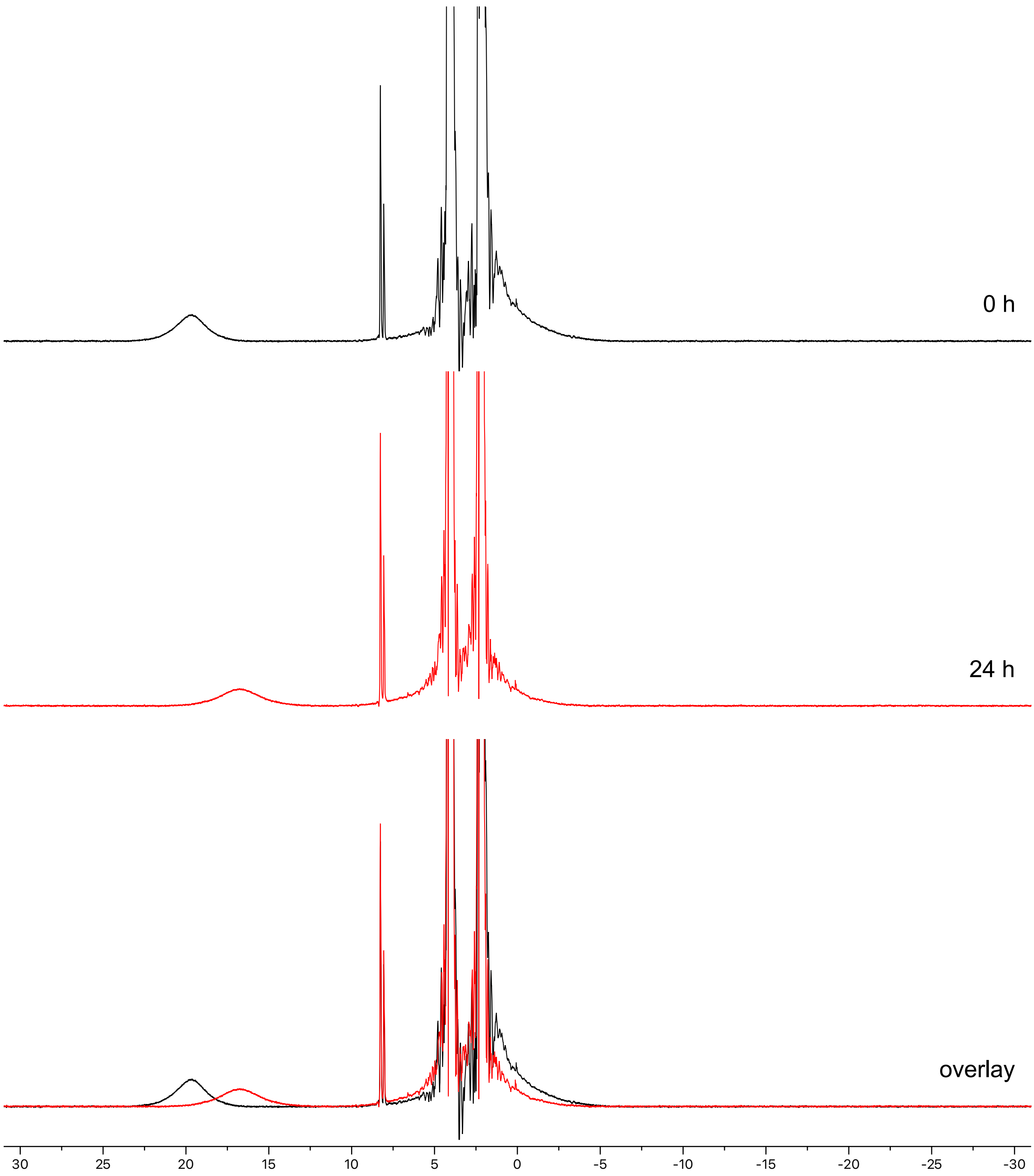

Figure S14. ^{1}H NMR spectra of **3**[BAr$^F_4$] collected after time in THF (400 MHz).

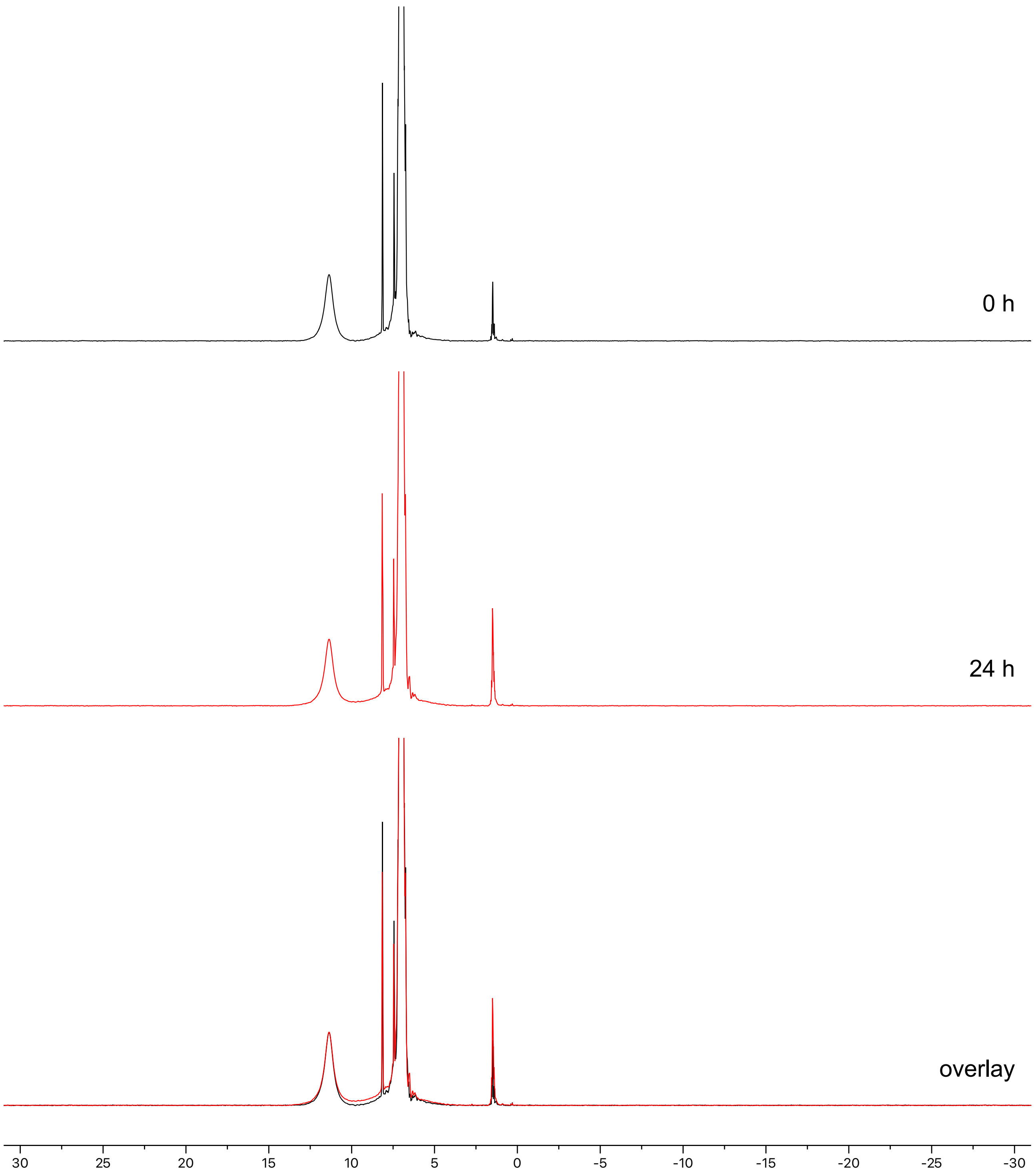

Figure S15. ^{1}H NMR spectra of **4**[BAr$^{F}_{4}$] collected over time in DFB (400 MHz).

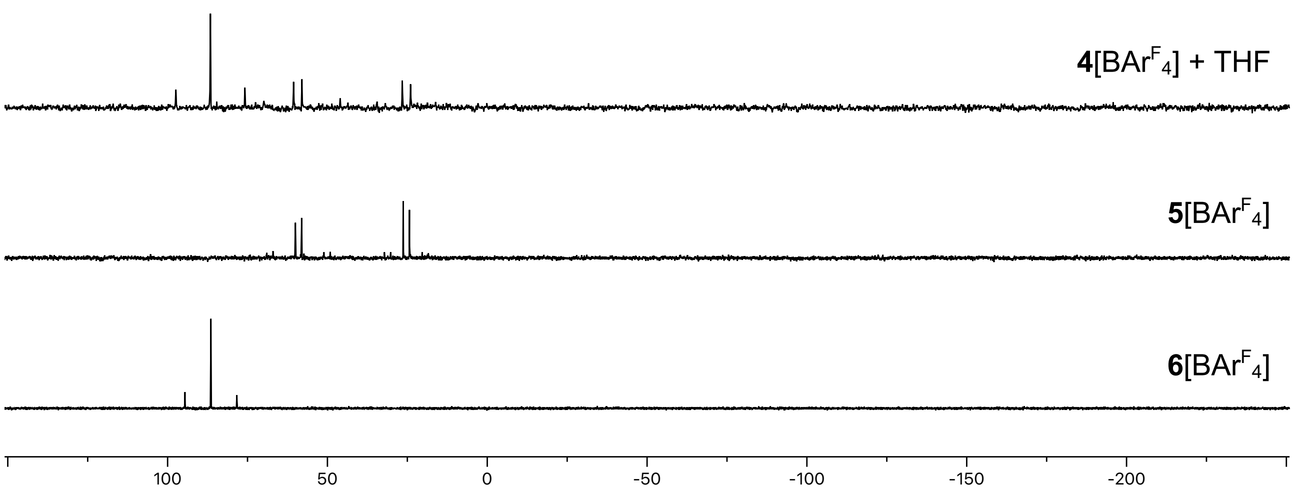

Figure S16. ^{31}P{^{1}H} NMR spectrum collected following dissolution of **4**[BAr$^{F}_{4}$] in THF and spectra of independently synthesised **5**[BAr$^{F}_{4}$] and **6**[BAr$^{F}_{4}$] (THF, 126 MHz).

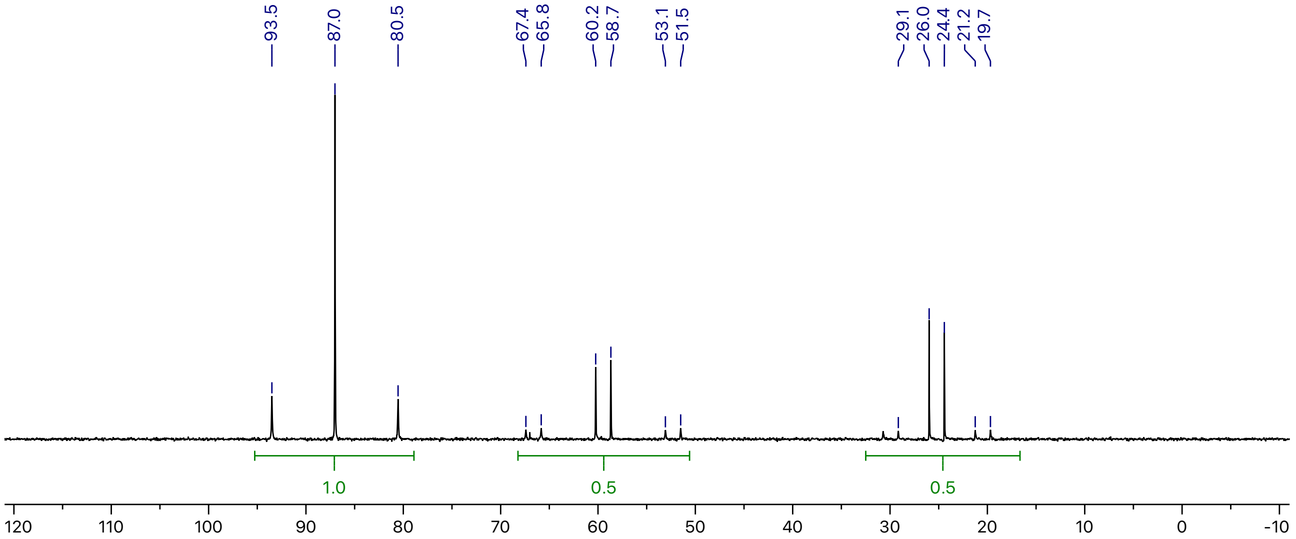

Figure S17. ^{31}P{^{1}H} NMR spectrum of the products obtained upon dissolution of **4**[BAr$^{F}_{4}$] in *d*$_{8}$-THF (CH_2Cl_2/CD_2Cl_2, 202 MHz).

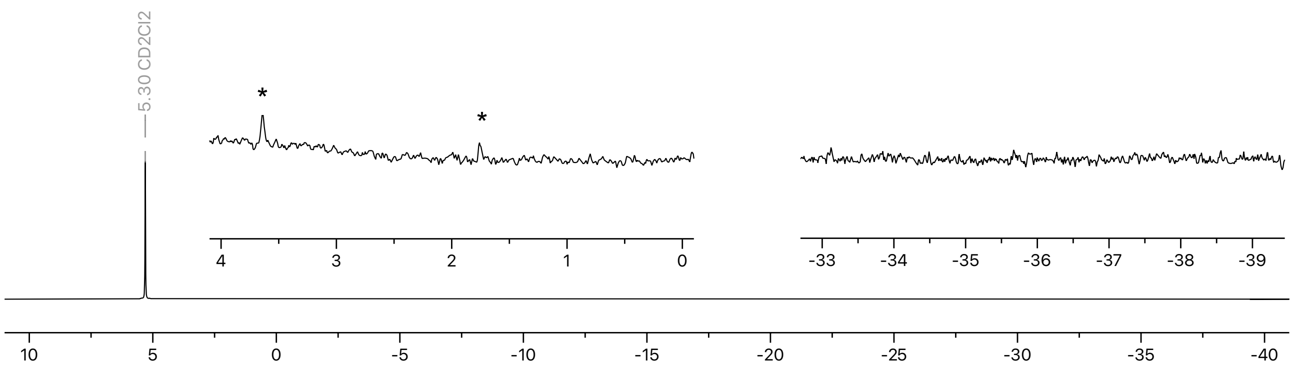

Figure S18. ^{2}H NMR spectrum of the products obtained upon dissolution of **4**[BAr$^{F}_{4}$] in *d*$_{8}$-THF. (CH_2Cl_2/CD_2Cl_2, ns = 256, 500 MHz). * = residue d^{8}-THF.

4 Characterisation of [Pt(P*t*Bu$_2$CMe$_2$CH$_2$)(P*t*Bu$_3$)][BAr^F$_4$] (5[BAr^F$_4$])

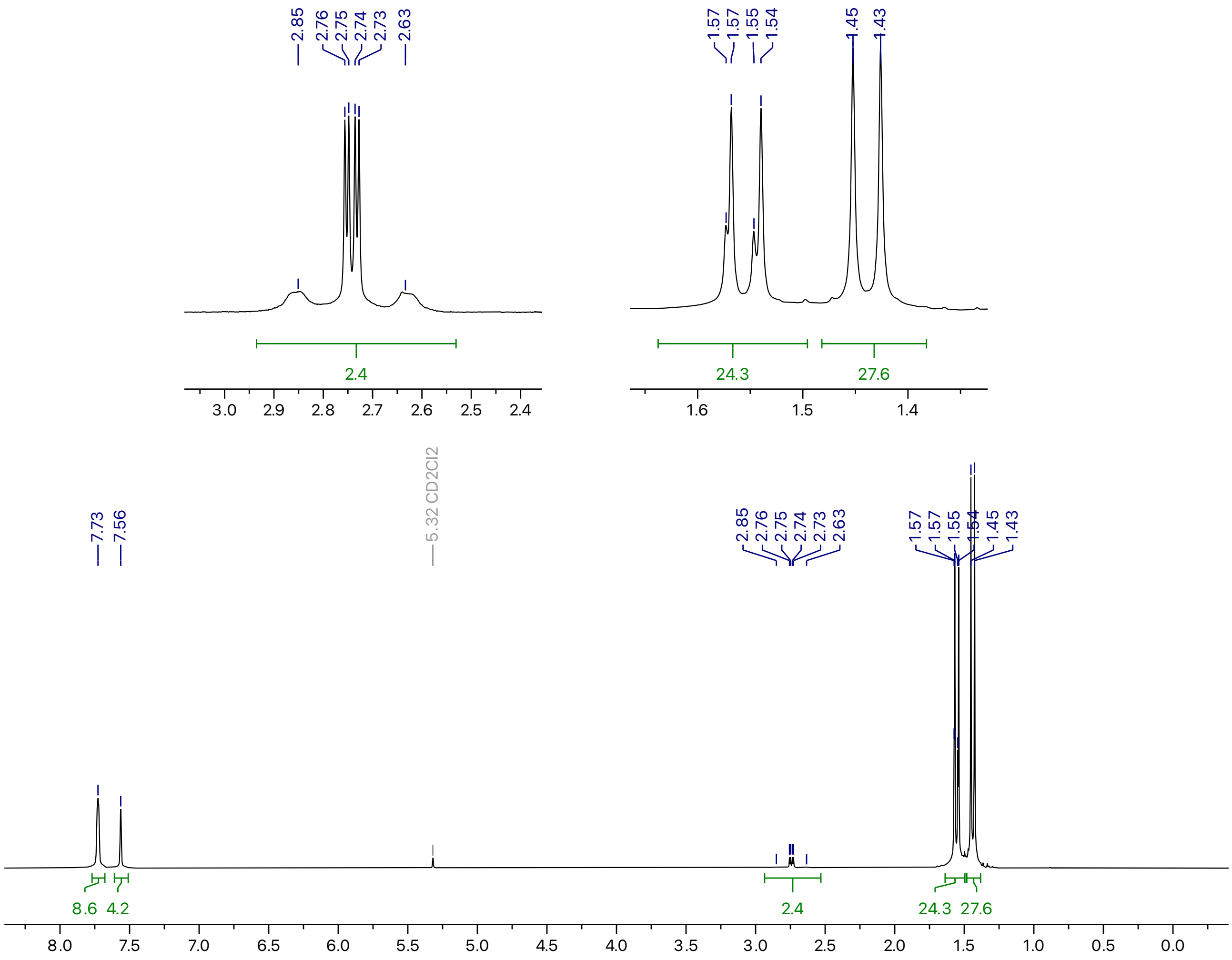

Figure S19. ^{1}H NMR spectrum of **5**[BAr^F$_4$] (500 MHz, CD$_2$Cl$_2$).

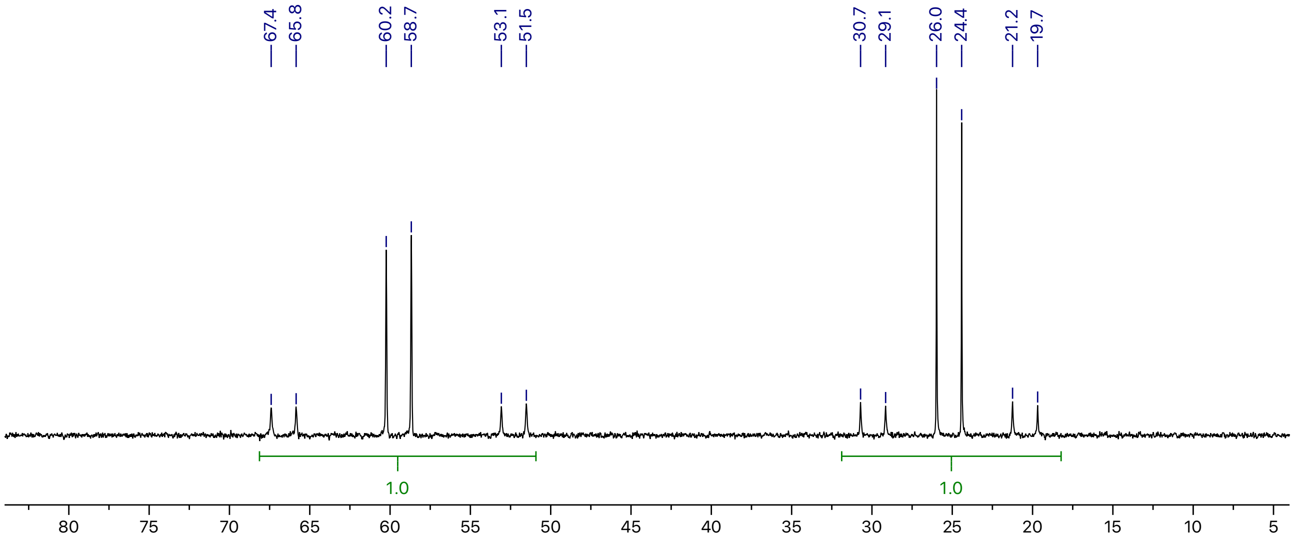

Figure S20. ^{31}P{^{1}H} NMR spectrum of **5**[BAr^F$_4$] (202 MHz, CD$_2$Cl$_2$).

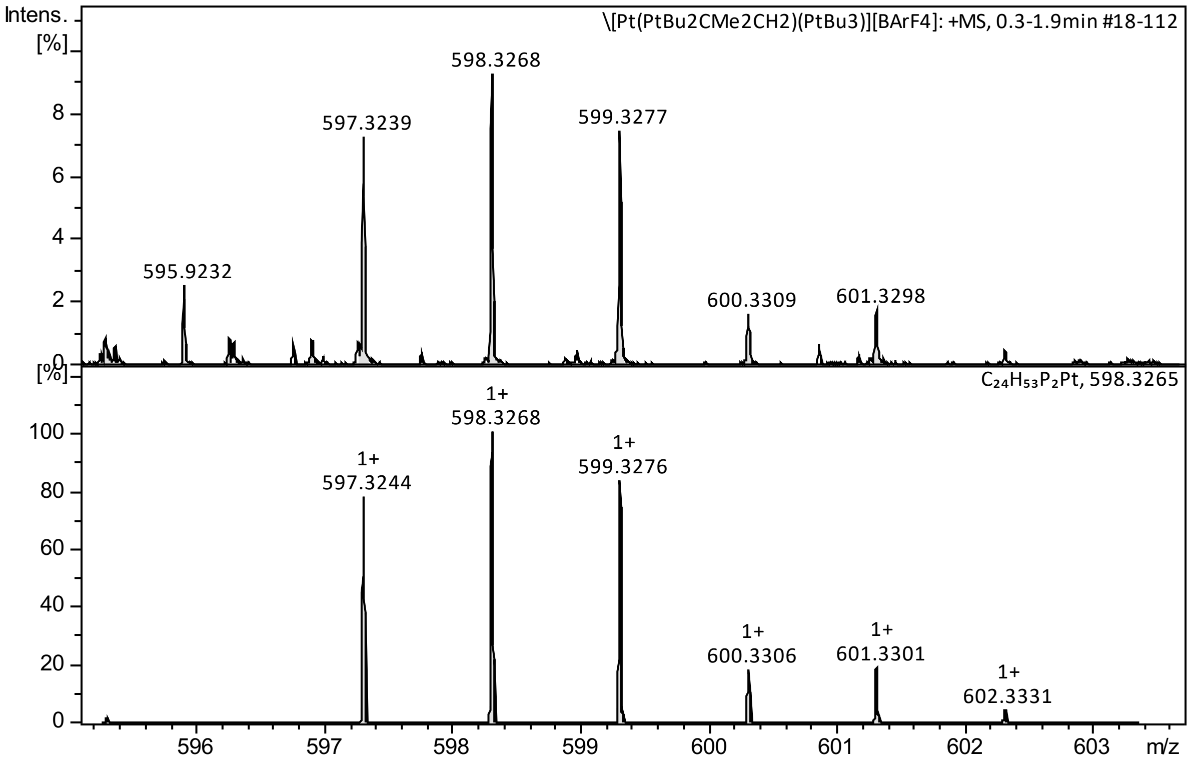

Figure S21. HR ESI-MS of **5**[BAr^F$_4$].

5 Characterisation of [Pt(P*t*Bu$_3$)$_2$H][BAr^F$_4$] (6[BAr^F$_4$])

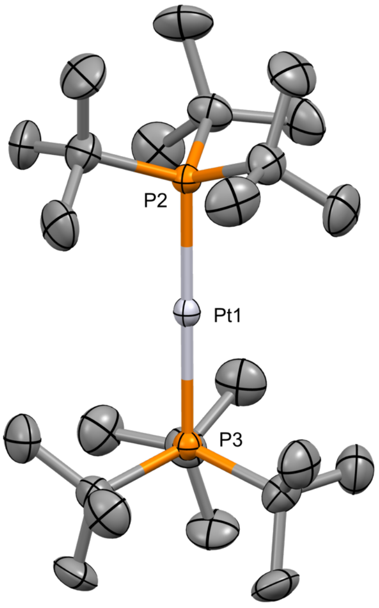

Figure S22. Solid-state structure of **6**[BAr^F$_4$]. The hydride ligand was not located from the Fourier difference map. Thermal ellipsoids drawn at 30% probably; minor disordered component (staggered substituents on P3, 45.6(11)%), hydrogen atoms, and anion omitted for clarity. Symmetry equivalent phosphine substituents are generated using the operations: ½+*z*, -½-*x*, -1-*y* and -½-*y*, -1-*z*, -½+*x*. Selected data: Pt1-P2, 2.314(3) Å; Pt1-P3, 2.320(3) Å; P2-Pt1-P3, 180°.

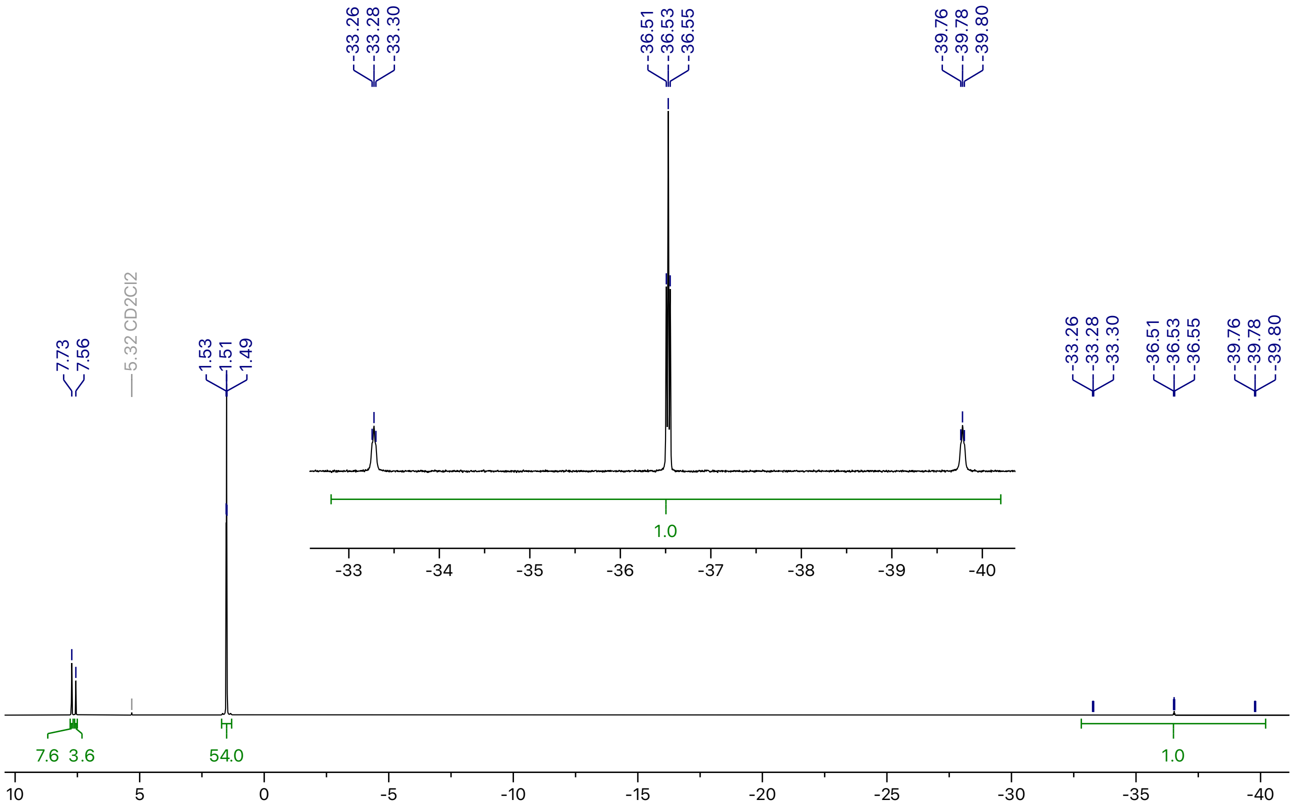

Figure S23. ^{1}H NMR spectrum of **6**[BAr^{F}_{4}] (400 MHz, CD_2Cl_2).

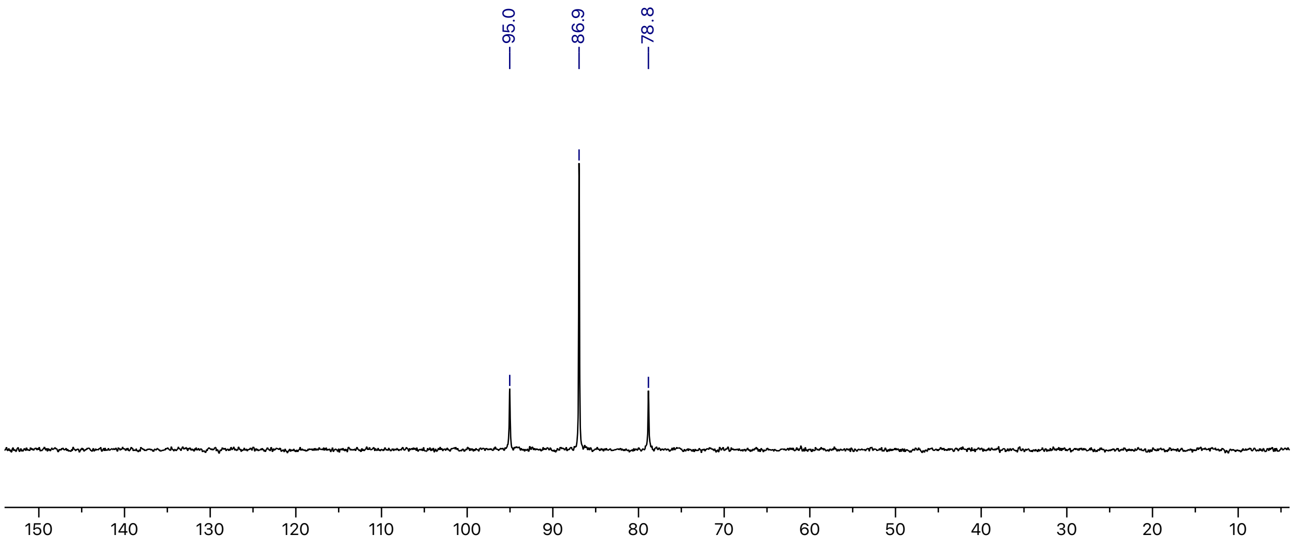

Figure S24. $^{31}P\{^{1}H\}$ NMR spectrum of **6**[BAr^{F}_{4}] (162 MHz, CD_2Cl_2).

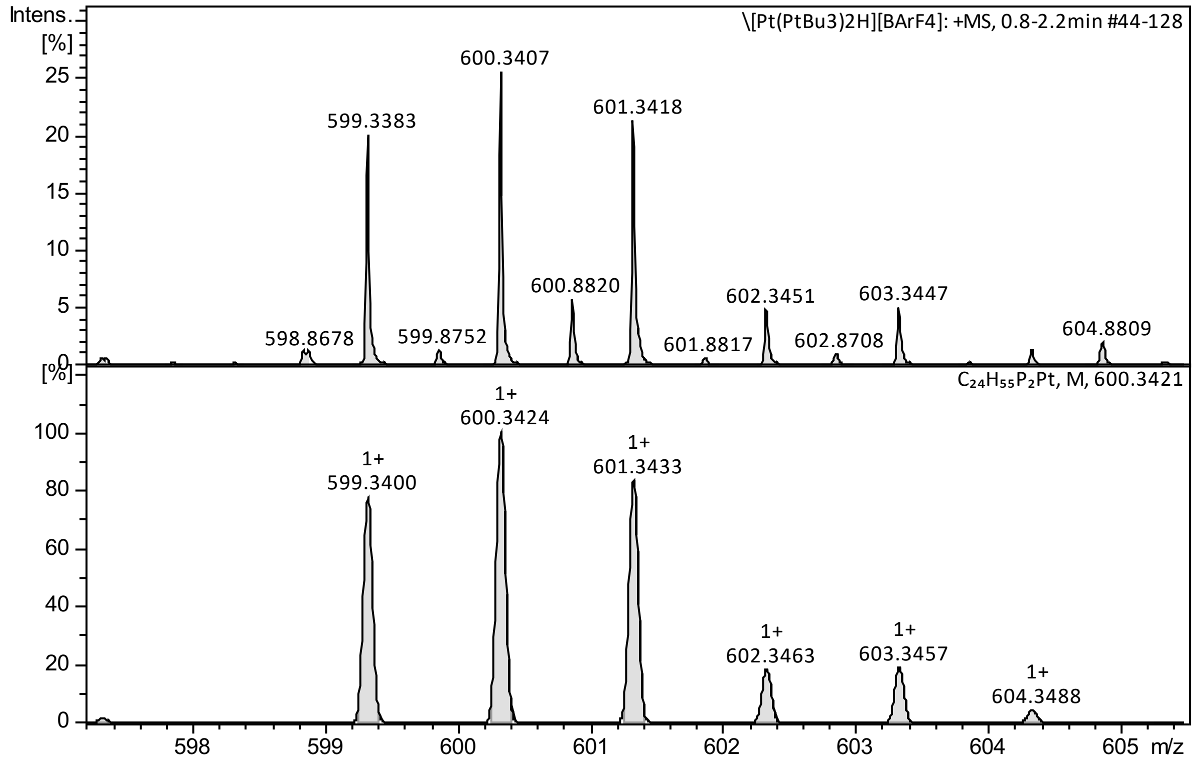

Figure S25. HR ESI-MS of **6**[BAr$^F_4$].

6 Reaction of 4[BAr$^F_4$] with $^•$OMes*

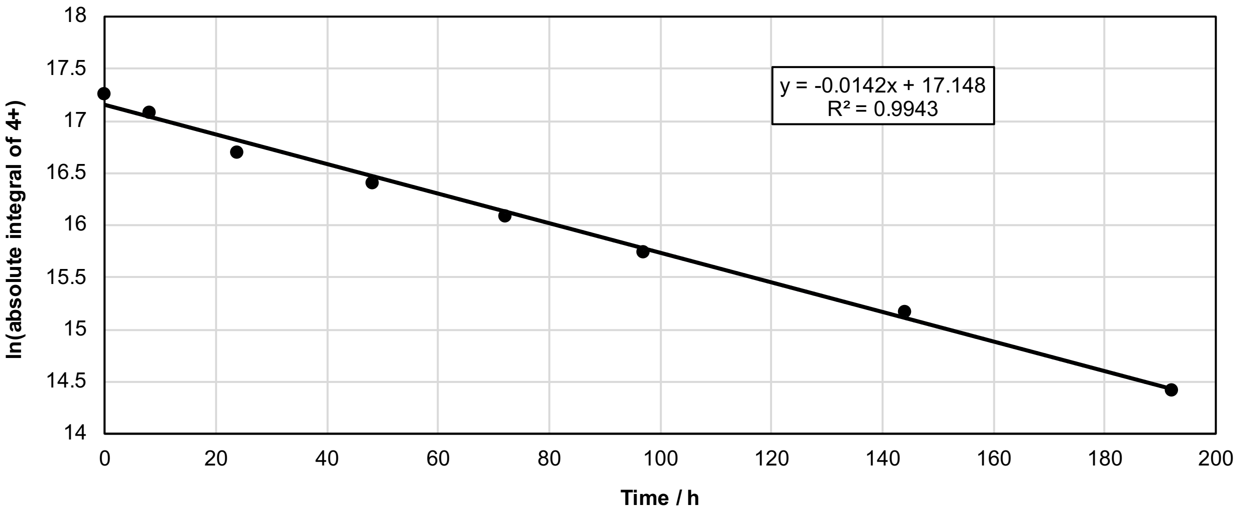

Figure S26. Kinetic analysis for the reaction of **4**[BAr$^F_4$] with 1.2 equivalents of $^•$OMes* in DFB.

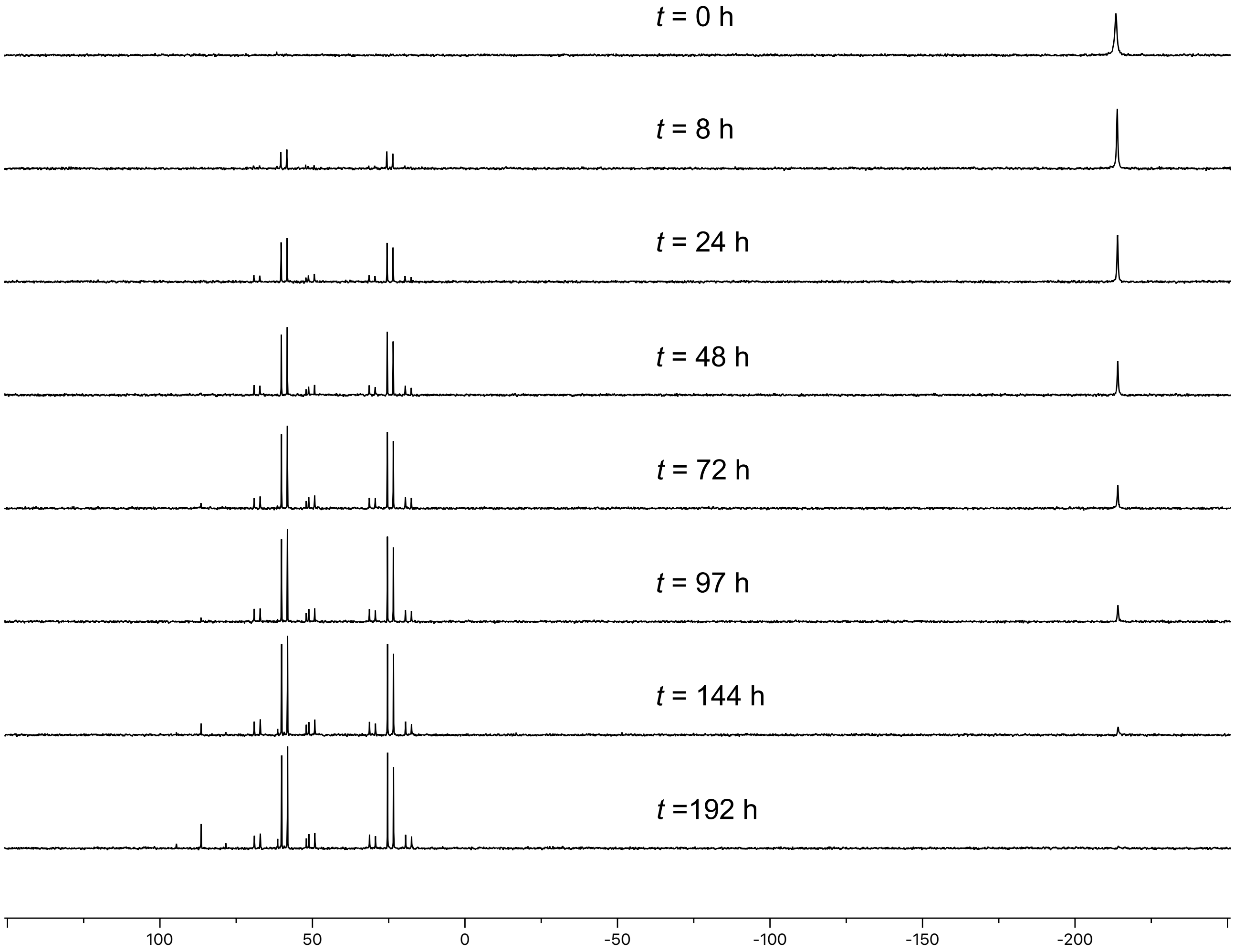

Figure S27. $^{31}P\{^{1}H\}$ NMR spectra collected during the reaction of **4**[BAr$^{F}_{4}$] with 1.2 equivalents of •OMes* in DFB (126 MHz).

7 Characterisation of [M(PAd$_3$)$_2$][BAr^F$_4$] (M = Pd, 13; Pt, 14)

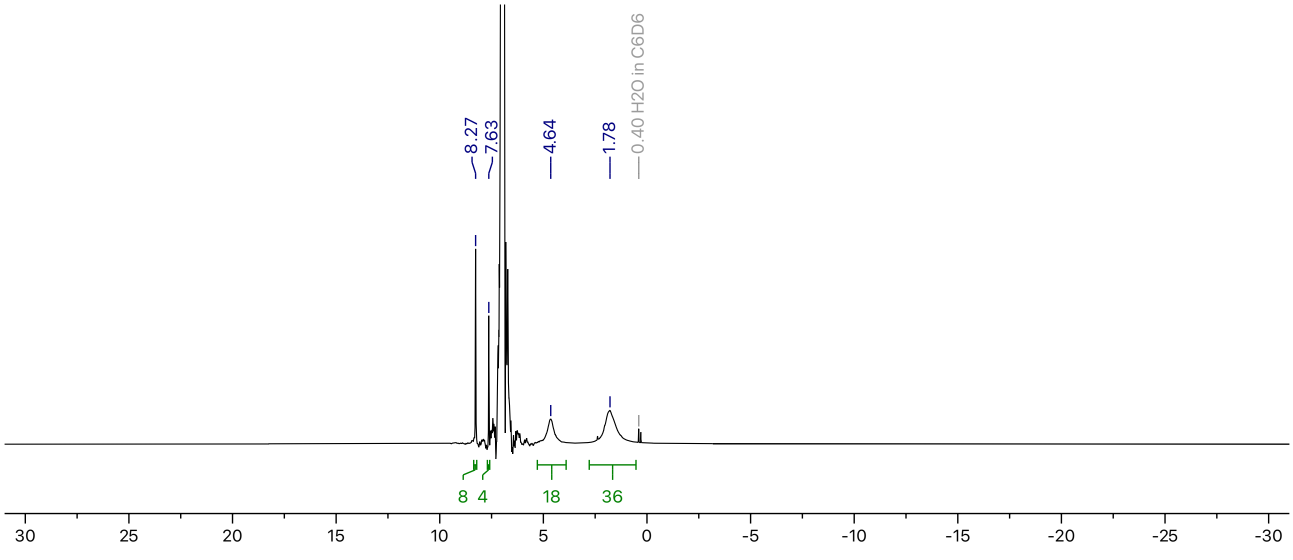

Figure S28. ^{1}H NMR spectrum of **13** in DFB (400 MHz).

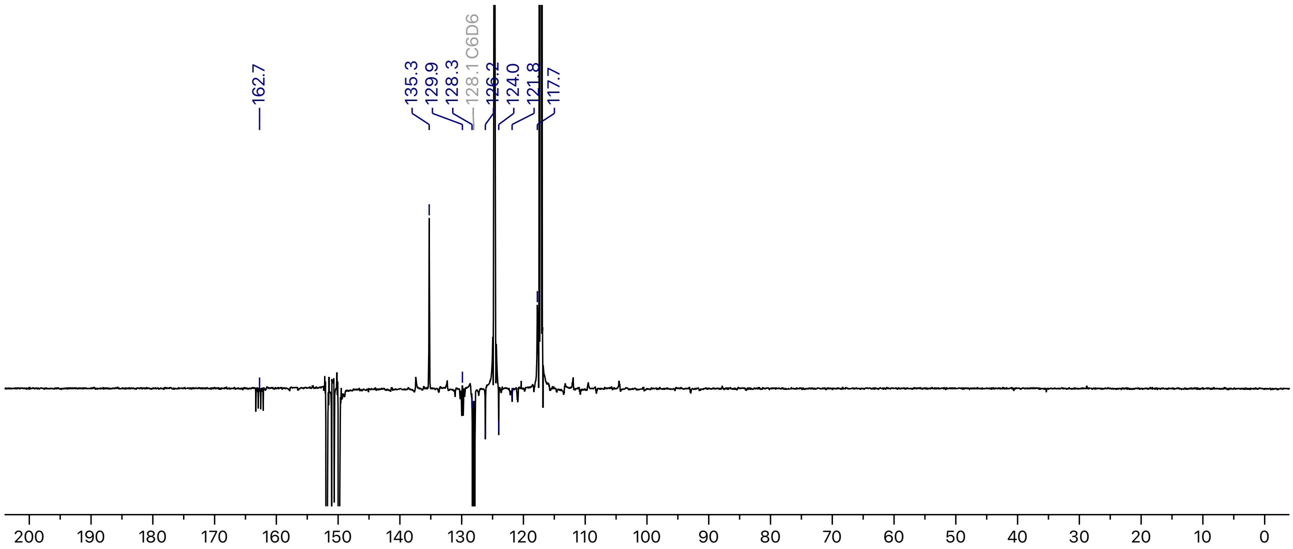

Figure S29. $^{13}C\{^{1}H\}$ APT NMR spectrum of **13** in DFB (126 MHz).

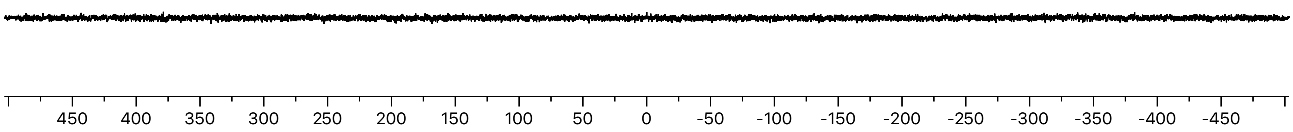

Figure S30. $^{31}P\{^{1}H\}$ NMR spectrum of **13** in DFB (162 MHz).

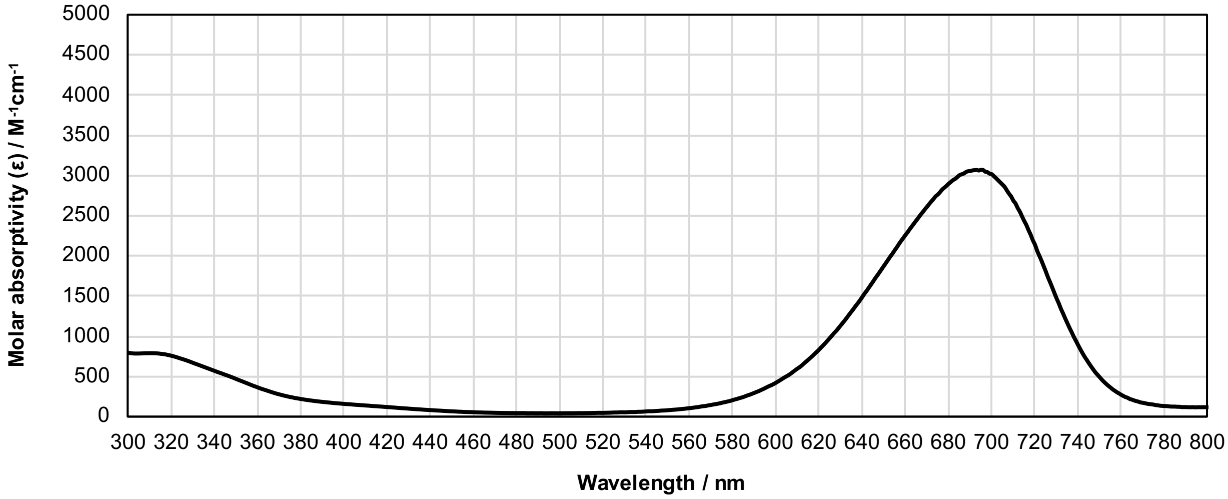

Figure S31. UV-vis spectrum of **13** in DFB.

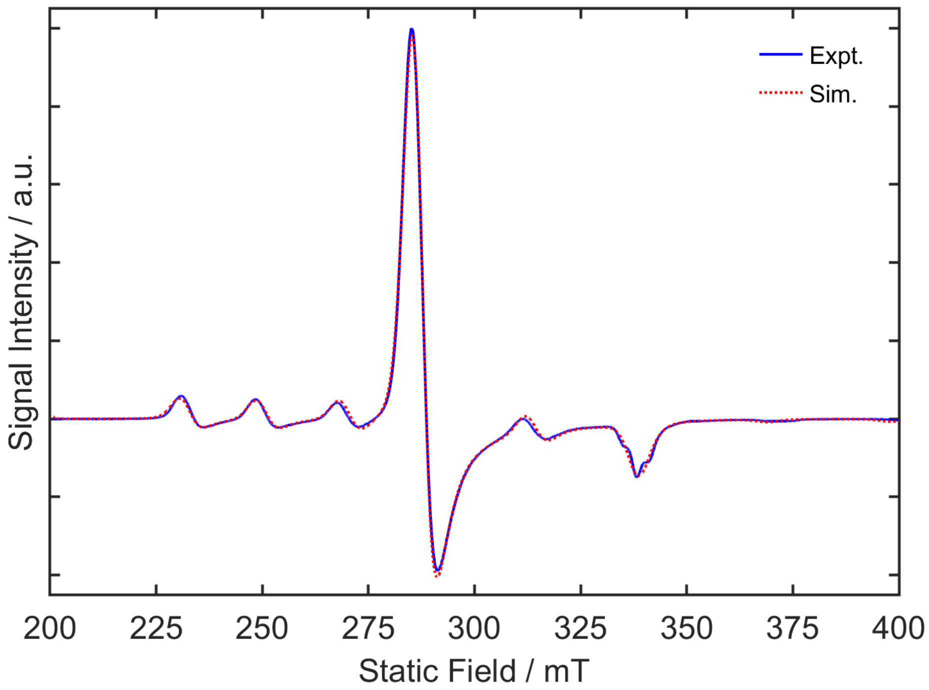

Figure S32. EPR spectrum of **13** (DFB glass, 200 K, a.u. = arbitrary units) after baseline subtraction. Fitting gave $g_{\perp}$ = 2.333, $g_{\parallel}$ = 1.979 and A_{iso} = 24.6 mT, using phenomenological line broadenings of 5.24 mT (Gaussian) and 1.38 mT (Lorentzian).

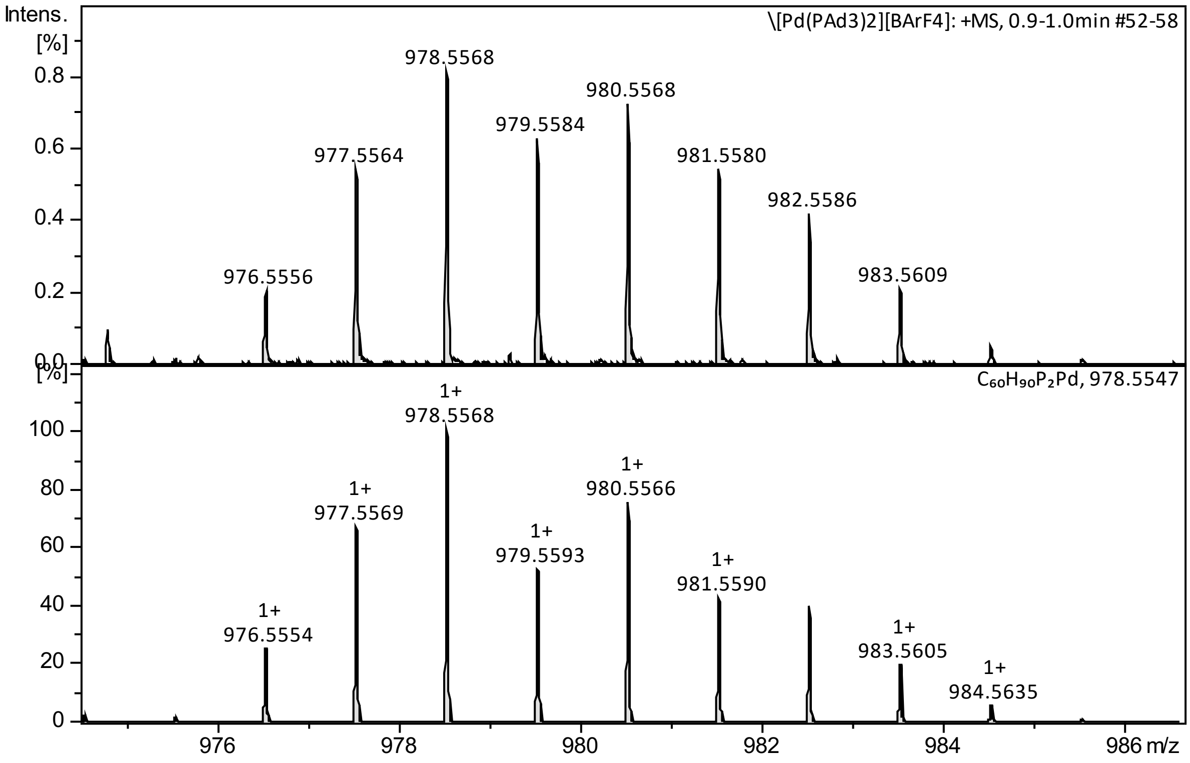

Figure S33. HR ESI-MS of **13**.

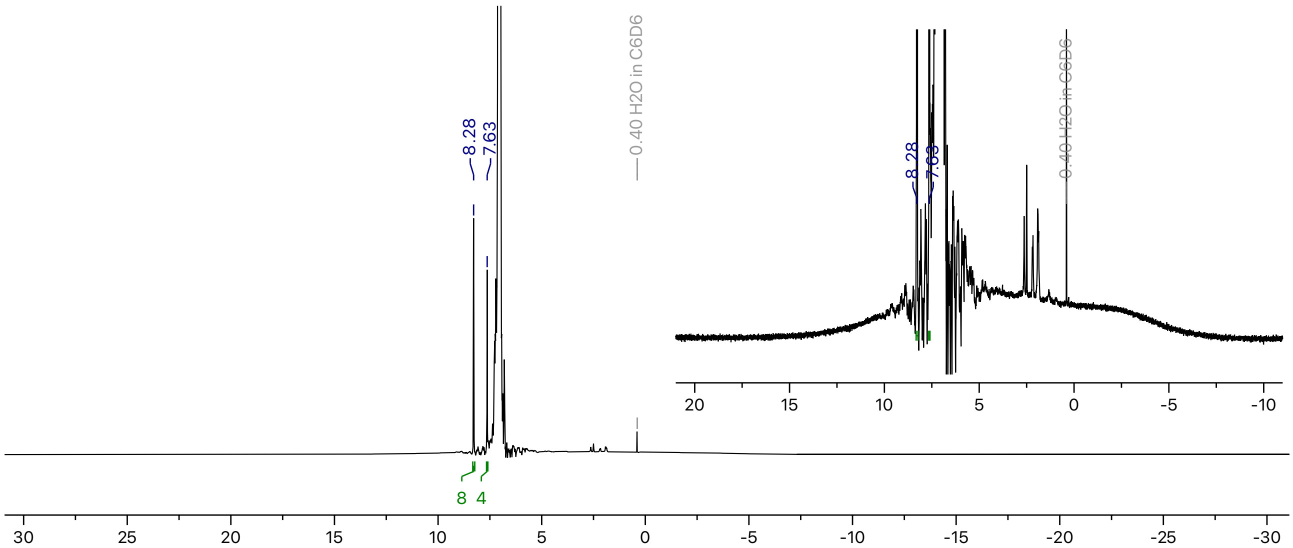

Figure S34. ^{1}H NMR spectrum of **14** in DFB (400 MHz).

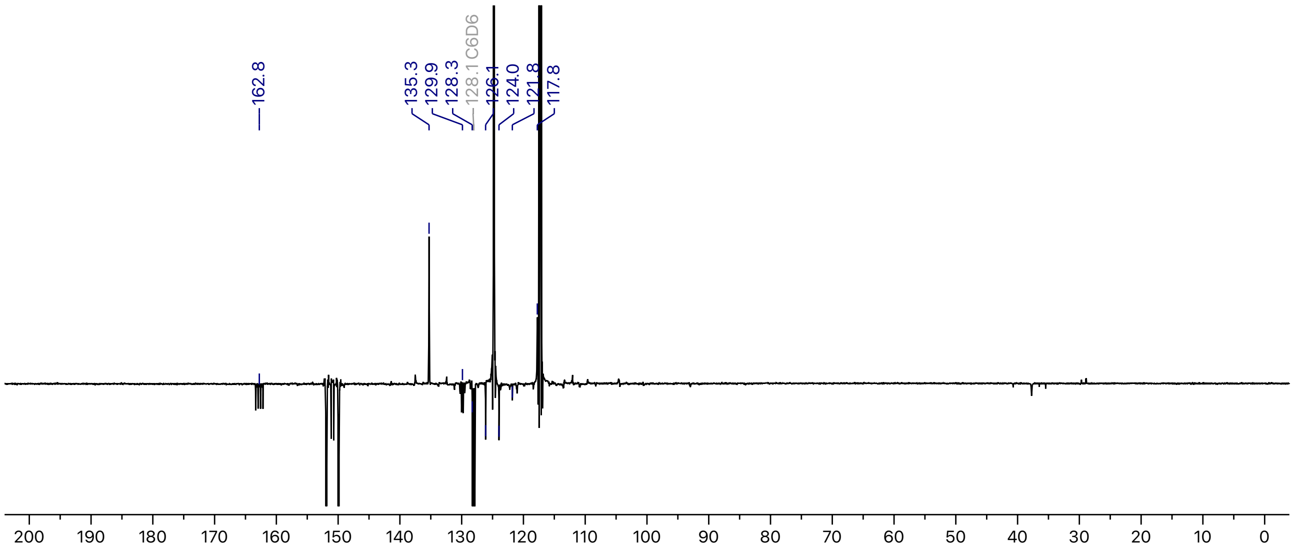

Figure S35. ^{13}C{^{1}H} APT NMR spectrum of **14** in DFB (126 MHz).

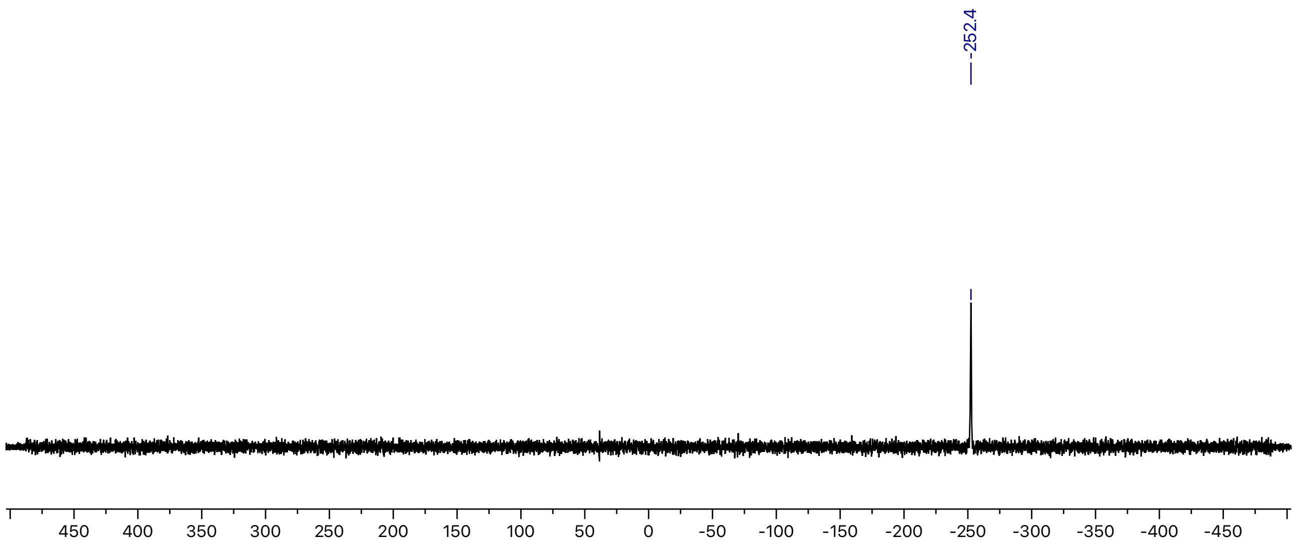

Figure S36. ^{31}P{^{1}H} NMR spectrum of **14** in DFB (162 MHz).

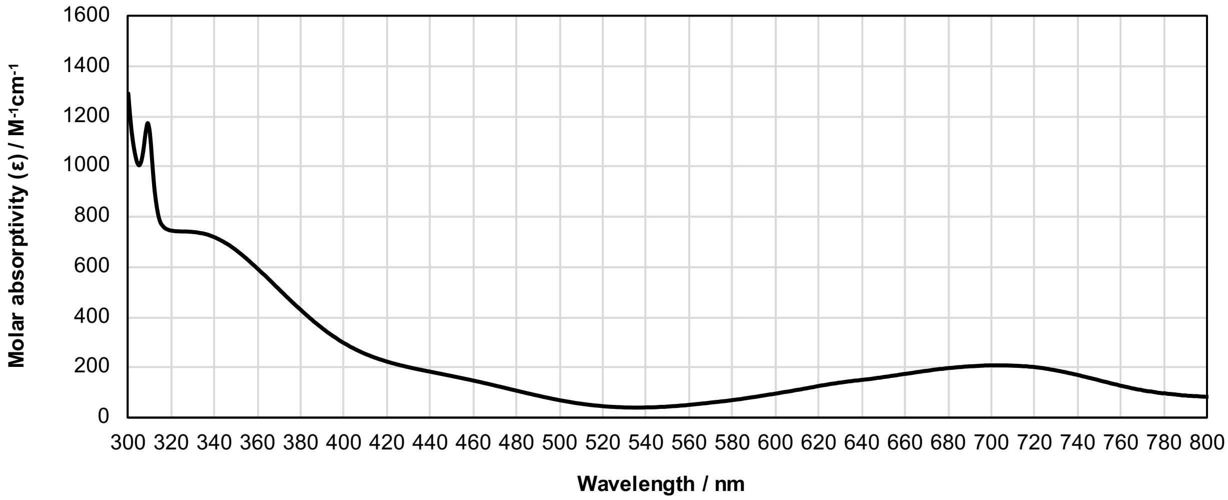

Figure S37. UV-vis spectrum of **14** in DFB.

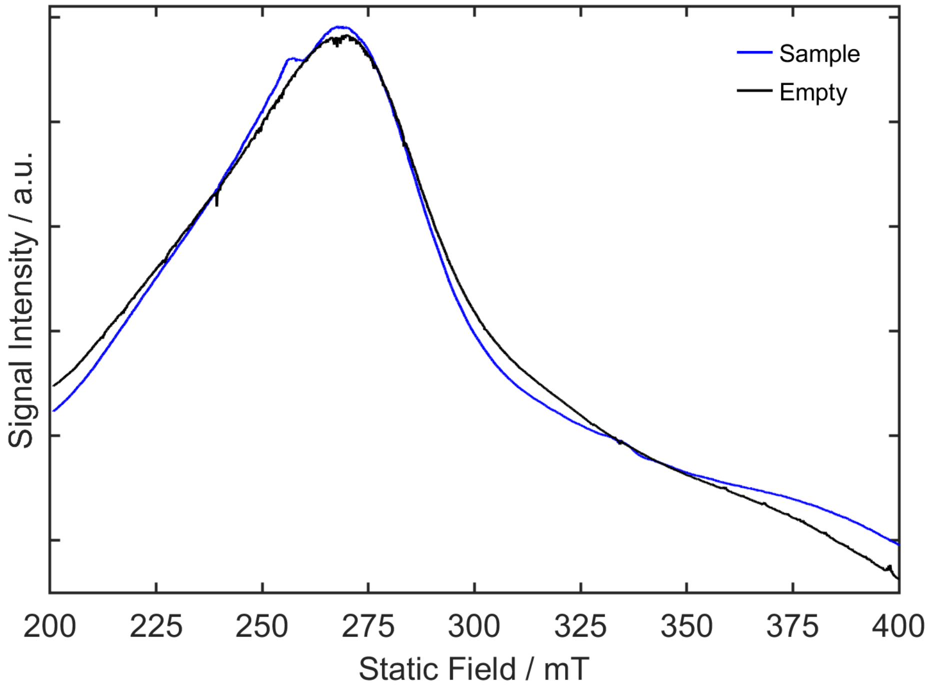

Figure S38. EPR spectrum of **14** (DFB glass, 100 K, a.u. = arbitrary units). The spectrum of an empty sample tube recorded under identical conditions is shown for comparison, indicating the apparent signal arises only from the cavity background.

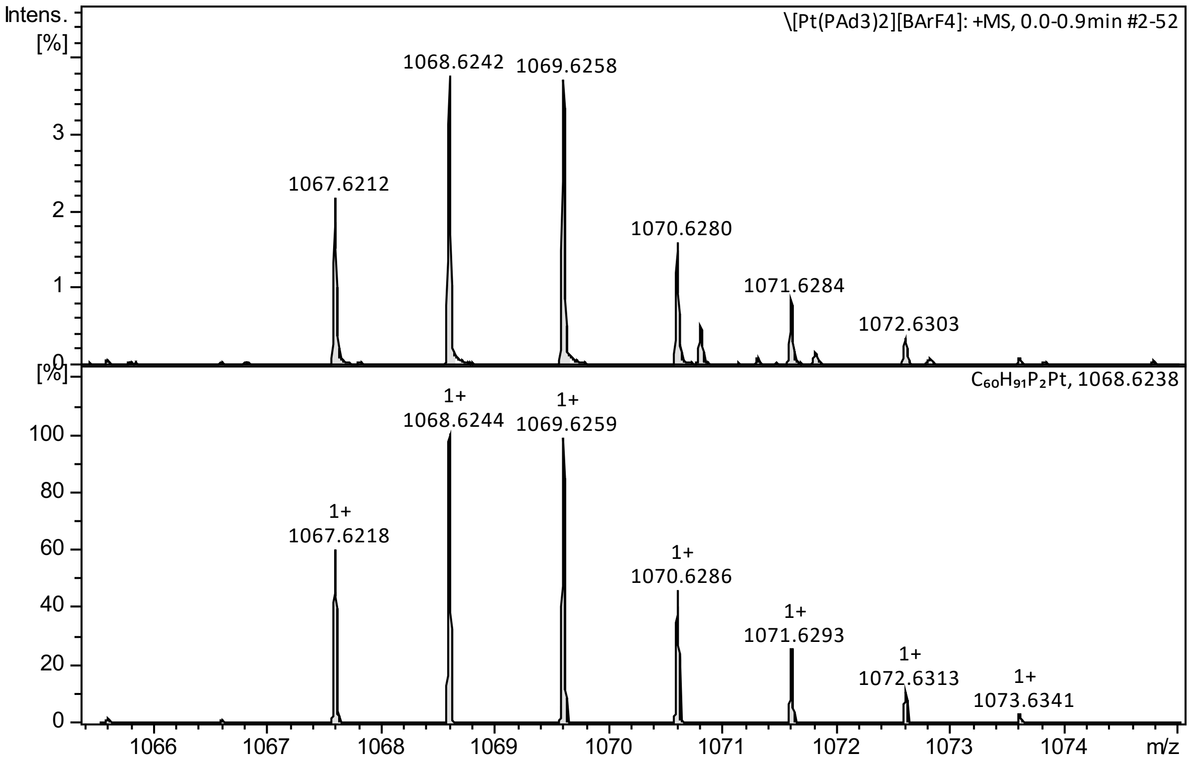

Figure S39. HR ESI-MS of **14**.

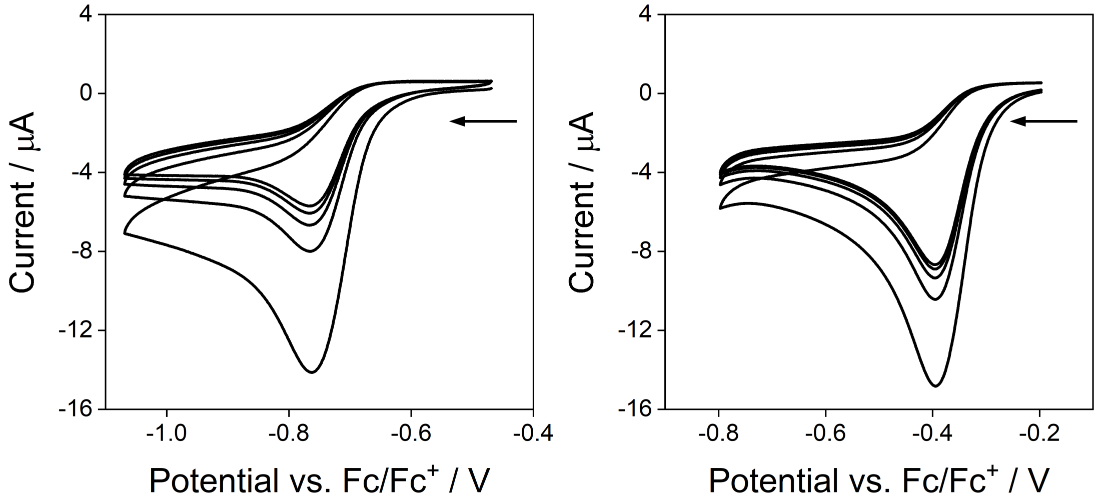

Figure S40. Potential cycling for the reduction of **13** (left) and **14** (right) in DFB (2 mM complex; 0.2 M [nBu$_4$N][BAr$^F_4$] electrolyte; glassy carbon working electrode, coiled Pt wire counter electrode and Ag wire quasi-reference electrode; scan rate = 100 mV·s^{-1}). $E_{P/2}$ = -0.70 V and -0.33 V, respectively. Reduction of the peak current is attributed to fowling of the electrode by precipitation of [M(PAd$_3$)$_2$] (M = Pd, **11**; Pt, **12**) onto the electrode surface.

8 Solution stability of [M(PAd$_3$)$_2$][BAr$^F{}_4$] (M = Pd, 13; Pt, 14)

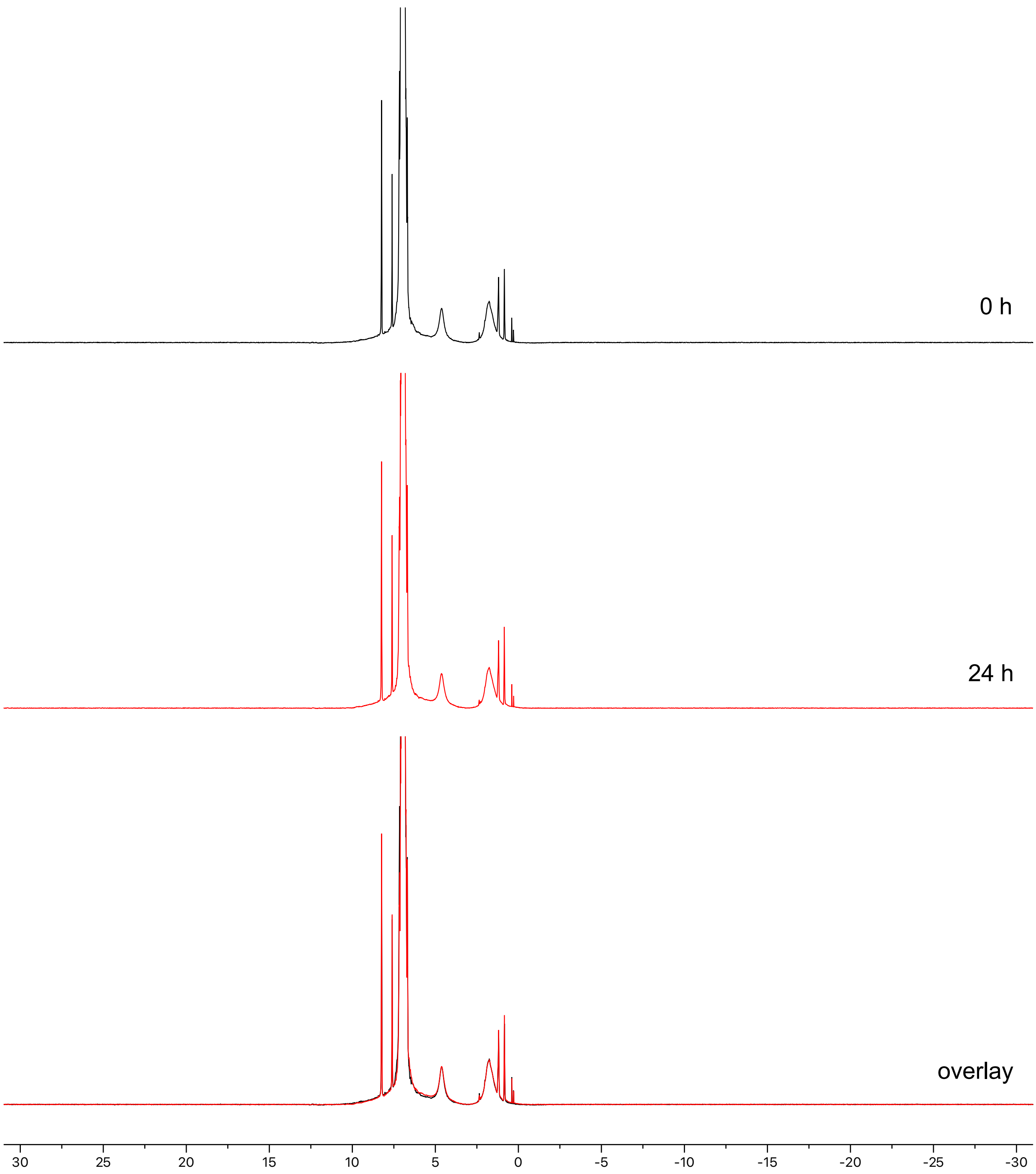

Figure S41. ^{1}H NMR spectra of **13** collected over time in DFB (400 MHz).

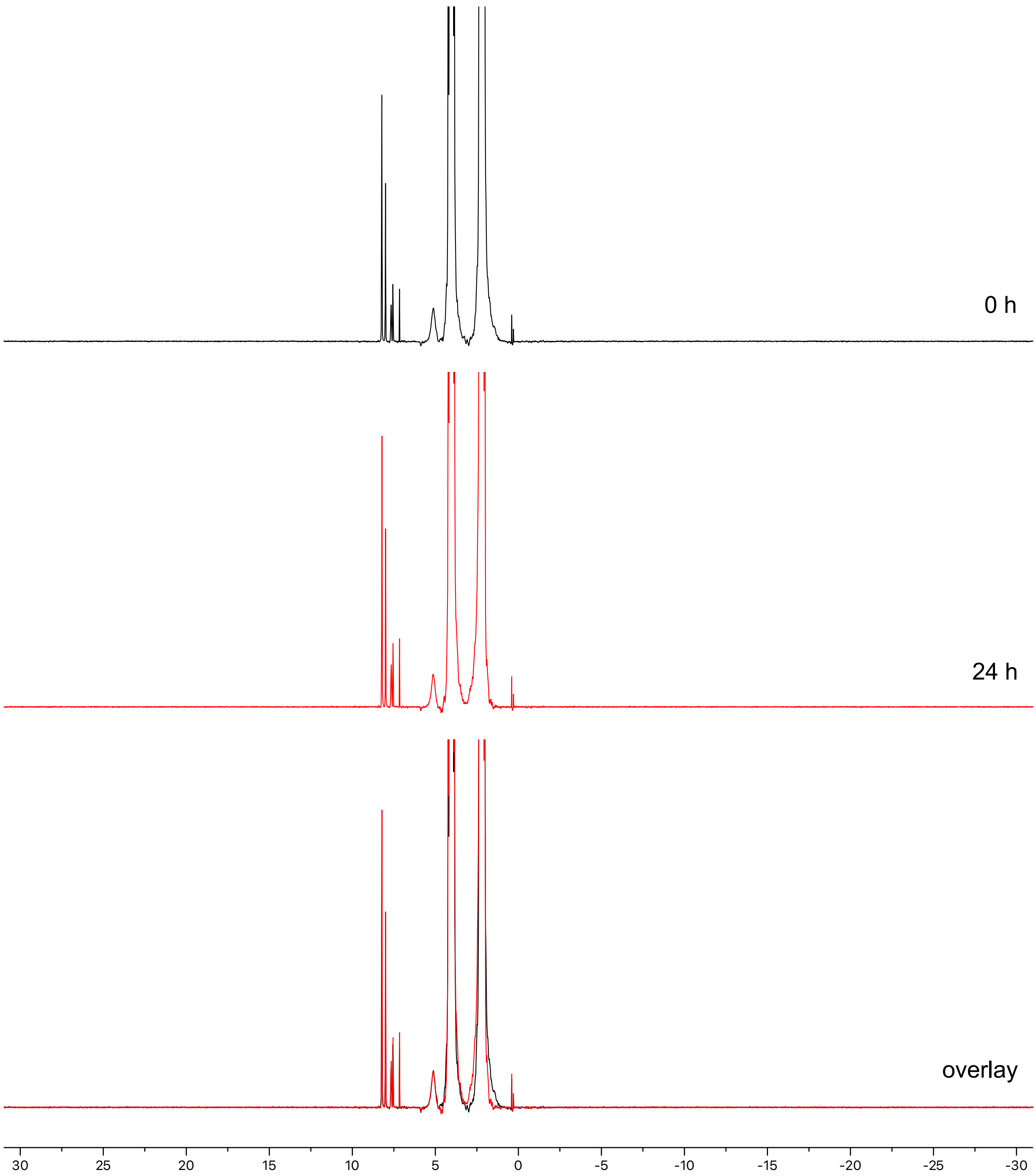

Figure S42. ^{1}H NMR spectra of **13** collected over time in THF (400 MHz).

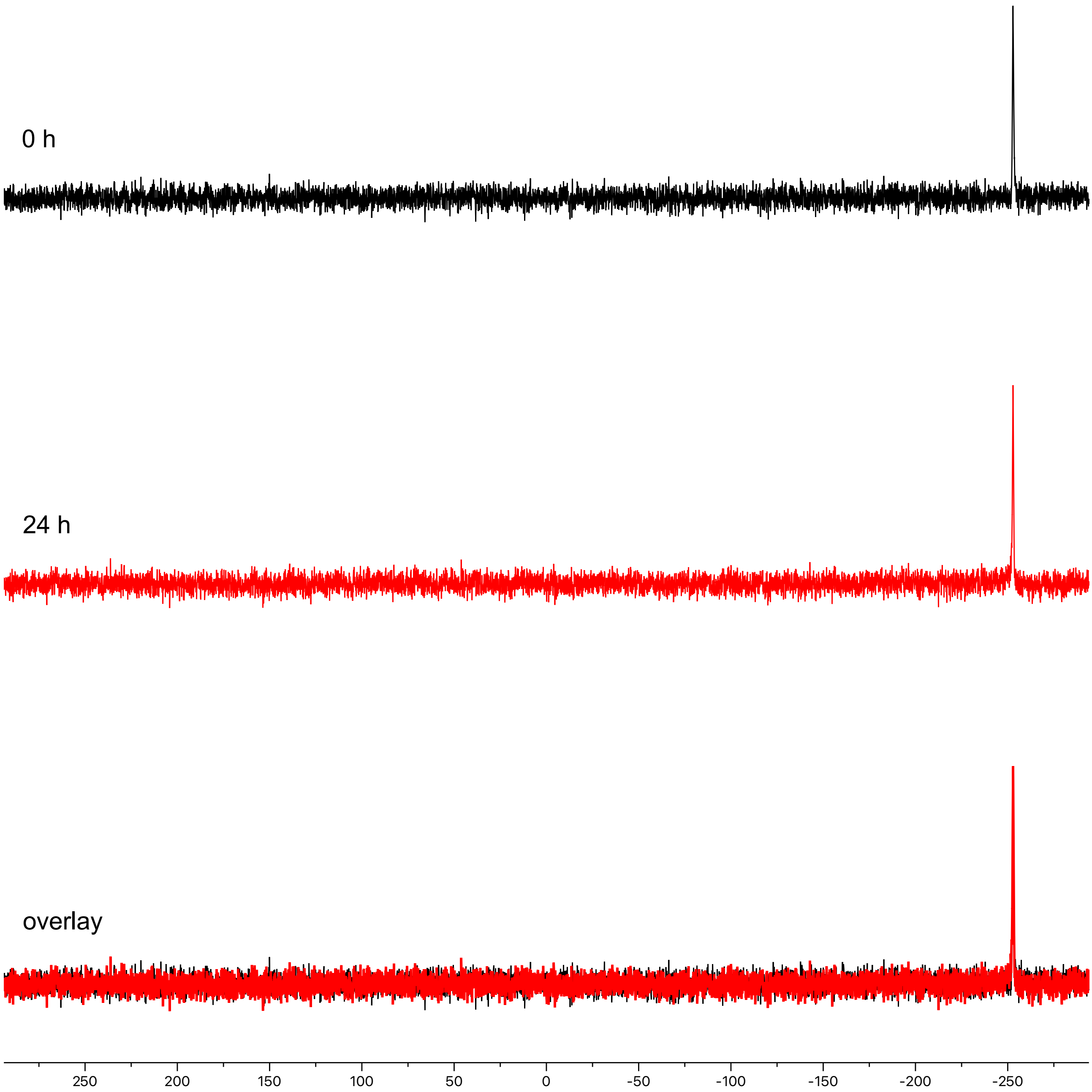

Figure S43. $^{31}P\{^{1}H\}$ NMR spectra of **14** collected over time in DFB (162 MHz).

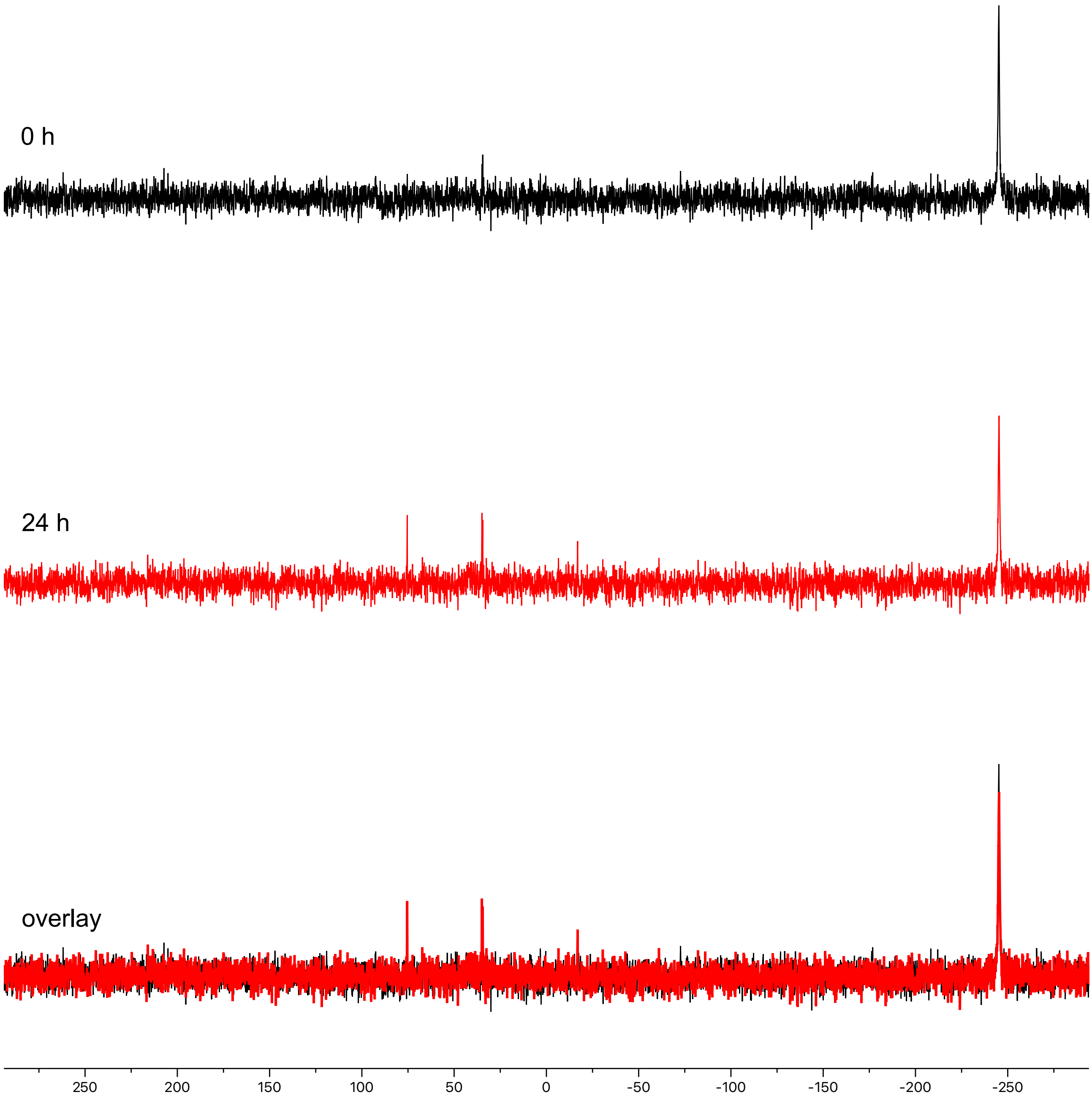

Figure S44. $^{31}P\{^{1}H\}$ NMR spectra of **14** collected over time in THF (162 MHz).

9 Reactions of 3[BAr^F$_4$], 4[BAr^F$_4$], 13 and 14 with 9,10-dihydroanthracene

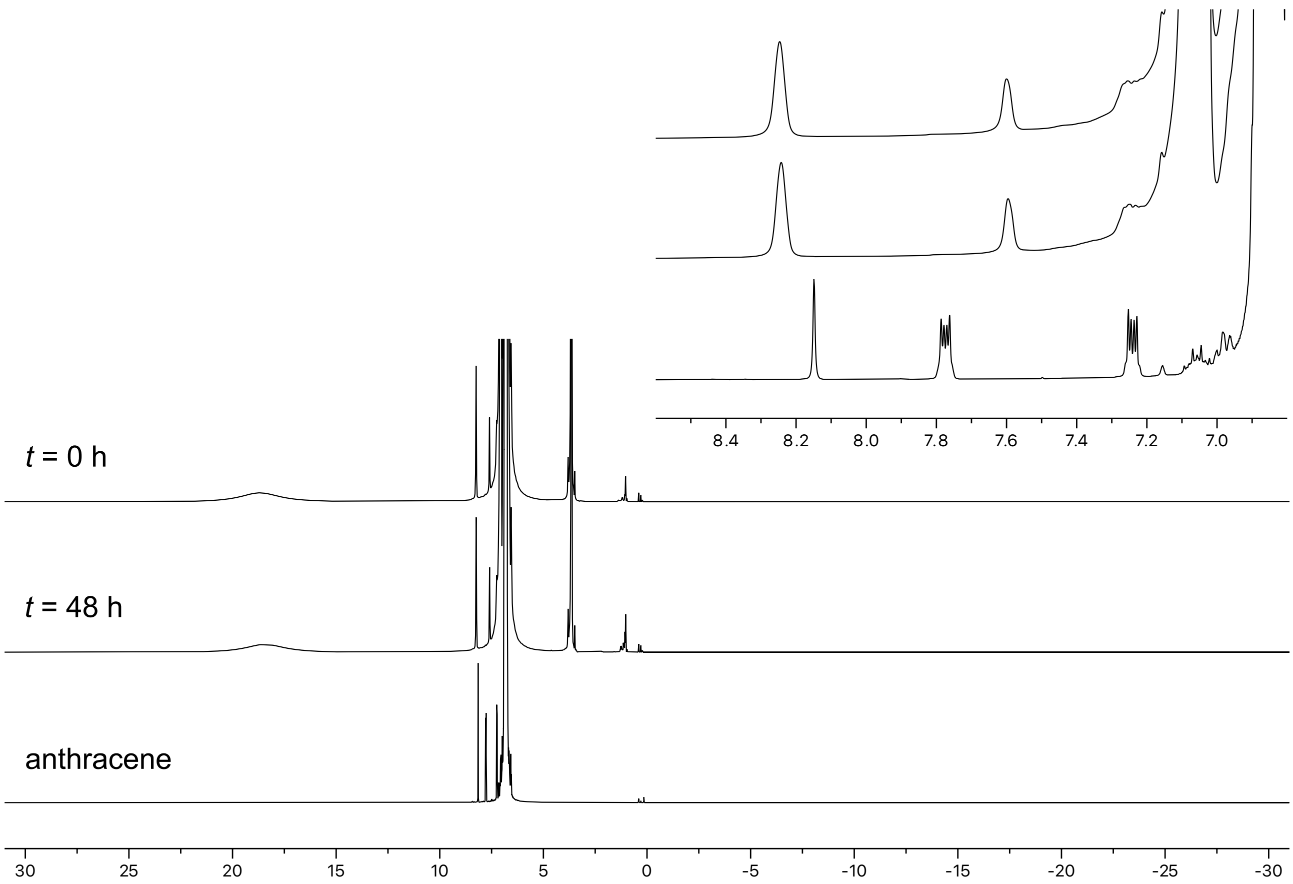

Figure S45. ^{1}H NMR spectra collected during the attempted reaction of **3**[BAr^F$_4$] with 50 equivalents of 9,10-dihydroanthracene in DFB and anthracene in DFB (400 MHz).

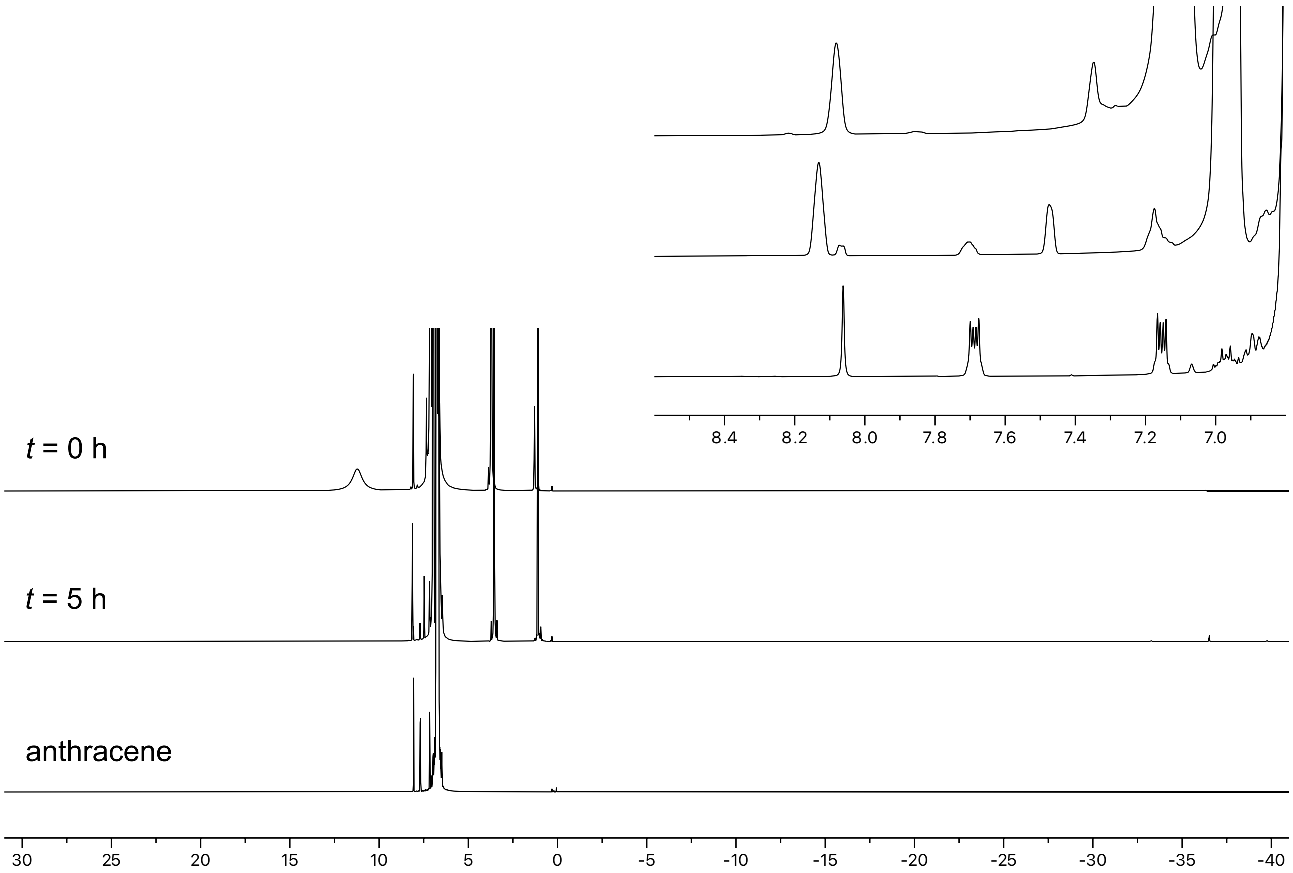

Figure S46. ^{1}H NMR spectra collected during the reaction of **4**[BAr$^{F}{}_{4}$] with 50 equivalents of 9,10-dihydroanthracene in DFB and anthracene in DFB (400 MHz).

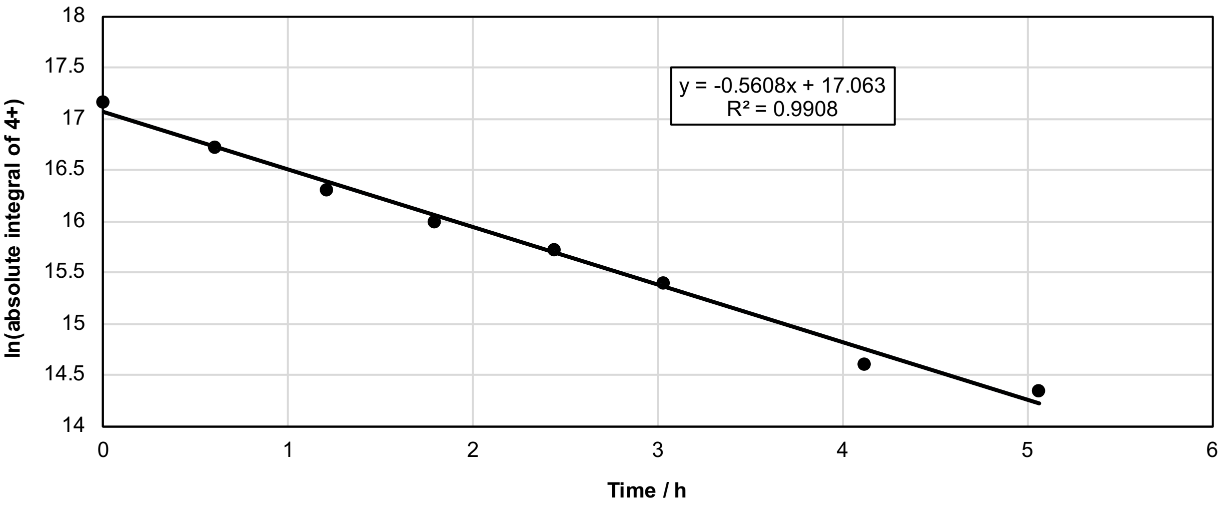

Figure S47. Kinetic analysis of the reaction of **4**[BAr$^{F}{}_{4}$] with 50 equivalents of 9,10-dihydroanthracene in DFB.

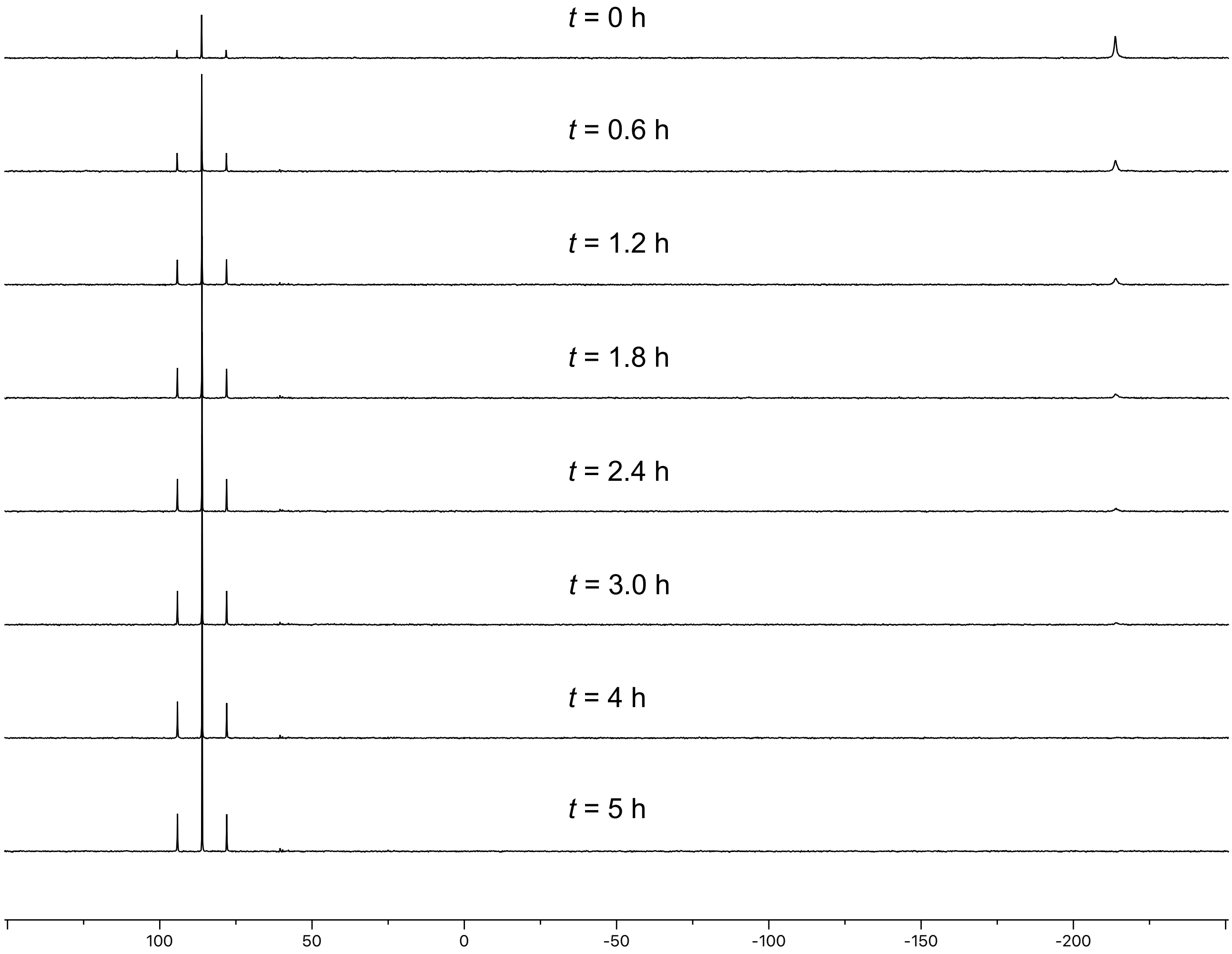

Figure S48. $^{31}P\{^{1}H\}$ NMR spectra collected during the reaction of **4**[BAr$^{F}_{4}$] with 50 equivalents of 9,10-dihydroanthracene in DFB (126 MHz).

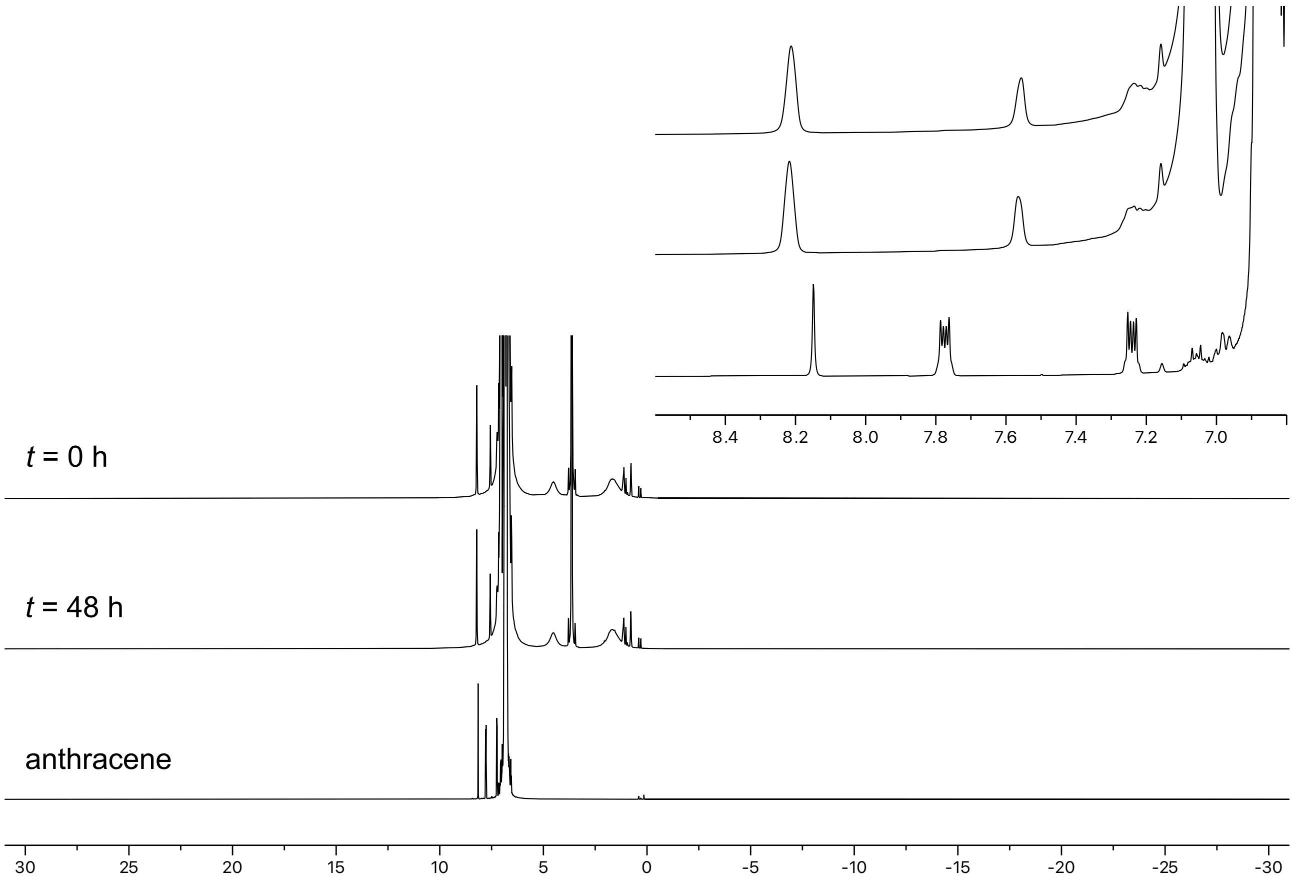

Figure S49. ^{1}H NMR spectra collected during the attempted reaction of **13** with 50 equivalents of 9,10-dihydroanthracene in DFB and anthracene in DFB (400 MHz).

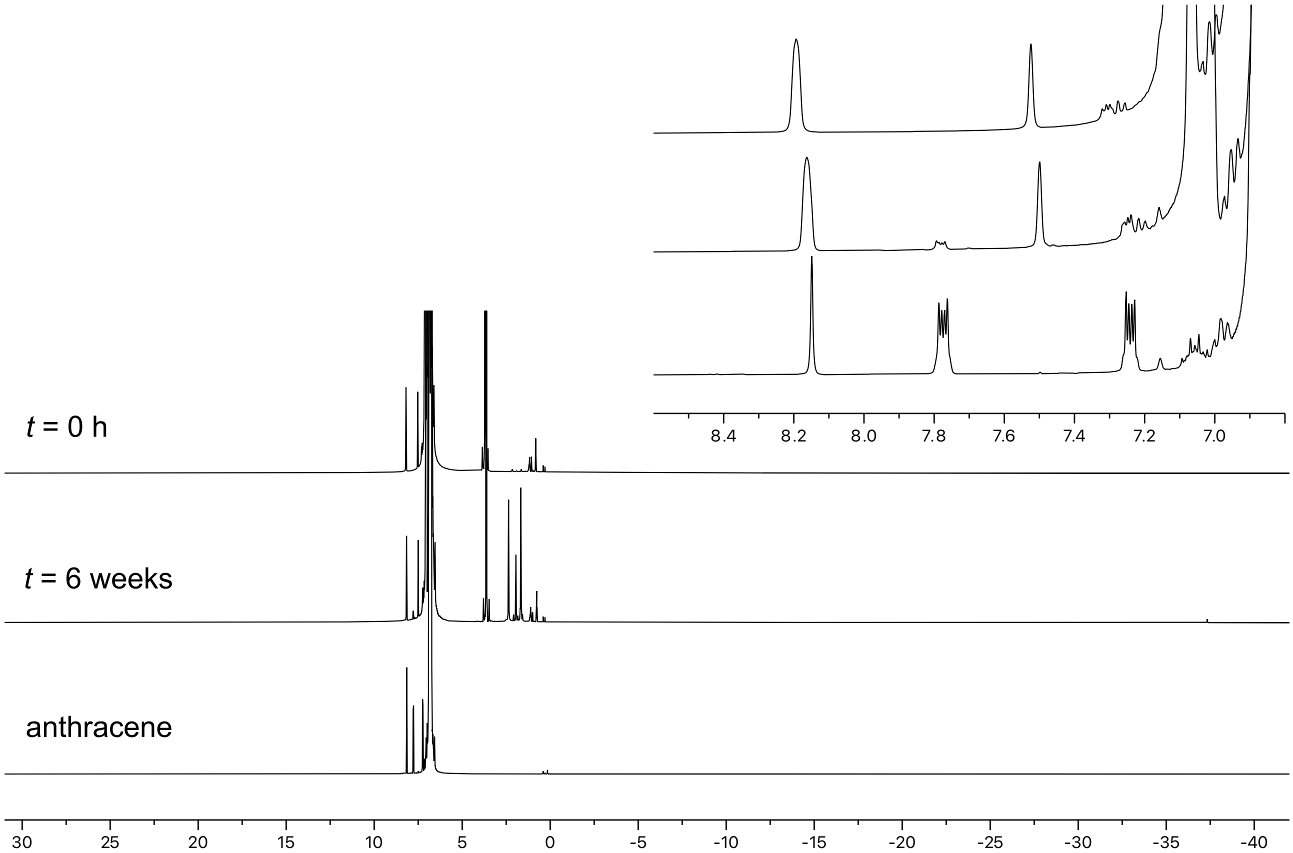

Figure S50. ^{1}H NMR spectra collected during the reaction of **14** with 50 equivalents of 9,10-dihydroanthracene in DFB and anthracene in DFB (400 MHz).

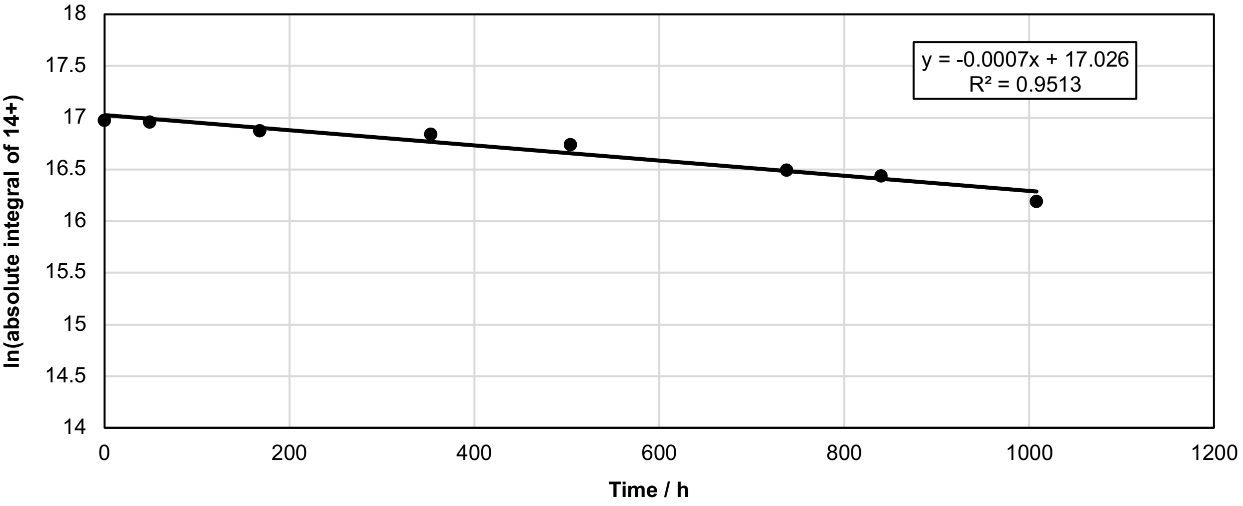

Figure S51. Kinetic analysis of the reaction of **14** with 50 equivalents of 9,10-dihydroanthracene in DFB.

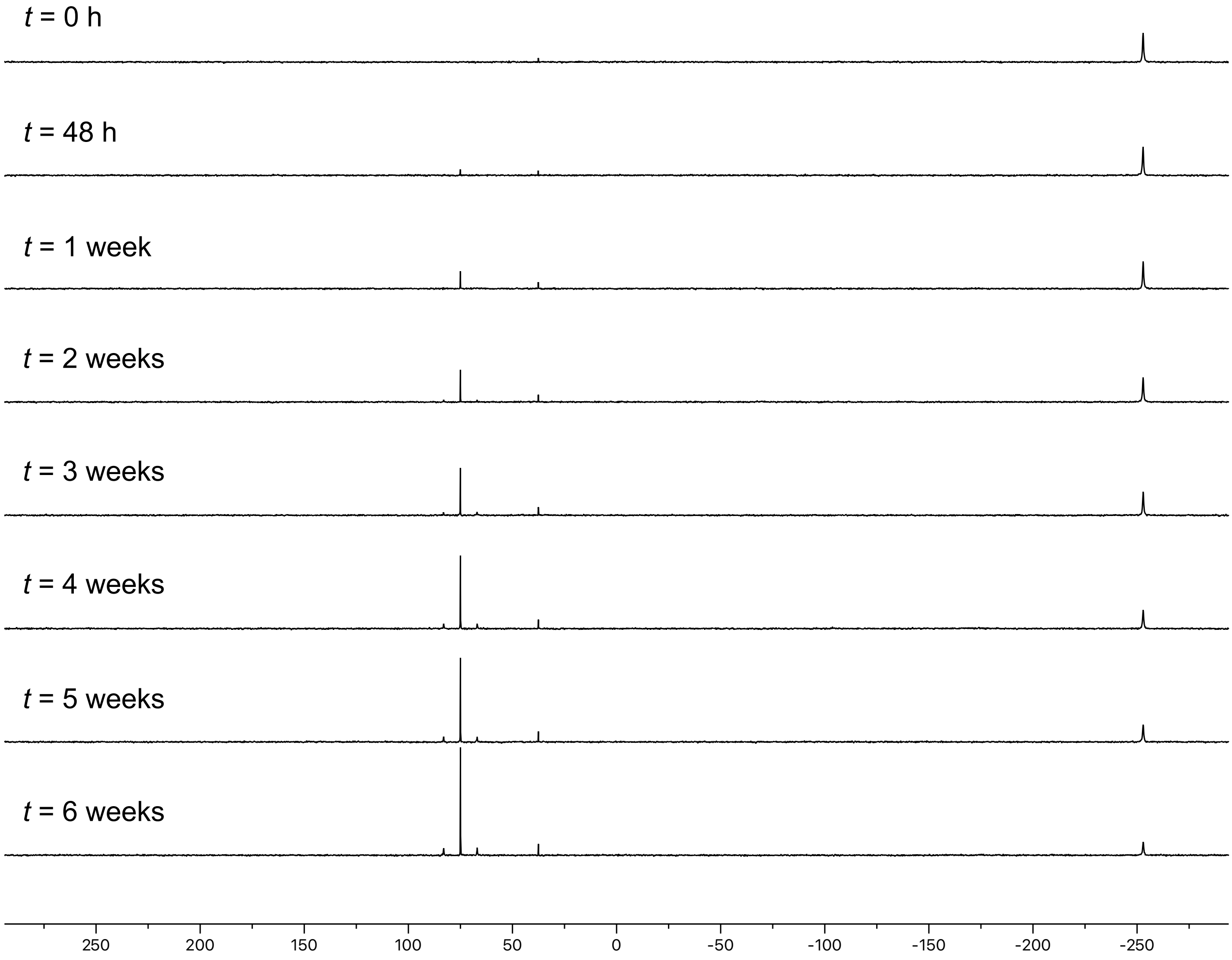

Figure S52. $^{31}P\{^1H\}$ NMR spectra collected during the reaction of **14** with 50 equivalents of 9,10-dihydroanthracene in DFB (126 MHz).

10 Characterisation of [Pd(P*t*Bu$_3$)$_2$H][BAr$^F_4$] (15)

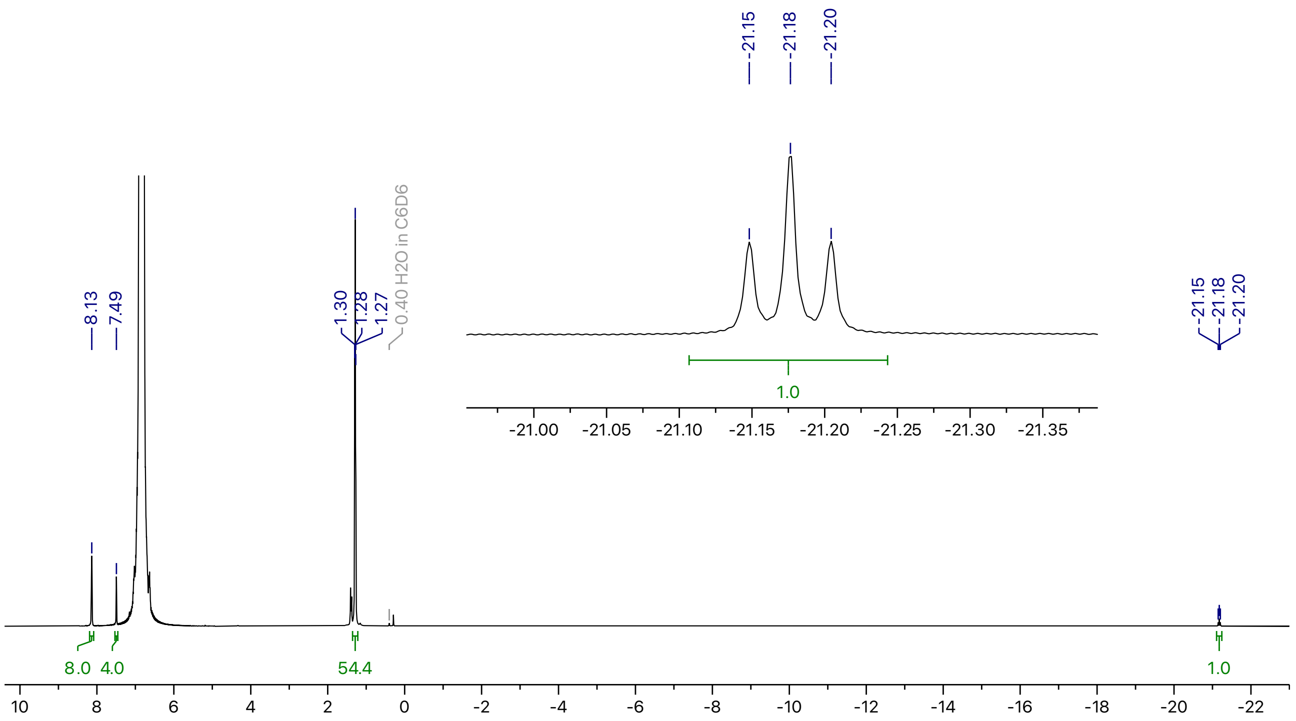

Figure S53. ^{1}H NMR spectrum of **15** (500 MHz, DFB).

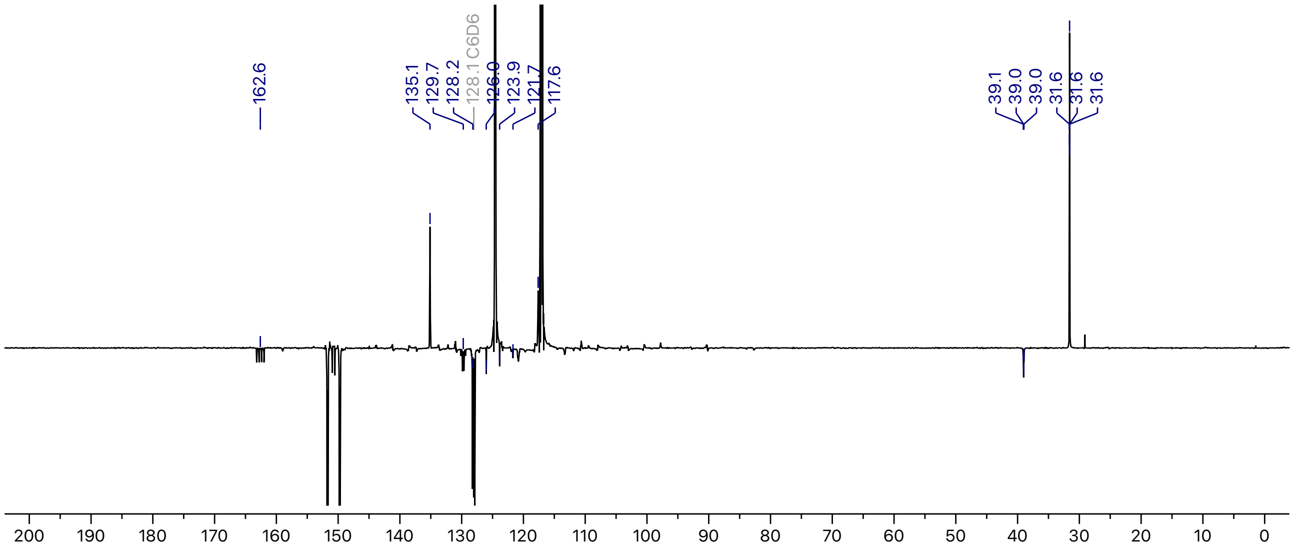

Figure S54. ^{13}C{^{1}H} APT NMR spectrum of **15** (126 MHz, DFB).

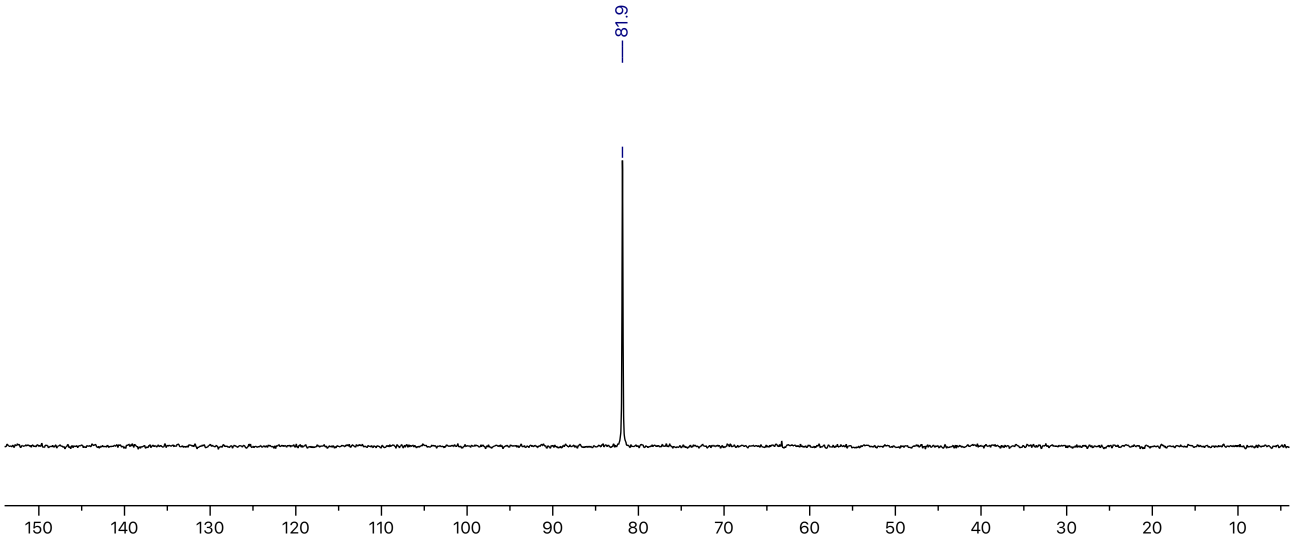

Figure S55. $^{31}P\{^{1}H\}$ NMR spectrum of **15** (162 MHz, DFB).

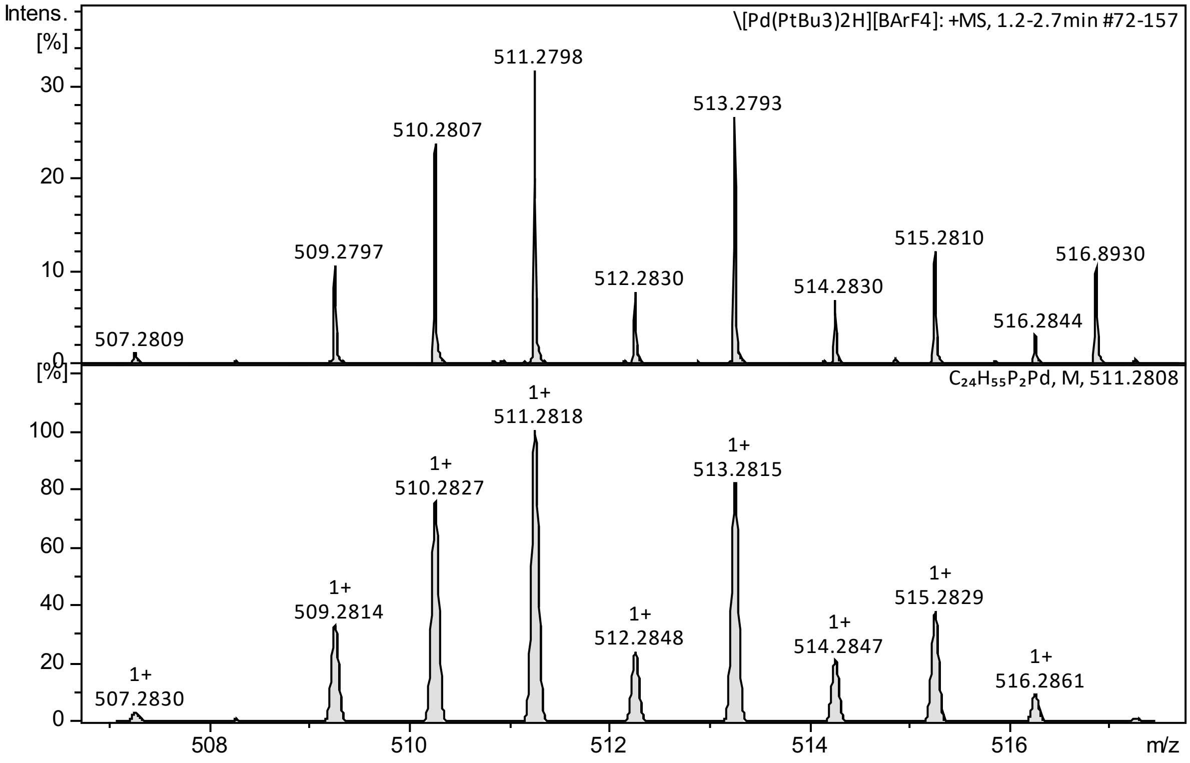

Figure S56. HR ESI-MS of **15**.

11 Characterisation of [Pt(PAd$_3$)$_2$H][BAr^F$_4$] (16)

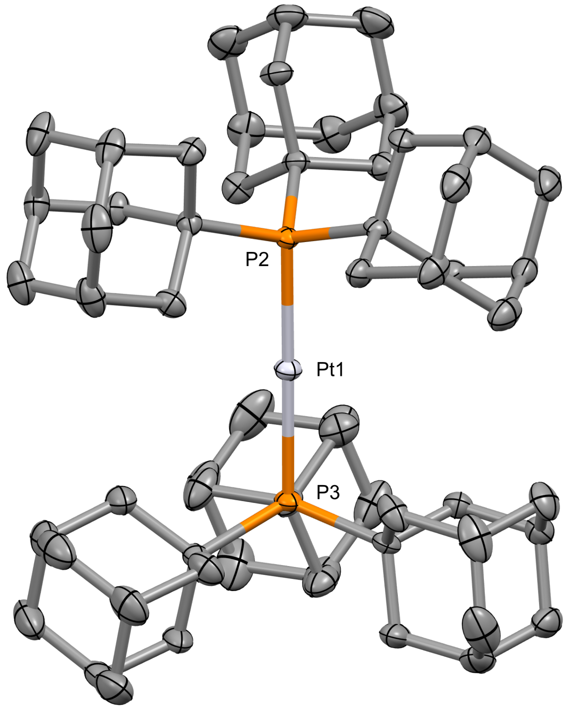

Figure S57. Solid-state structure of **16**. The hydride ligand was not located off the Fourier difference map. Thermal ellipsoids drawn at 50% probably; hydrogen atoms, solvent molecules, and anion omitted for clarity. Selected data: Pt1-P2, 2.3205(8) Å; Pt1-P3, 2.3219(8) Å; P2-Pt1-P3, 179.58(3)°.

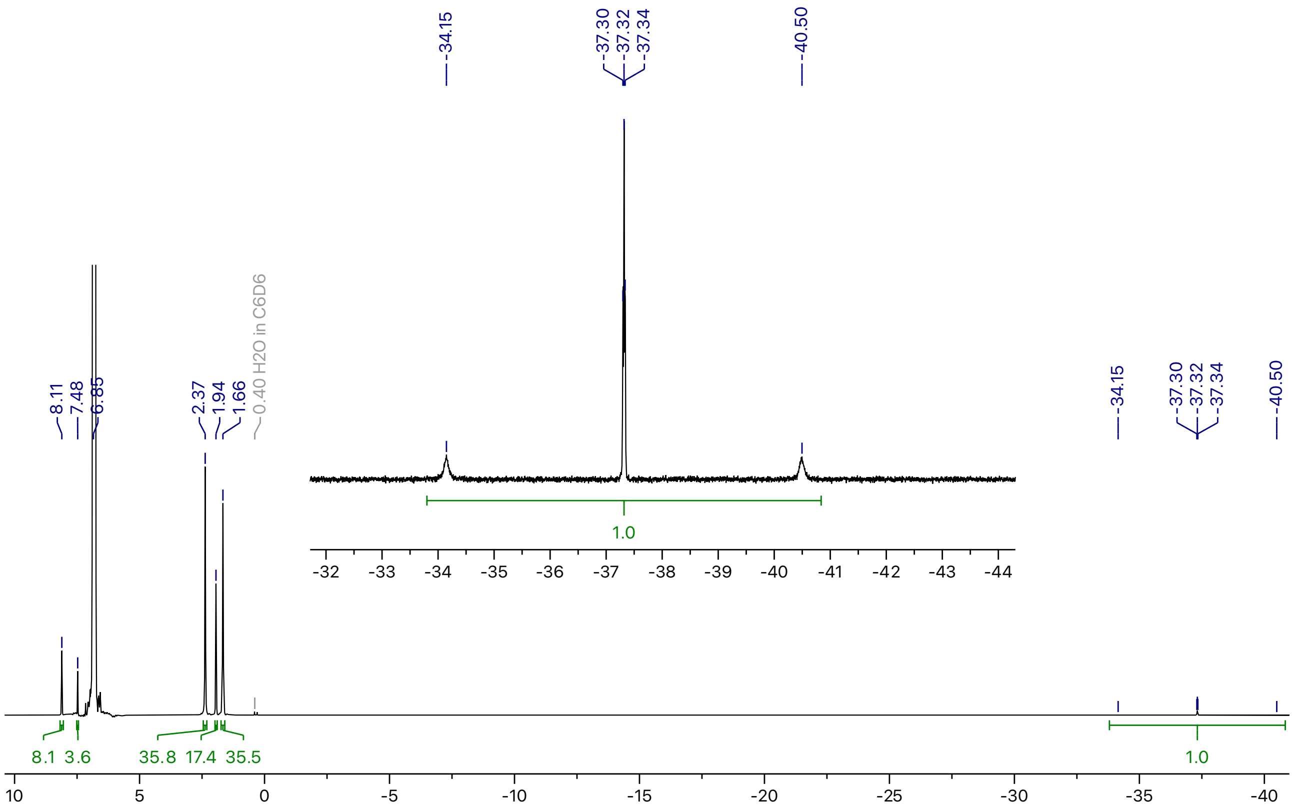

Figure S58. ^{1}H NMR spectrum of **16** (400 MHz, DFB).

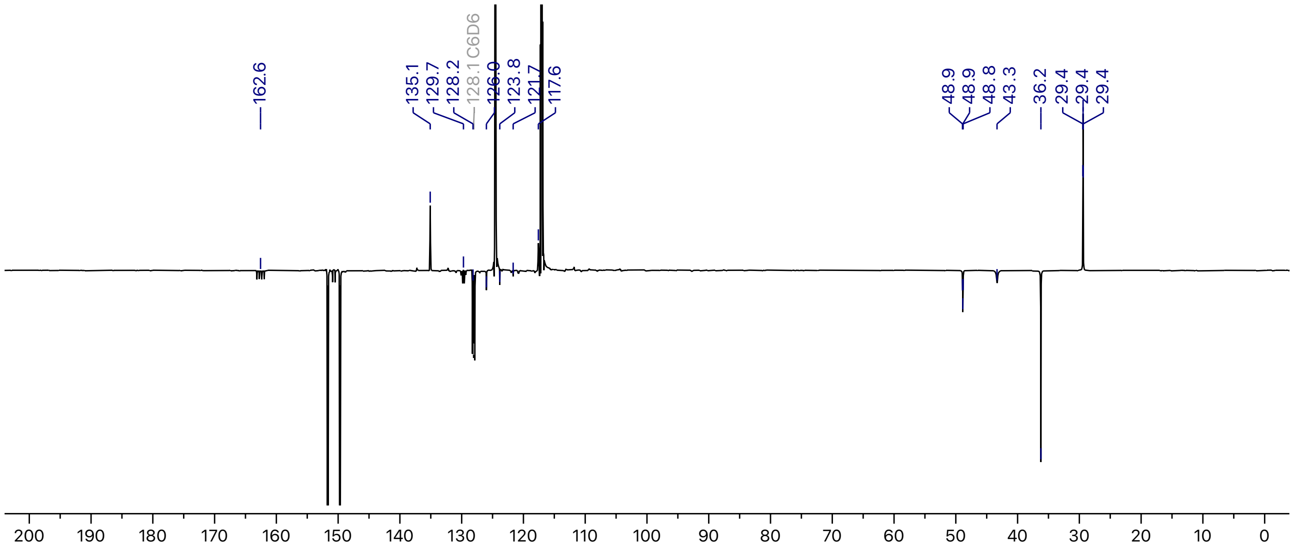

Figure S59. $^{13}C\{^1H\}$ APT NMR spectrum of **16** (126 MHz, DFB).

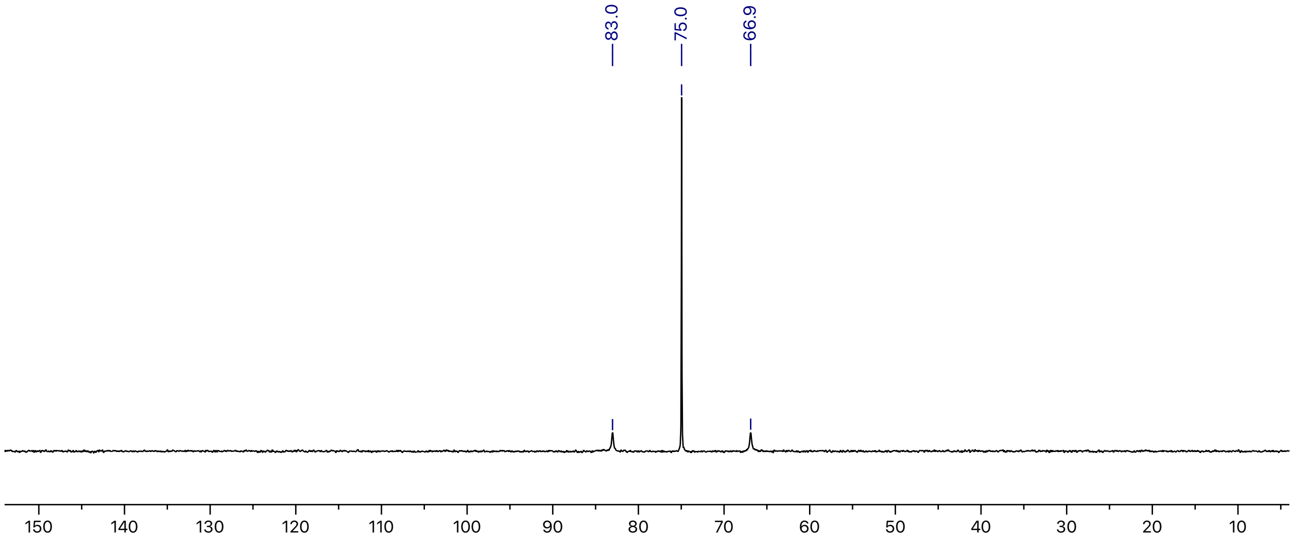

Figure S60. $^{31}P\{^1H\}$ NMR spectrum of **16** (162 MHz, DFB).

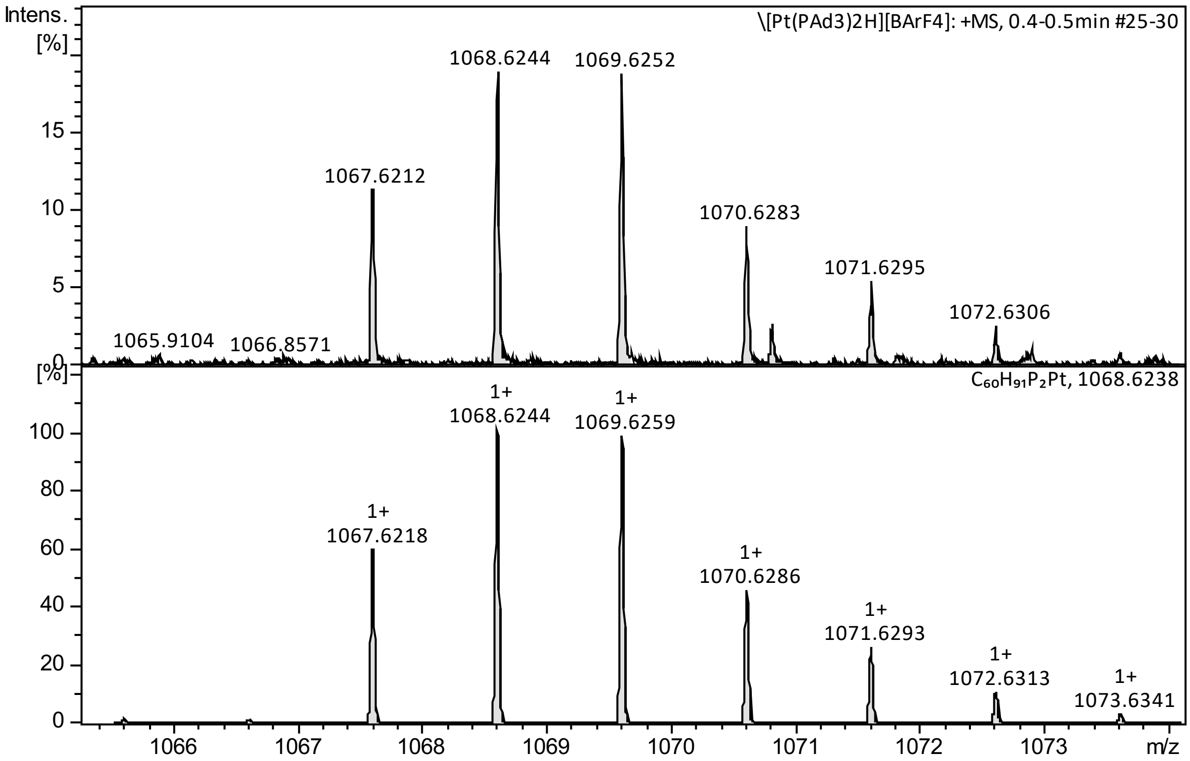

Figure S61. HR ESI-MS of **16**.

12 Computational details

12.1 Full details of Computational Methods

All electronic structure calculations presented in this paper were carried out using the ORCA 5.0.3 program package.[1] To find energetically most favorable conformers, initial conformer searches of selected complexes were conducted using the automated *crest* approach,[2] which employs the efficient semiempirical extended tight binding method (GFN2-xTB) with a specially adapted implicit solvation model (ALPB).[3] Unconstrained geometry optimizations in C_1 symmetry and analytical (or numerical for the largest systems) frequency calculations of all compounds were carried out at the DFT level, using the PBEh-3c composite method.[4] This method is based on a reparametrized hybrid variant of the PBE GGA exchange-correlation functional containing 42% non-local Hartree-Fock exchange. The orbitals are expanded in a modified valence double-ζ Gaussian basis corresponding to the Ahlrichs-type def2-mSVP set, in conjunction with the def2/J auxiliary basis set for the RI approximation to the Coulomb term.[5] The calculations utilized the def2-ECP for Pd (replacing 28 core electrons) and Pt (replacing 60 core electrons).[6] To account for inter- and intramolecular basis set superposition error (BSSE) and long-range London dispersion effects, the geometrical counterpoise correction (gCP)[7] and atom-pairwise DFT-D3 (Becke-Johnson damping) schemes are utilized.[8] PBEh-3c provides a computationally robust and cost-effective approach for efficient and accurate geometry optimizations and thermochemical properties of large molecules. The PBE hybrid functional itself performs well in terms of reproducing geometries for 1st, 2nd and 3rd row transition metal complexes.[9] Transition states were located by first performing relaxed energy scans of suitable geometrical parameters or alternatively by partially constrained preoptimization of approximate guess structures followed by analytical frequency calculations. If deemed suitable, the

obtained guess geometries were then subjected to full transition state optimisations. All optimized stationary points were characterized by analysis of their analytical second derivatives, with minima having only positive eigenvalues and transition states having one imaginary eigenvalue. Few optimised minima of encounter complexes contain spurious and vanishingly small imaginary frequencies ($<i10$ cm^{-1}) that could not be eliminated even with tighter geometry convergence or integration grid criteria. These are attributed to the size of the systems and indicative of numerical noise, but do not impact on the conclusions drawn. The nature of transition states was confirmed via intrinsic reaction coordinate (IRC) calculations in both forward and reverse direction of the reaction coordinate.[10] Subsequent geometry optimizations of the IRC end points yielded the nearest minima linked by a transition state. The frequency calculations also provided thermal and entropic corrections to the total energy in gas phase at T = 298.15 K and p = 1 atm within the quasi rigid-rotor/harmonic oscillator (QRRHO) approximation.[11] The open-shell singlet state (M_S = 0) corresponding to antiferromagnetically coupled metal centers in the bimetallic transition states and reactant precursor complexes were modeled with the help of the spin-unrestricted broken-symmetry (BS) formalism.[12] The FlipSpin feature of ORCA was used to generate initial guesses for the BS calculations. Geometries with these states were fully optimized and convergence to the desired BS solution was confirmed by inspection of magnetic orbitals, spin populations, and the expectation value of the $\langle S^2 \rangle$ operator. The energies of the BS states were used without spin projection. Initial test calculations revealed that PBEh-3c contains adequate amounts of Hartree–Fock exchange admixture that allow stabilisation of the antiferromagnetically coupled local S = ½ spin centres in the dinuclear systems. The validity of several density functionals (B3LYP[13], ωB97X-D3BJ[14], ωB97X-V[15], B2PLYP-D3(BJ)[16]) to generate reliable single-point energies for the given systems was tested against results obtained from high-level *ab initio* DLPNO-CCSD(T) calculations,[17] using the T_0 algorithm to obtain perturbative triples excitations. The three truncation parameters which define cutoffs for occupation numbers in the pair natural orbitals (T_{CutPNO}), estimated pair correlation energies ($T_{CutPairs}$), and for the fitting domain selection (T_{CutMKN}) were chosen according to default settings. These self-consistent field calculations were performed using the RIJCOSX approximation with a convergence criterion of 10^{-9} E_h (VeryTightSCF) and in conjunction with the def2-TZVPP basis set on all atoms (and associated def2-ECP for Pt) supported by the corresponding def2-TZVPP/C and def2/J auxiliary bases. Single point energies were computed using the B2PLYP-D3(BJ) double hybrid functional (including Grimme's D3 atom-pairwise dispersion correction and Becke-Johnson damping) in combination with the def2-TZVPP basis set.[18] The RI-MP2 variant was invoked by prepending the RI prefix to the functional name, and the def2-TZVPP/C auxiliary basis set was specified for correlation fitting.[19] The chain of spheres approximation to exact exchange (COSX)[20] was used in combination with the def2/J auxiliary basis. The SCF energy convergence was set to "tight". Effects due to the presence of a solvent were treated implicitly with a conductor-like polarizable continuum (CPCM) and Truhlar's SMD model.[21] Solvent parameters corresponded to those of tetrahydrofuran (ε = 7.4, refractive index = 1.000), or, in the absence of defined parameters for DFB solvent, default SMD parameters were selected for fluorobenzene and the dielectric constant adjusted to that of DFB (ε = 13.4, refractive index = 1.443). For reactions involving THF solvent molecules explicitly, computed energies were adjusted to incorporate effects from excess solvent following literature procedures.[22] In particular to adjust for the transfer of solute

from a 1 atm in gas phase to 1 M standard state in solution: $\Delta G^{\circ\rightarrow *} = RT\ln(24.46)$; a correction for the standard state of the solvated THF solvent: $\Delta G^{*\rightarrow conc} = RT\ln(12.31/n)$, where n is the number of THF molecules; a correction for the experimental concentration differential $\Delta G^{\circ\rightarrow\circ'} = RT\ln(0.02/12.31)$, with a [**4**$^+$] = 20 mM. Absorption properties of were calculated using time-dependent DFT (TD) with the Tamm-Dancoff (TDA) approximation. The long-rage corrected CAM-B3LYP hybrid functional[23] was employed in combination with a def2-TZVP basis set on all atoms, including the corresponding def2-ECPs for Pt and Pd. Effects from DFB solvent were accounted for through the linear response CPCM/SMD scheme.[24] Representations of spectra were generated by convoluting calculated transition energies and intensities with Gaussian functions with a full width at half-maximum (fwhm) of 2000 cm^{-1}. Geometries were visualized using the ChemCraft software package.[25]

12.2 Structure and properties of [M(P*t*Bu$_3$)$_2$]$^+$ (M = Pd, 3$^+$; Pt, 4$^+$).

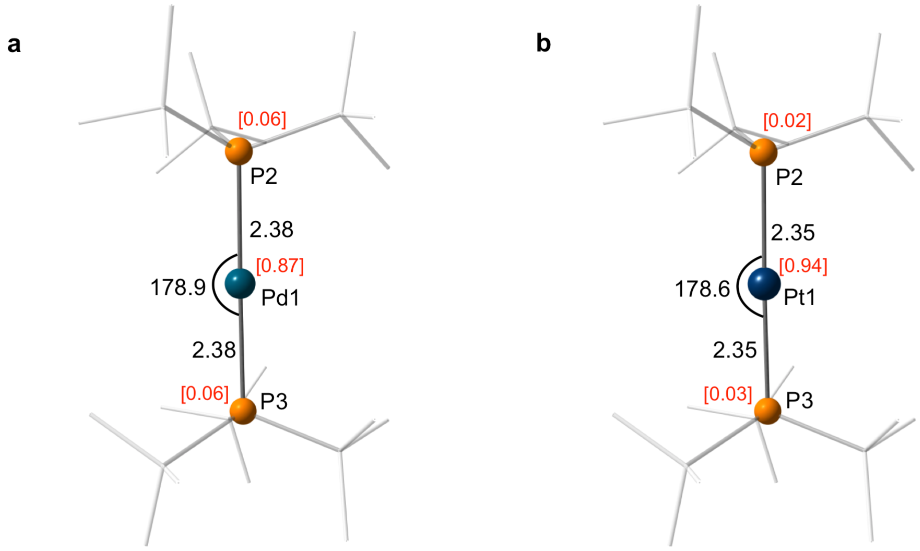

Figure S62. Optimised geometries of (a) **3**$^+$ (S = ½) and (b) **4**$^+$ (S = ½) at the PBEh-3c level of theory with key bond distances (Å), angles (°), and spin populations (red numbers in square brackets).

Table S1. Comparison between DFT-optimised and experimental structural parameters.

	PBEh-3c	B3LYP-D3(BJ)	X-ray
**1**			
Pd1–P2 / Å	2.306	2.299	
Pd1–P3 / Å	2.307	2.299	
P2–Pd1–P3 / °	177.7	179.7	
**2**			
Pt1–P2 / Å	2.284	2.279	
Pt1–P3 / Å	2.286	2.280	
P1–Pt1–P2 / °	178.3	178.4	
**3⁺**			
Pd1–P2 / Å	2.376	2.354	2.349(3)
Pd1–P3 / Å	2.379	2.355	2.353(3)
P2–Pd1–P3 / °	178.9	179.0	180.0
**4⁺**			
Pt1–P2 / Å	2.354	2.342	2.333(4)
Pt1–P3 / Å	2.354	2.341	2.338(4)
P1–Pt1–P2 / °	178.6	179.7	180.0
**5⁺**			
Pt1–P2 / Å	2.314	2.314	2.297(2)
Pt1–P3 / Å	2.367	2.346	
Pt1–C4 / Å	2.041	2.057	2.063(17)
P2–Pt1–P3 / °	174.2	175.4	180.0
Pt1–P3–C3 / °	84.1	84.6	90.0(3)

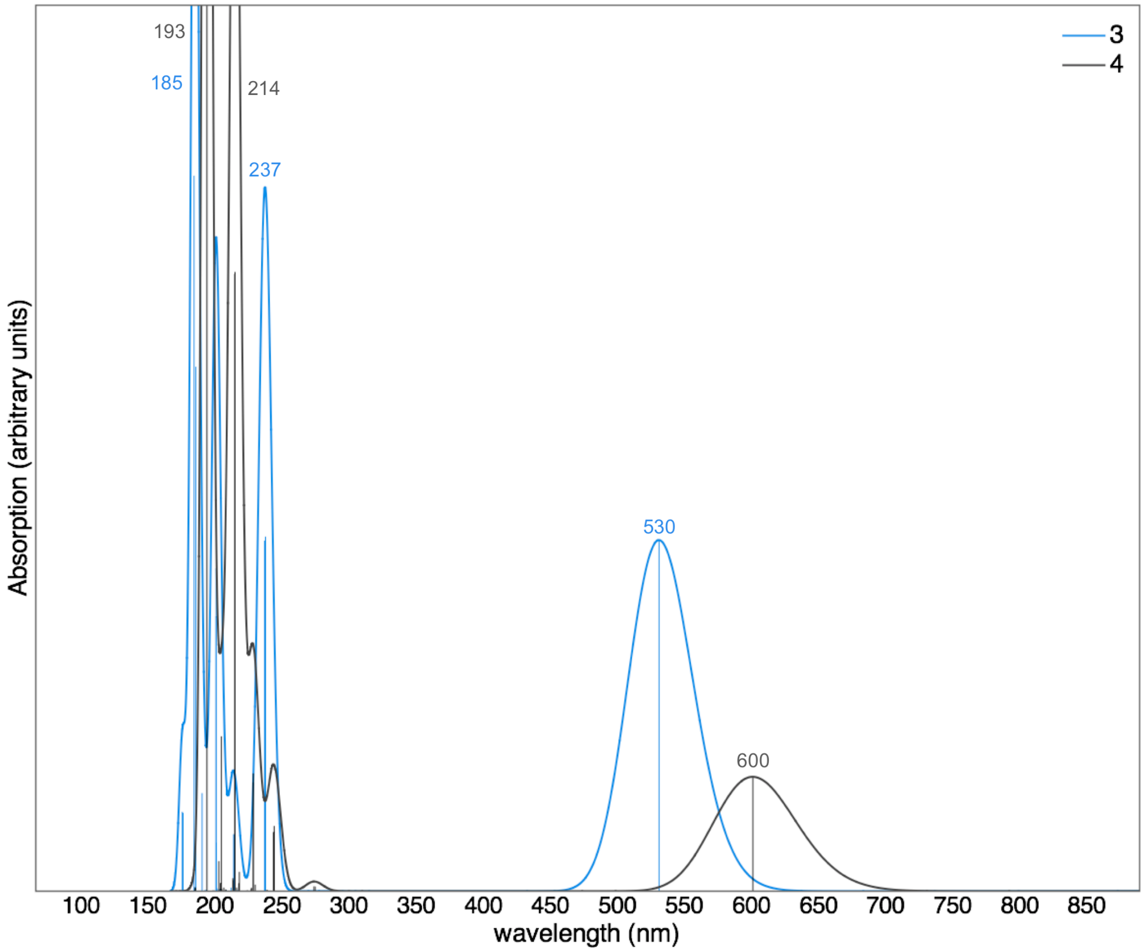

Figure S63. Calculated UV-vis spectra of **3**$^{+}$ and **4**$^{+}$ at the CAM-B3LYP-D3(BJ)/def2-TZVP+def2-ECP(Pd/Pt)) level of theory. Absorption energies include corrections due to effects from DFB solvent.

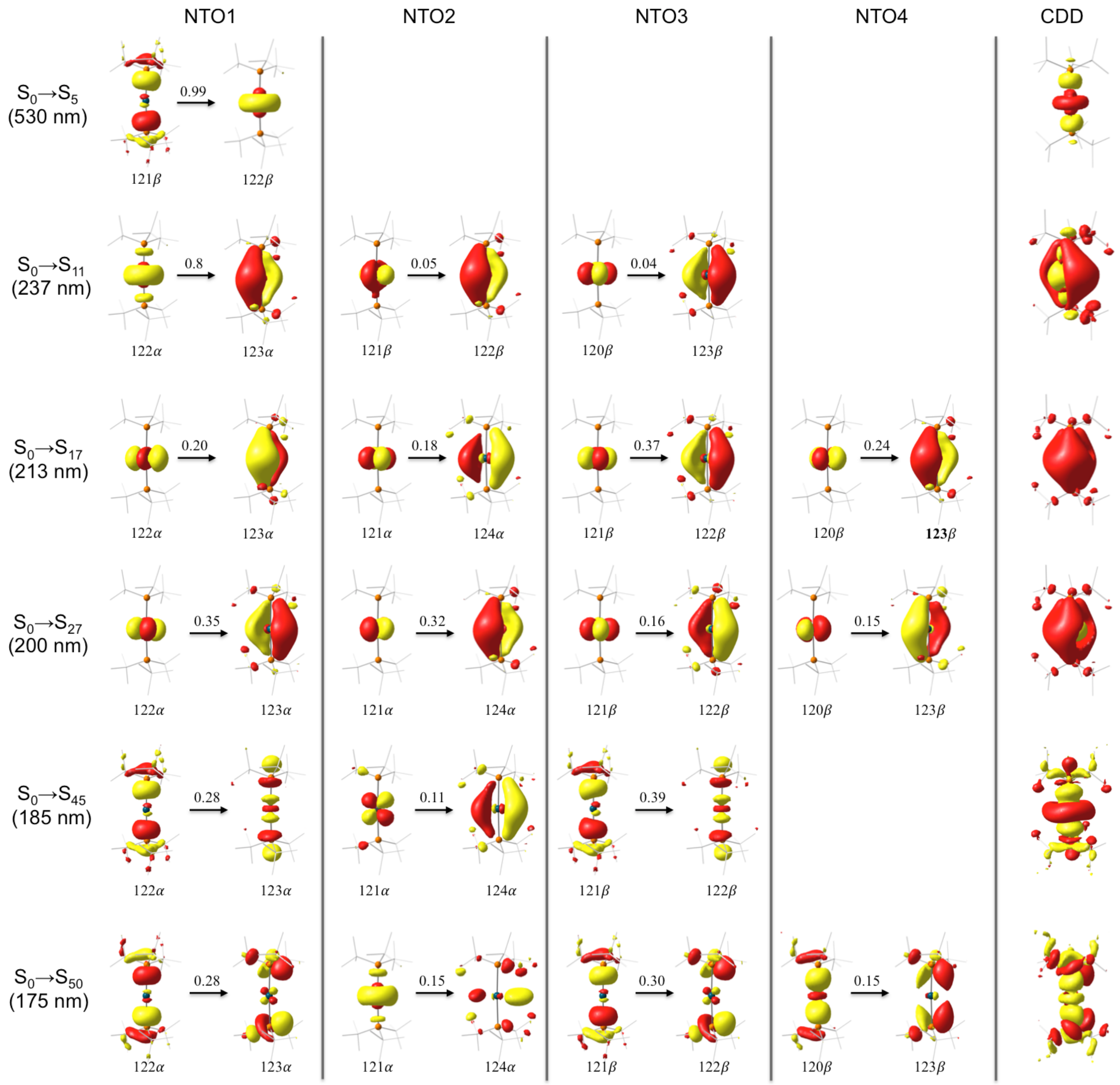

Figure S64. Donor/acceptor pairs of natural transition orbitals (NTOs) and charge difference densities (CDDs, red: charge accumulation, yellow, charge depletion) for **3**$^+$ (isosurface 0.05 au)**.**

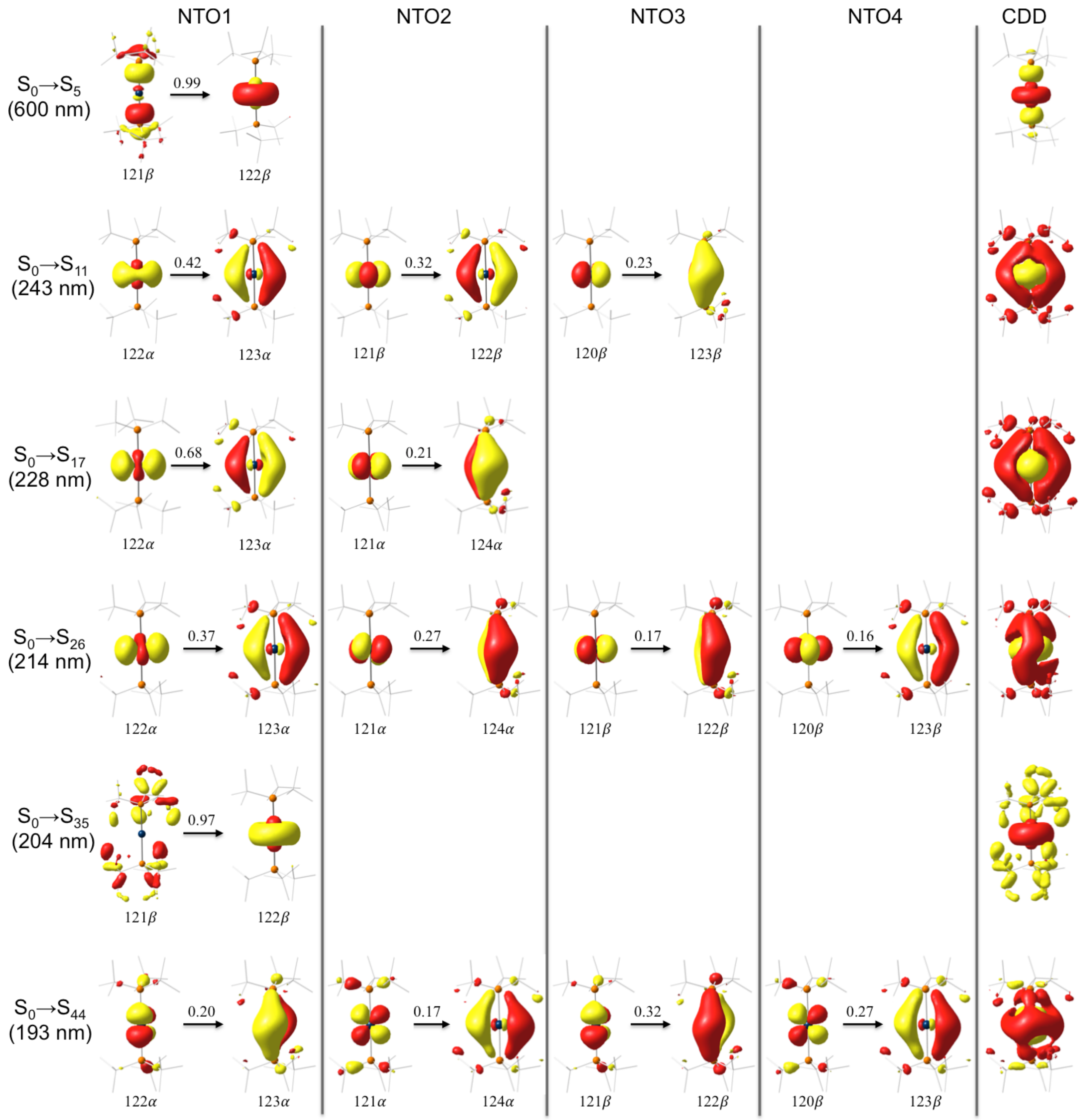

Figure S65. Donor/acceptor pairs of natural transition orbitals (NTOs) and charge difference densities (CDDs, red: charge accumulation, yellow, charge depletion) for **4⁺** (isosurface 0.05 au).

12.3 Reactions of [M(P*t*Bu$_3$)$_2$]$^+$ (M = Pd, 3$^+$; Pt, 4$^+$).

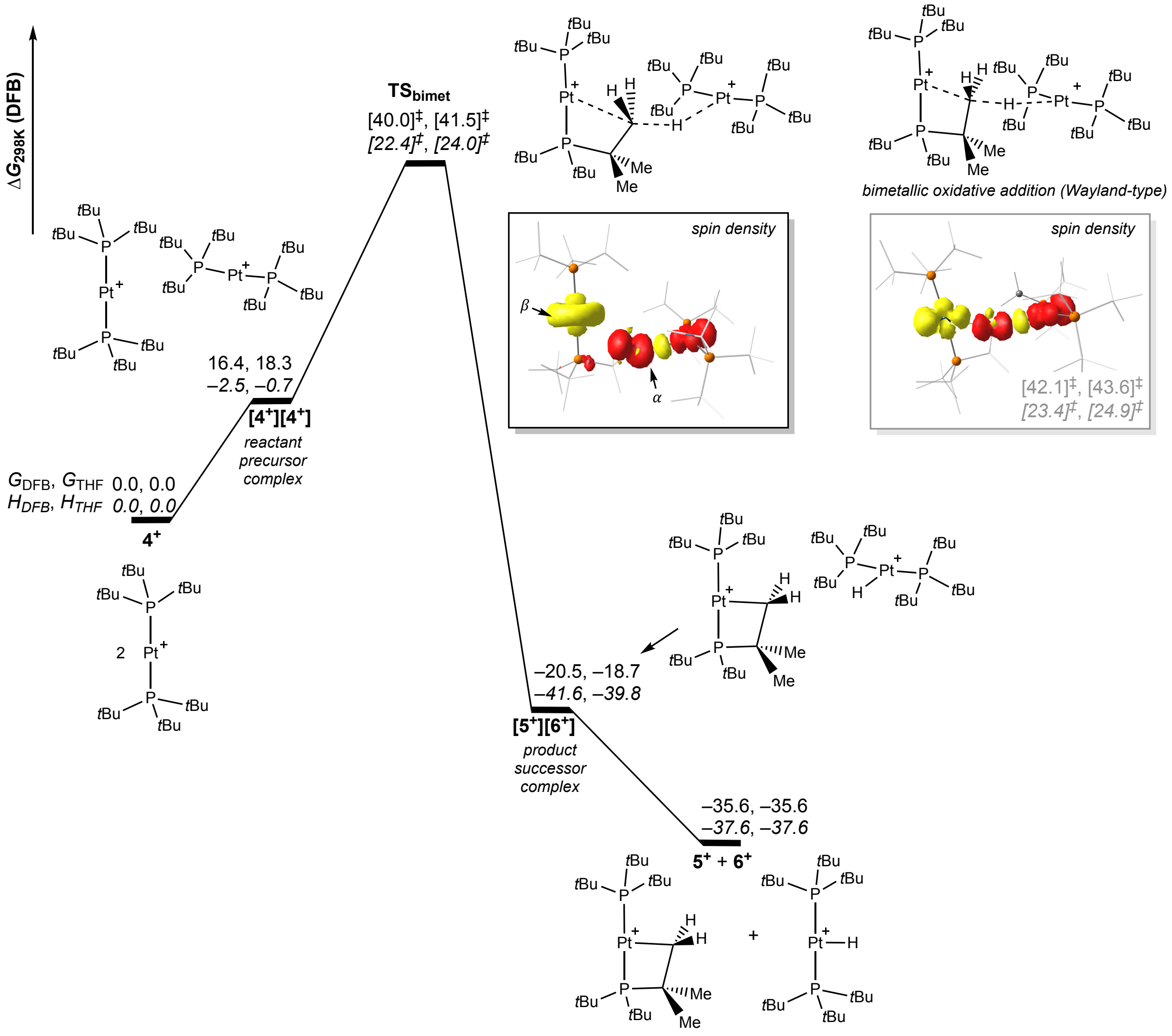

Figure S66. Computed reaction profile for bimetallic C–H bond oxidative addition to **4$^+$** at the B2PLYP-D3(BJ)/def2-TZVPP+def-ECP(Pt)//PBEh-3c level of theory. Energies corrected for DFB and THF solvent. The left inset depicts the spin density of the transition state (isosurface 0.005 au). The right inset shows the spin density of the partially optimised transition state guess before unconstrained saddle point geometry optimisation. It contains one imaginary mode (*i*1348 cm^{-1}) and resembles the Wayland-type diradical oxidative addition, but still high in energy.

Table S2. Calculated thermodynamics and kinetics for bimetallic C–H bond oxidation in **4⁺**. Relative Gibbs free energies (enthalpies in parentheses) at different levels of theory (corrected for DFB solvent) on PBEh-3c optimised geometries (kcal·mol^{-1}).

Method	4^+	$[4^+][4^+]$	TS_{bimet}	$[5^+][6^+]$	$5^+ + 6^+$
B2PLYP-D3(BJ)	0.0<br>(0.0)	18.6<br>(–0.9)	40.0<br>(22.4)	–20.5<br>(–38.5)	–35.6<br>(–37.6)
B3LYP-D3(BJ)	0.0<br>(0.0)	18.3<br>(–1.3)	38.0<br>(20.5)	–10.9<br>(–29.0)	–25.7<br>(27.7)
ωB97X-D3(BJ)	0.0<br>(0.0)	18.6<br>(–1.00)	50.8<br>(33.3)	–8.9<br>(–26.9)	–23.8<br>(–25.8)
ωB97X-V	0.0<br>(0.0)	18.2<br>(–1.4)	50.5<br>(33.0)	–7.4<br>(–25.4)	–22.3<br>(–24.4)

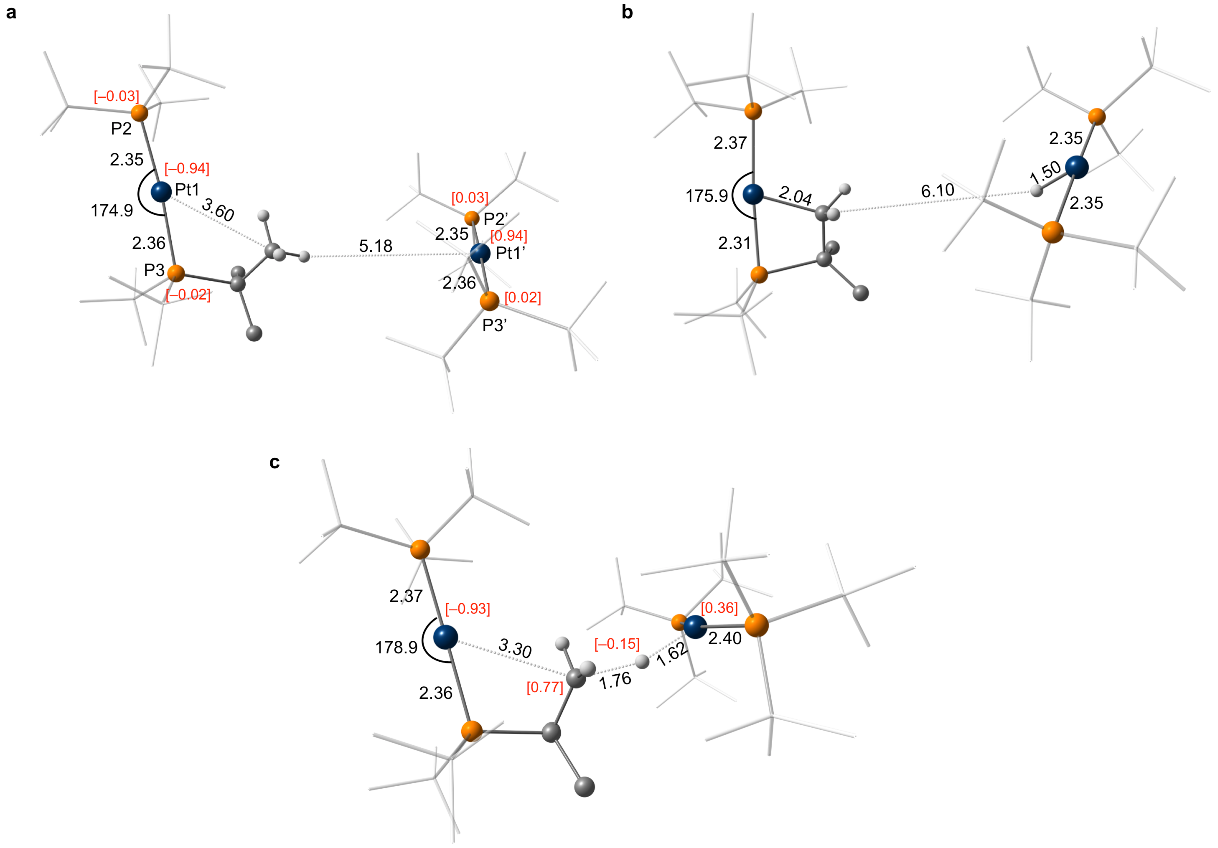

Figure S67. Optimised geometries of (a) reactant precursor complex **[4⁺][4⁺]** (M_S = 0), (b) product successor complex **[5⁺][6⁺]** (S = 0), and (c) biradical transition state $\mathbf{TS_{bimet}}$ (M_S = 0) with key bond distances (Å), angles (°), and spin populations (red numbers in square brackets).

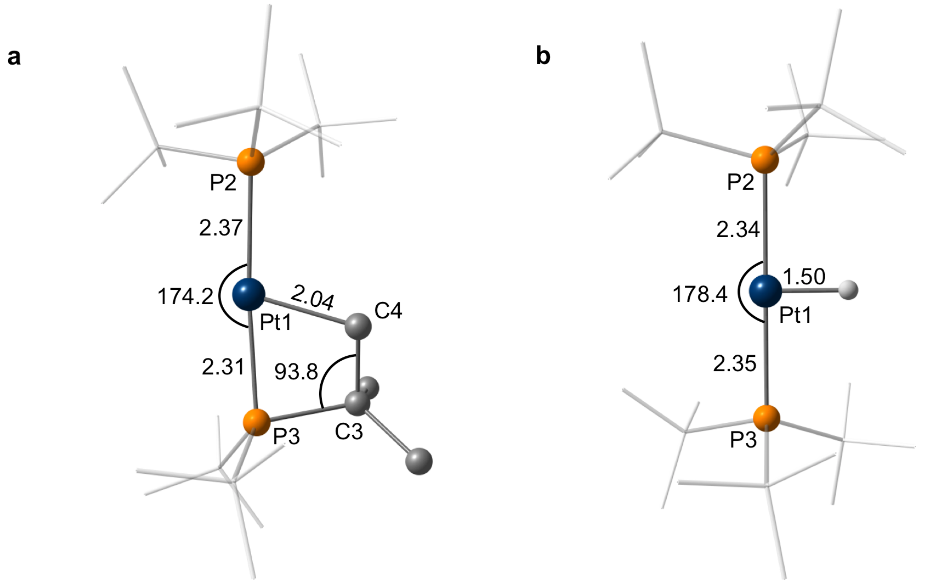

Figure S68. Optimised geometries of (a) **5**$^+$ (S = 0) and (b) **6**$^+$ (S = 0) at the PBEh-3c level of theory with key bond distances (Å) and angles (°).

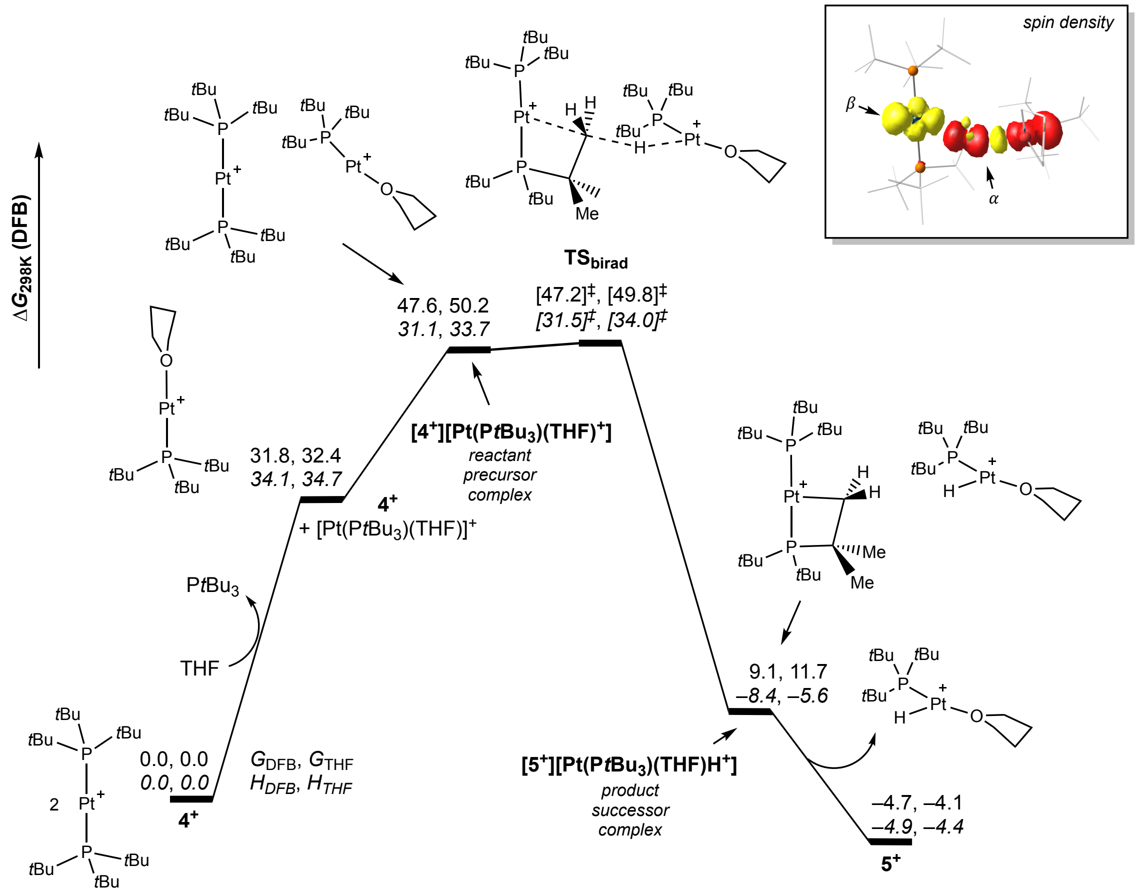

Figure S69. Computed reaction profile for biradical C–H bond oxidative addition to **4**$^+$ and **[Pt(P*t*Bu$_3$)(THF)]$^+$** at the B2PLYP-D3(BJ)/def2-TZVPP+def-ECP(Pt)//PBEh-3c level of theory. Energies corrected for DFB and THF solvent. The inset depicts the spin density of the transition state (isosurface 0.005 au).

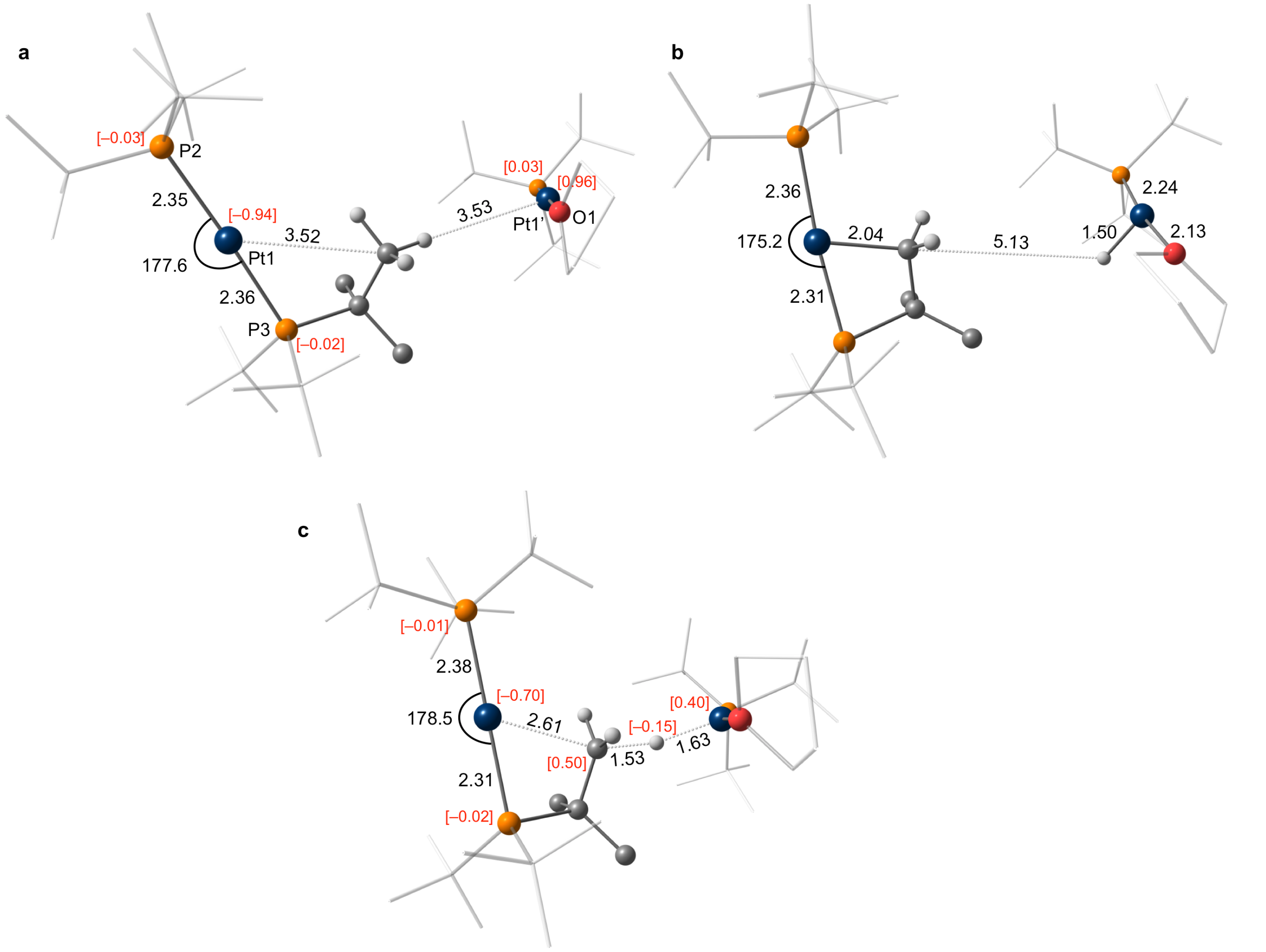

Figure S70. Optimised geometries of (a) reactant precursor complex **[4$^+$][Pt(P*t*Bu$_3$)(THF)$^+$]** (M_S = 0), (b) product successor complex **[5$^+$][Pt(P*t*Bu$_3$)(THF)H$^+$]** (S = 0), and (c) biradical transition state **TS$_{\mathbf{birad}}$** (M_S = 0) with key bond distances (Å), angles (°), and spin populations (red numbers in square brackets).

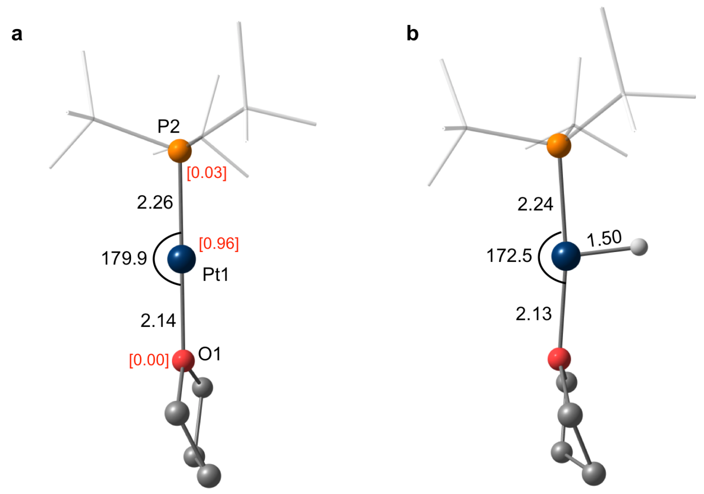

Figure S71. Optimised geometries of (a) **[Pt(P*t*Bu$_3$)(THF)]$^+$** (S = ½) and (b) **[Pt(P*t*Bu$_3$)(THF)H]$^+$** (S = 0) with key bond distances (Å), angles (°), and spin populations (red numbers in square brackets).

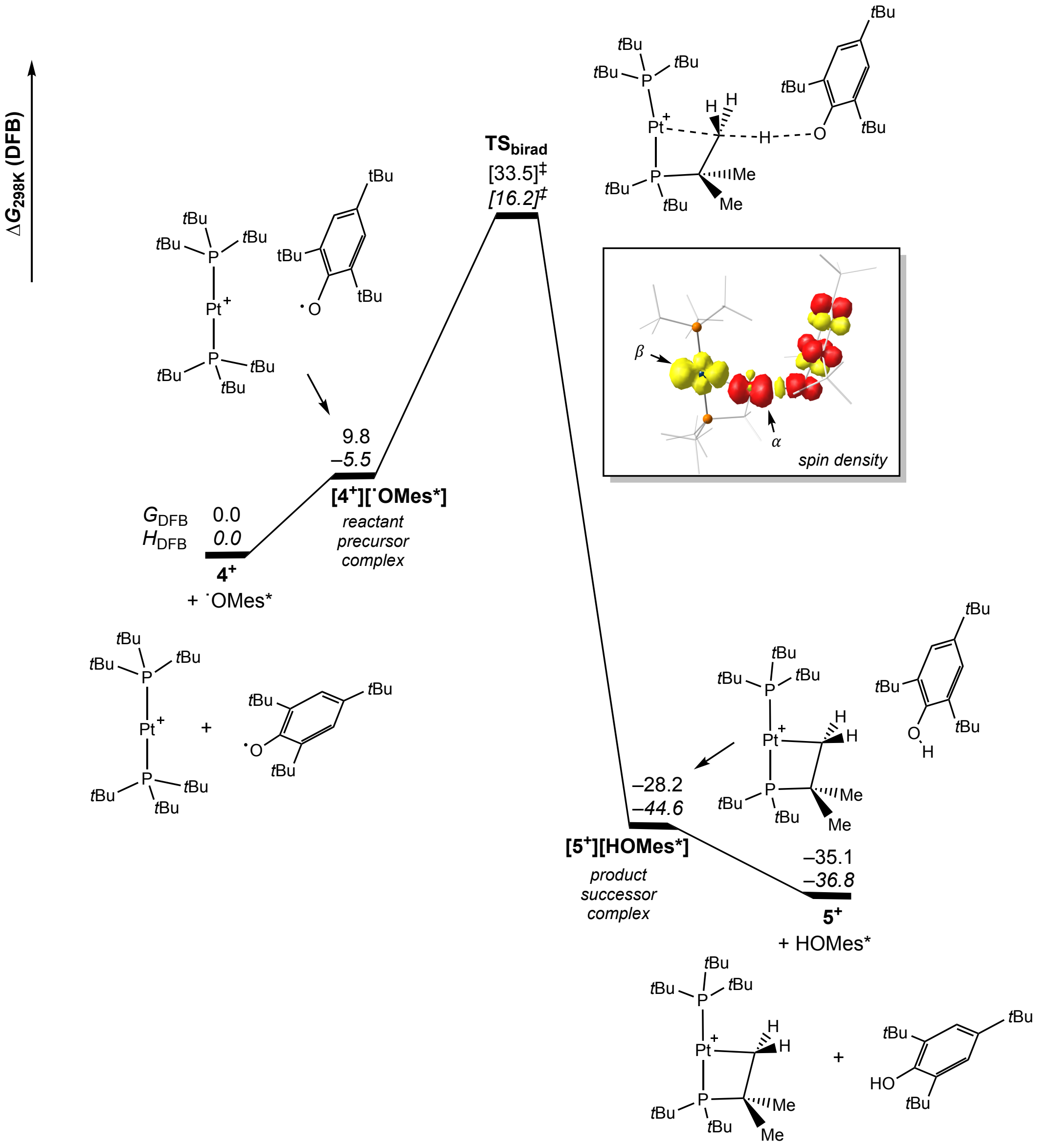

Figure S72. Computed reaction profile for biradical C–H bond oxidative addition to **4⁺** and •OMes* at the B2PLYP-D3(BJ)/def2-TZVPP+def-ECP(Pt)//PBEh-3c level of theory. Energies corrected for DFB solvent. The inset depicts the spin density of the transition state (isosurface 0.005 au).

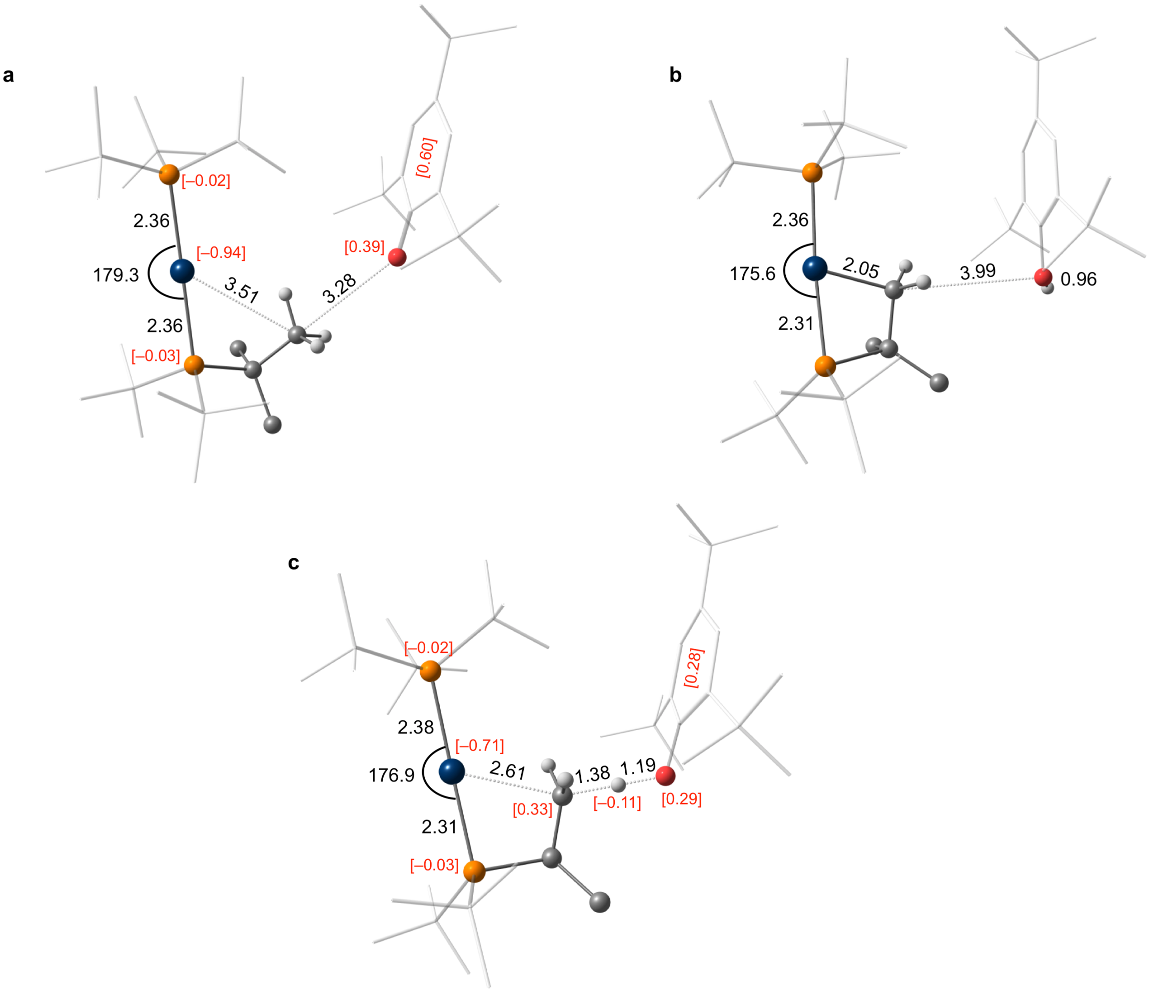

Figure S73. Optimised geometries of (a) reactant precursor complex **[4⁺][OMes*]** (M_S = 0), (b) product successor complex **[5⁺][HOMes*]** (S = 0) and (c) biradical transition state **TS$_{HAT}$** (M_S = 0) with key bond distances (Å), angles (°), and spin populations (red numbers in square brackets).

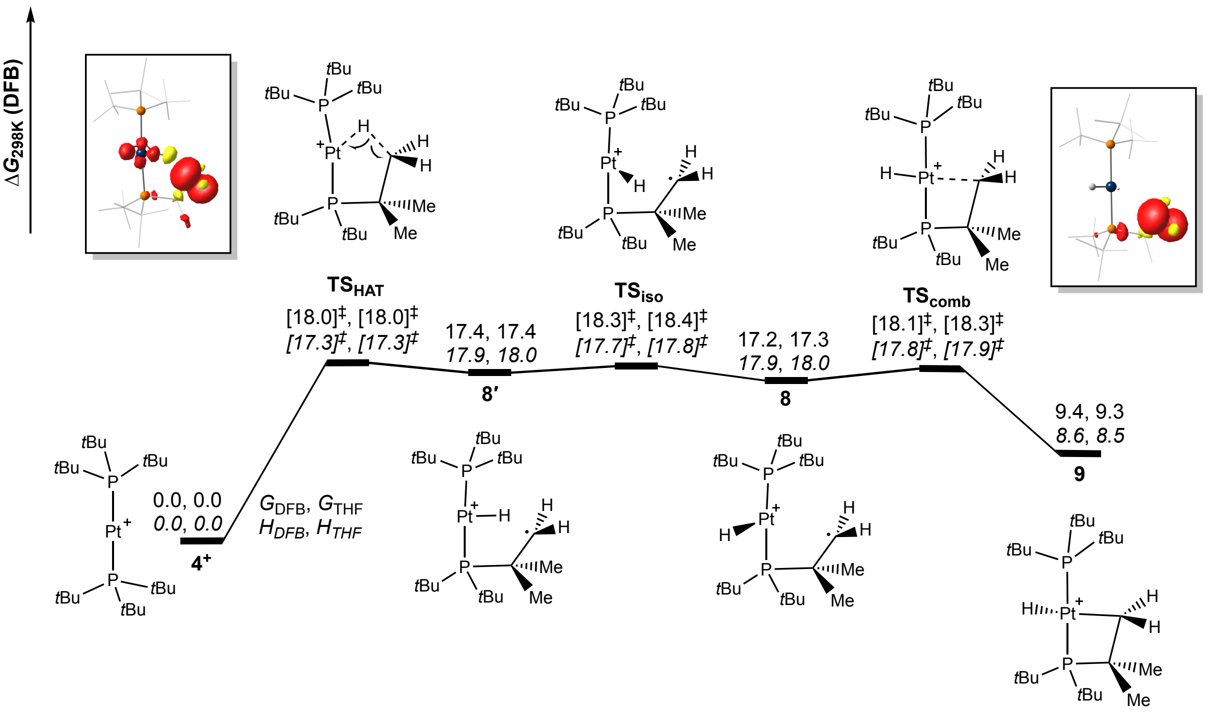

Figure S74. Computed reaction profile for step-wise intramolecular C–H bond activation in **4⁺** at the B2PLYP-D3(BJ)/def2-TZVPP+def-ECP(Pt)//PBEh-3c level of theory. Energies corrected for DFB and THF solvent. The insets depict the spin density of **TS$_{HAT}$** and **TS$_{comb}$** (isosurface 0.005 au).

Table S3. Calculated thermodynamics and kinetics for step-wise intramolecular C–H bond activation in **4⁺**. Relative Gibbs free energies (enthalpies in parentheses) at different levels of theory (corrected for DFB solvent) on PBEh-3c optimised geometries (kcal·mol^{-1}).

Method	4⁺	TS$_{HAT}$	8′	TS$_{iso}$	8	TS$_{comb}$	9
B2PLYP-D3(BJ)	0.0 (0.0)	18.0 (17.3)	17.4 (17.9)	18.3 (17.7)	17.2 (17.9)	18.1 (17.8)	9.4 (8.6)
B3LYP-D3(BJ)	0.0 (0.0)	20.3 (19.6)	19.7 (20.3)	20.6 (20.0)	19.5 (20.3)	20.5 (20.2)	13.9 (13.1)
ωB97X-D3(BJ)	0.0 (0.0)	25.1 (24.4)	23.3 (23.9)	24.3 (23.7)	23.1 (23.8)	24.3 (23.9)	15.9 (15.1)
ωB97X-V	0.0 (0.0)	26.0 (25.3)	24.2 (24.8)	24.9 (24.3)	23.7 (24.4)	24.9 (24.5)	16.5 (15.7)
DLPNO-CCSD(T)	0.0 (0.0)	19.7 (19.0)	18.0 (18.5)	18.9 (18.3)	18.0 (18.7)	19.1 (18.7)	10.7 (9.9)

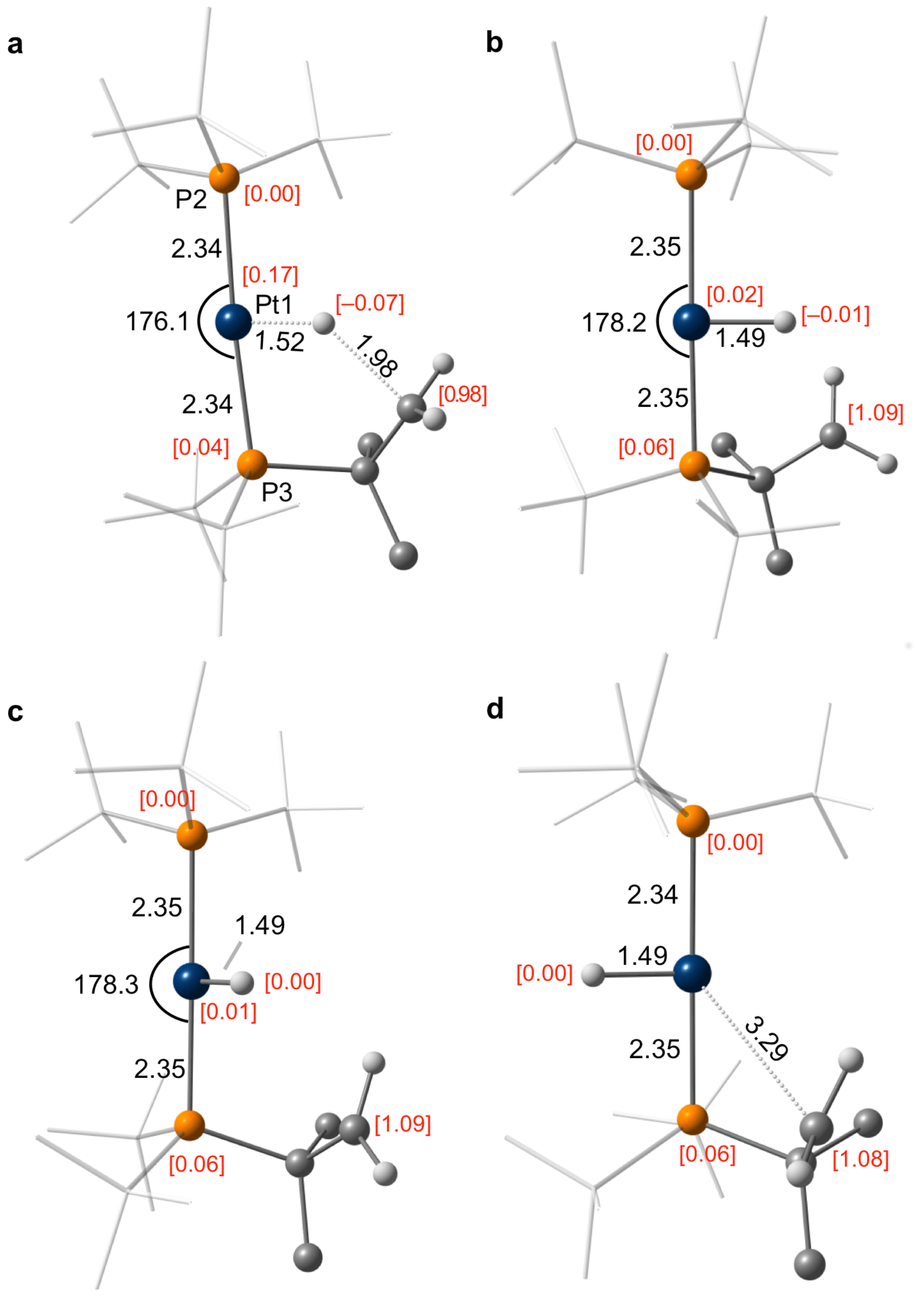

Figure S75. Optimised geometries of (a) **TS$_{HAT}$** (S = ½), (b) **8′** (S = ½), (c) **8** (S = ½), and (d) **TS$_{comb}$** (S = ½) at the PBEh-3c level of theory with key bond distances (Å), angles (°), and spin populations (red numbers in square brackets).

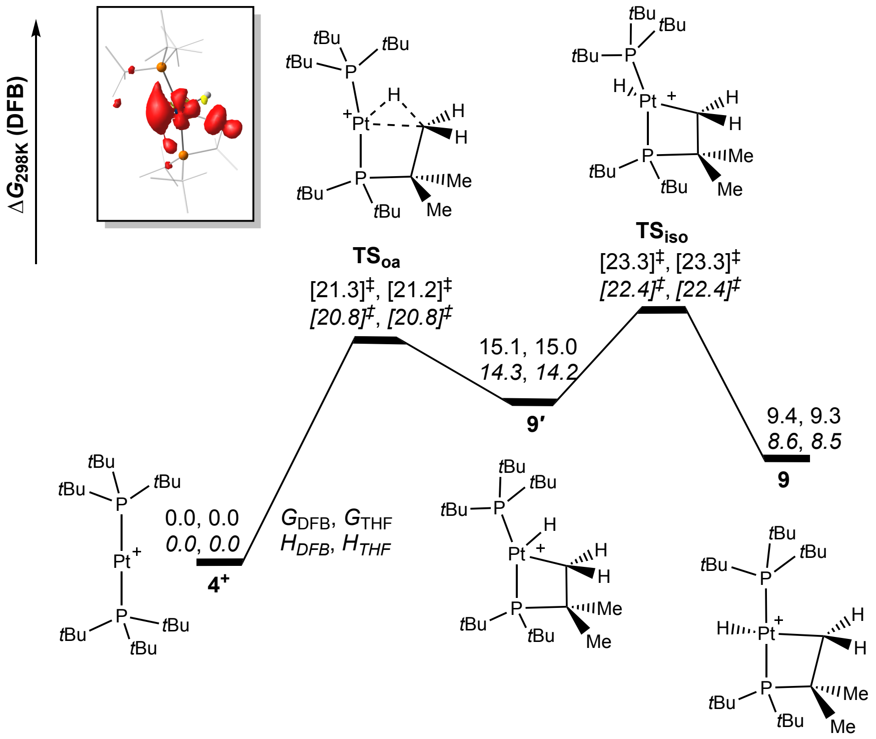

Figure S76. Computed reaction profile for concerted intramolecular C–H bond activation in **4⁺** at the B2PLYP-D3(BJ)/def2-TZVPP+def-ECP(Pt)//PBEh-3c level of theory. Energies corrected for DFB and THF solvent. The inset depicts the spin density of TS_{OA} (isosurface 0.005 au).

Table S4. Calculated thermodynamics and kinetics for concerted intramolecular C–H bond activation in **4⁺**. Relative Gibbs free energies (enthalpies in parentheses) at different levels of theory (corrected for DFB solvent) on PBEh-3c optimised geometries (kcal·mol^{-1}). [a]Geometry optimisation using B3LYP-D3(BJ) in conjunction with def2-TZVP on Pt(+ECP) and P, def2-SVP on C and H.

Method	**4⁺**	**TS$_{oa}$**	**9′**	**TS$_{iso}$**	**9**
B2PLYP-D3(BJ)	0.0 (0.0)	21.3 (20.8)	15.1 (14.3)	23.3 (22.4)	9.4 (8.6)
B2PLYP-D3(BJ)[a]	0.0 (0.0)	22.9 (22.2)	16.8 (15.6)	24.6 (23.4)	11.0 (9.9)
B3LYP-D3(BJ)	0.0 (0.0)	26.5 (26.1)	20.4 (19.6)	27.8 (26.9)	13.9 (13.1)
ωB97X-D3(BJ)	0.0 (0.0)	27.5 (27.2)	22.7 (21.9)	30.5 (29.6)	15.9 (15.1)
ωB97X-V	0.0 (0.0)	27.7 (27.2)	23.1 (22.3)	30.8 (29.9)	16.5 (15.7)
DLPNO-CCSD(T)	0.0 (0.0)	21.8 (21.3)	16.8 (16.0)	23.7 (22.8)	10.7 (9.9)

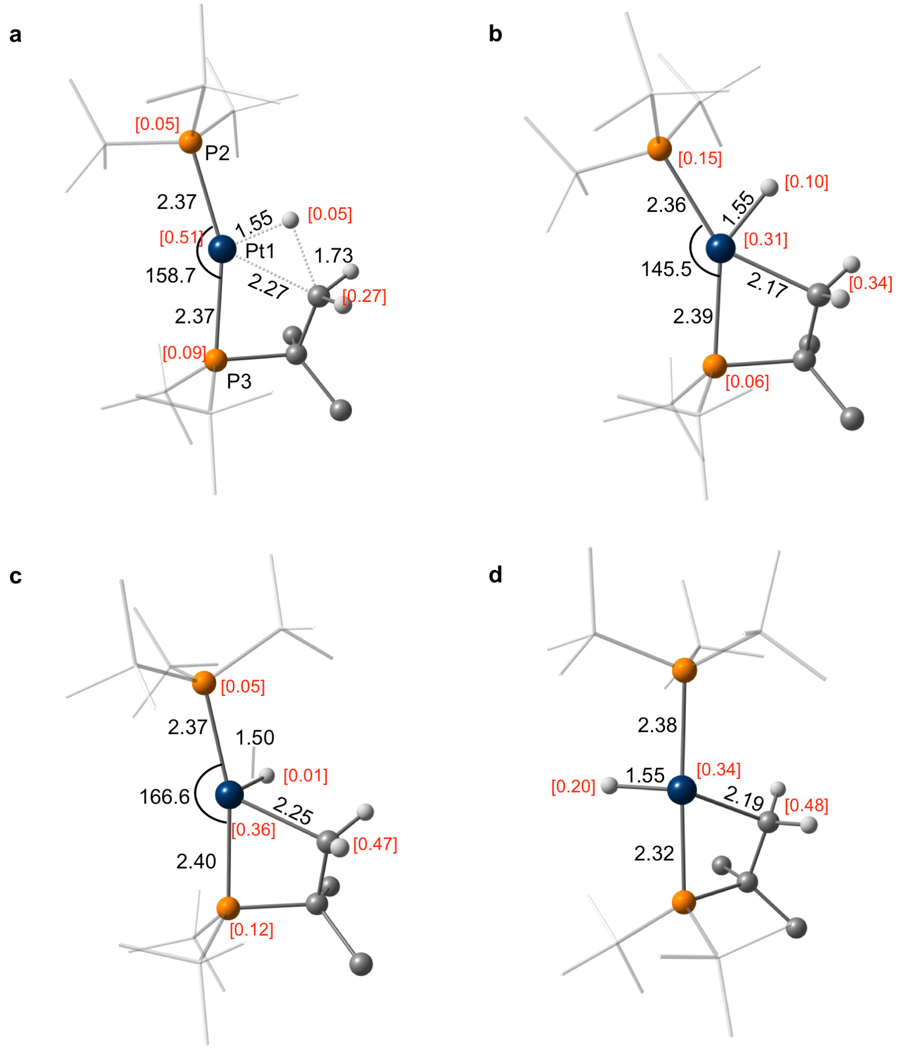

Figure S77. Optimised geometries of (a) **TS$_{oa}$** (S = ½), (b) **9′** (S = ½), (c) **TS$_{iso}$** (S = ½) and (d) **9** (S = ½) at the PBEh-3c level of theory with key bond distances (Å) and angles (°), and spin populations (red numbers in square brackets).

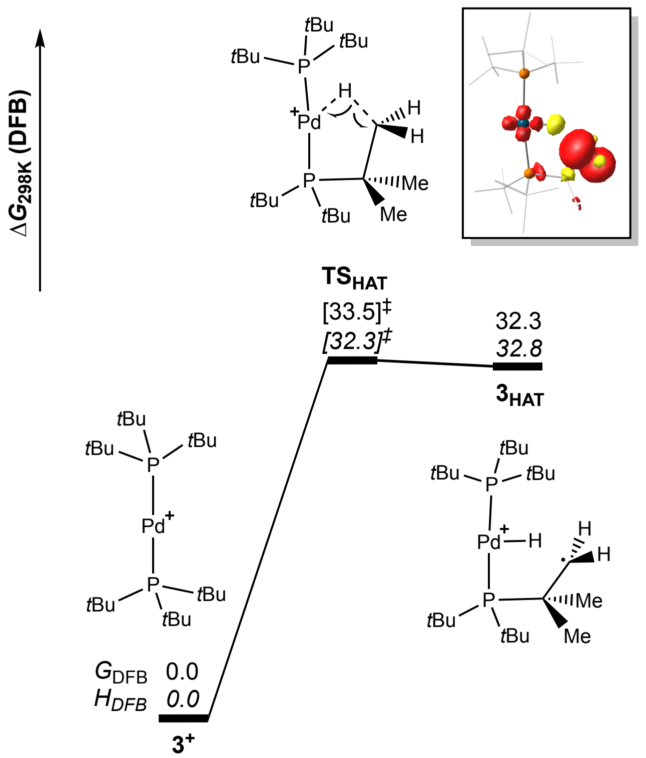

Figure S78. Computed reaction profile for step-wise intramolecular C–H bond activation in **3**$^+$ at the B2PLYP-D3(BJ)/def2-TZVPP+def-ECP(Pd)//PBEh-3c. Energies corrected for DFB solvent. The inset depicts the spin density of the transition state (isosurface 0.005 au).

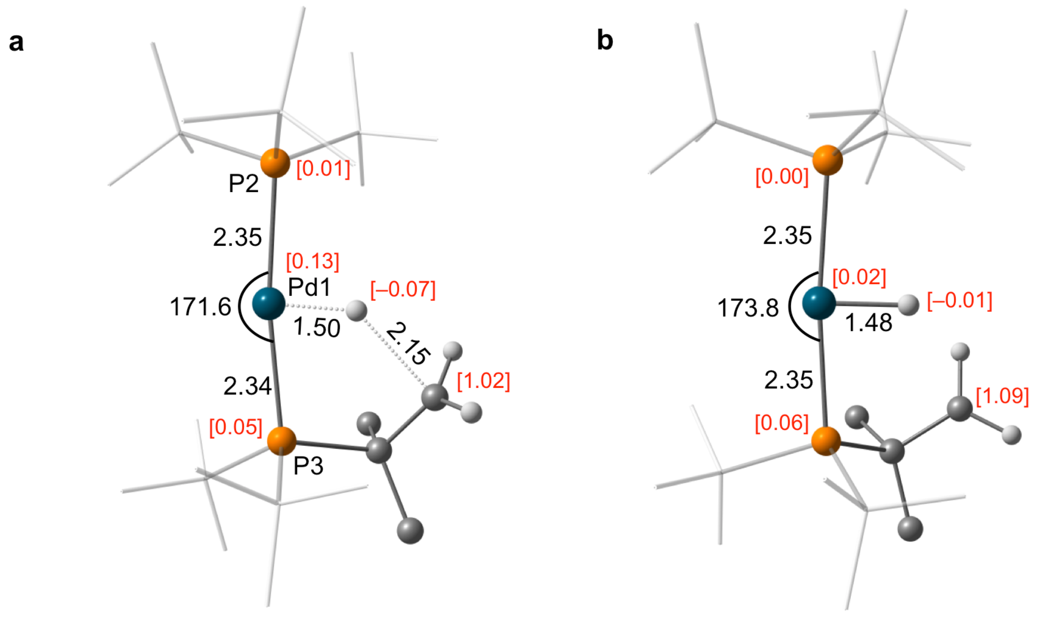

Figure S79. Optimised geometries of (a) precursor complex **TS**$_{\mathbf{HAT}}$ (S = ½) and (b) **3**$_{\mathbf{HAT}}$ (S = ½) with key bond distances (Å), angles (°), and spin populations (red numbers in square brackets).

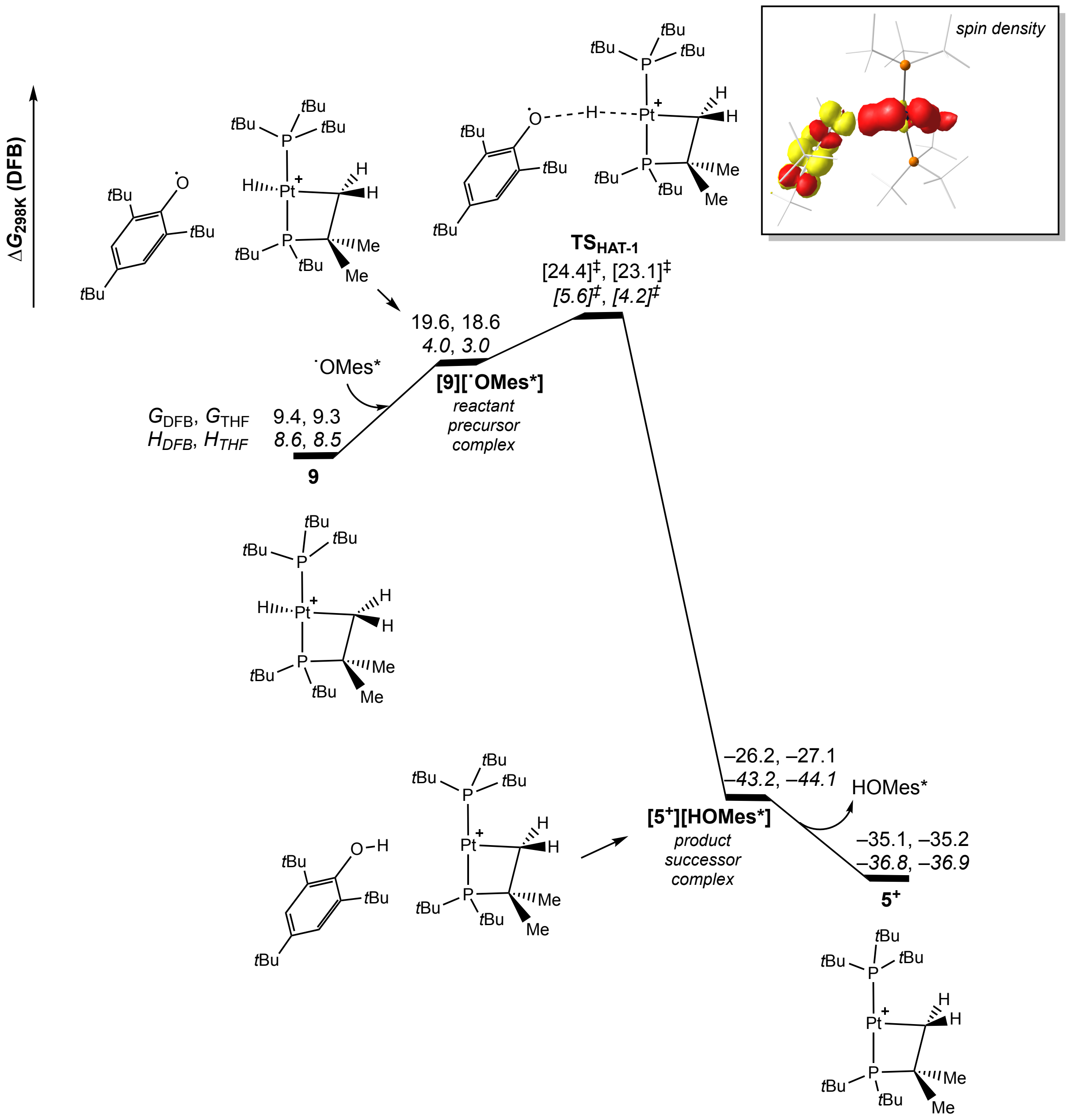

Figure S80. Computed reaction profile for the conversion of **9** into **5⁺** at the B2PLYP-D3(BJ)/def2-TZVPP+def-ECP(Pt)//PBEh-3c level of theory. Energies corrected for DFB and THF solvent. The inset depicts the spin density of TS_{HAT-1} (isosurface 0.005 au).

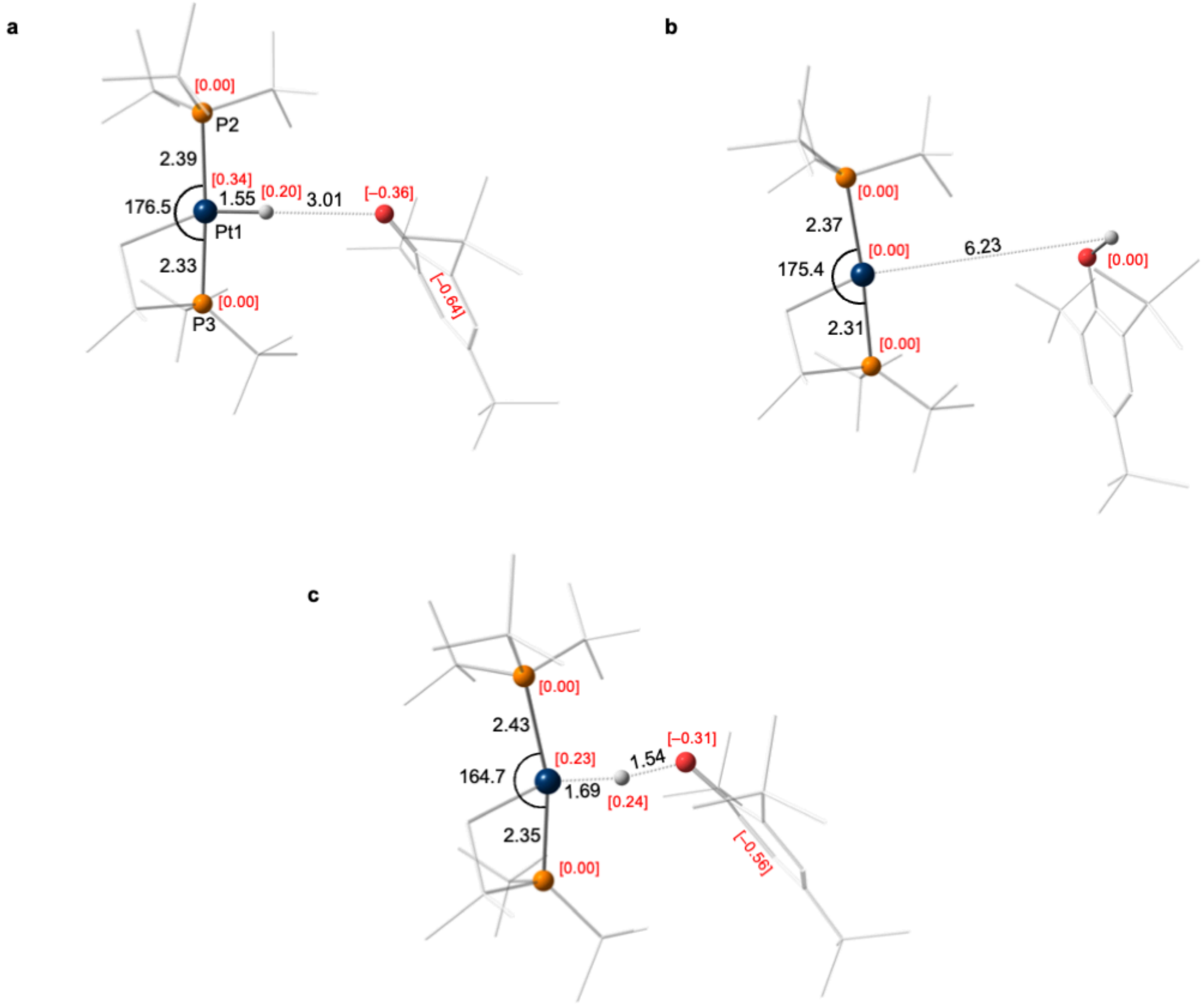

Figure S81. Optimised geometries of (a) reactant precursor complex **[9][OMes*]** (M_S = 0), (b) product successor complex **[5⁺][HOMes*]** (S = 0), (c) biradical transition state **TS$_{HAT-1}$** (M_S = 0) with key bond distances (Å), angles (°), and spin populations (red numbers in square brackets).

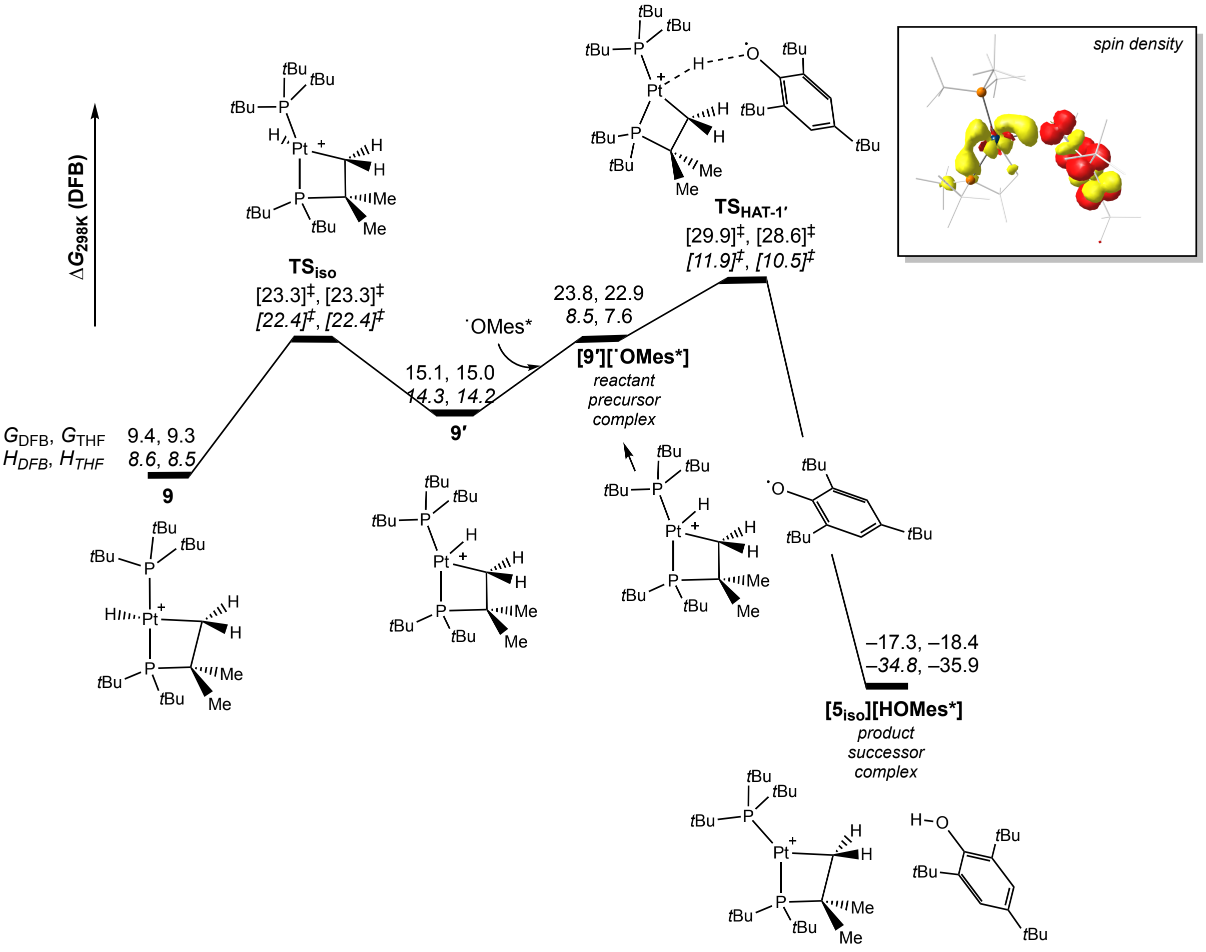

Figure S82. Computed reaction profile for the conversion of **9** into **5**$^{+}$ via isomerisation to **9′** at the B2PLYP-D3(BJ)/def2-TZVPP+def-ECP(Pt)//PBEh-3c level of theory. Energies corrected for DFB and THF solvent. The insets depict the spin density of **TS**$_{\mathbf{HAT\text{-}1'}}$ (isosurface 0.005 au).

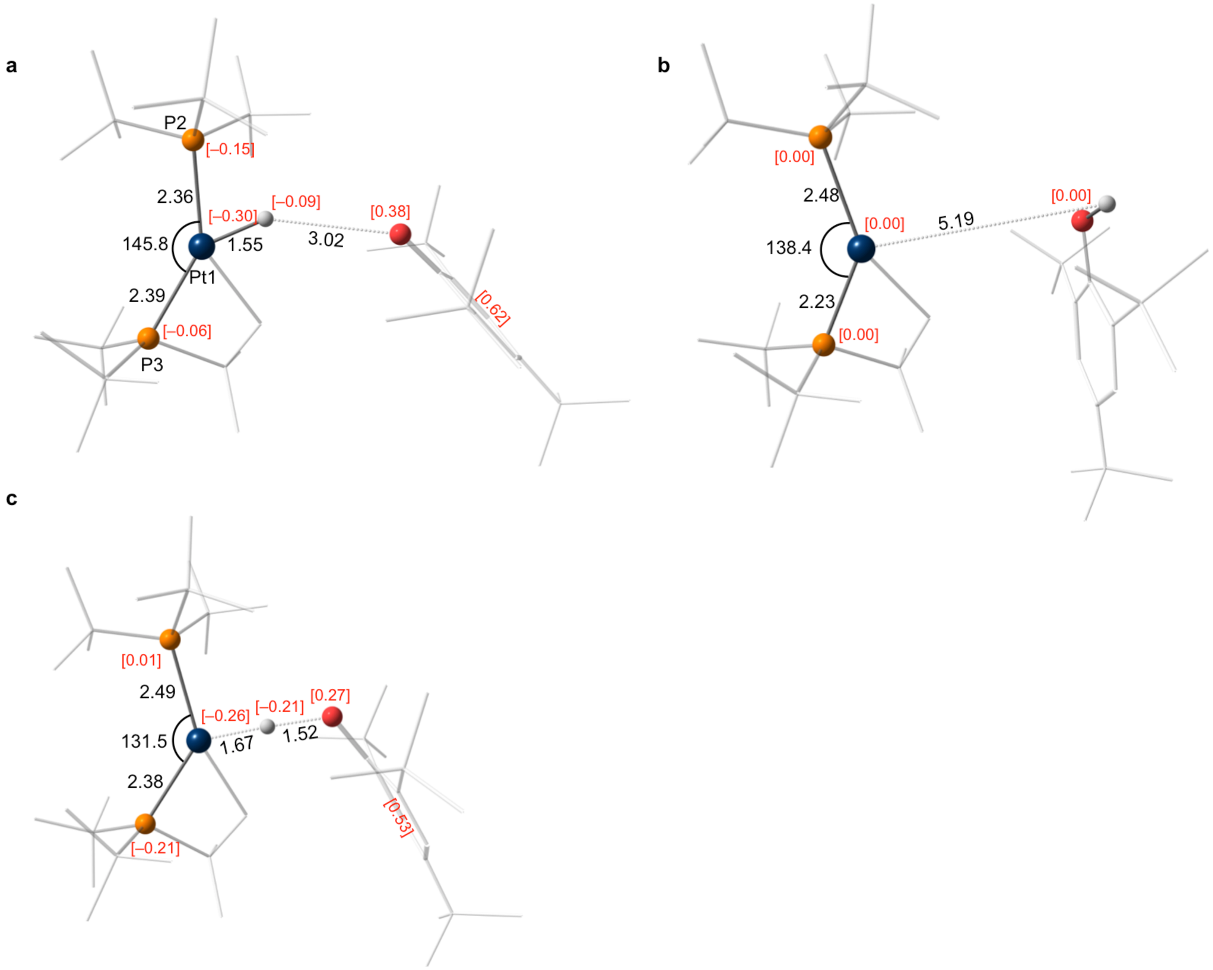

Figure S83. Optimised geometries of (a) reactant precursor complex **[9′][OMes*]** (M_S = 0), (b) product successor complex **[5$_{iso}$][HOMes*]** (S = 0), (c) biradical transition state **TS$_{HAT-1'}$** (M_S = 0) with key bond distances (Å), angles (°), and spin populations (red numbers in square brackets).

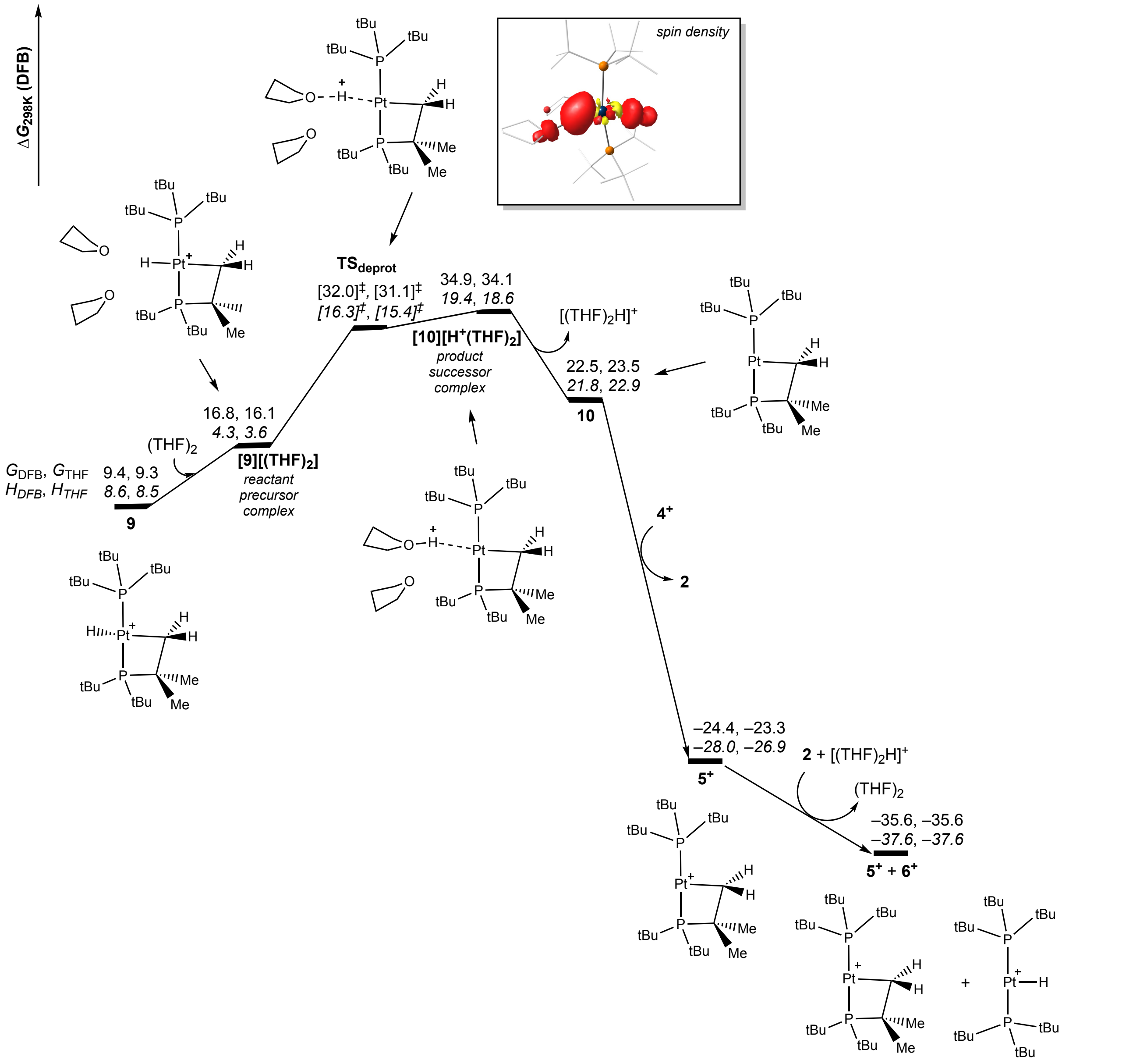

Figure S84. Computed reaction profile for the conversion of **9** into **5⁺** via proton transfer to THF at the B2PLYP-D3(BJ)/def2-TZVPP+def-ECP(Pt)//PBEh-3c level of theory. Energies corrected for DFB and THF solvent. The insets depict the spin density of **TS**$_{\mathbf{deprot}}$ (isosurface 0.005 au). The electronic energy of the **TS**$_{\mathbf{deprot}}$ product successor complex is lower than the transition state, but the calculated Free Energy is anomalously elevated due to uncancelled thermal and entropic corrections.

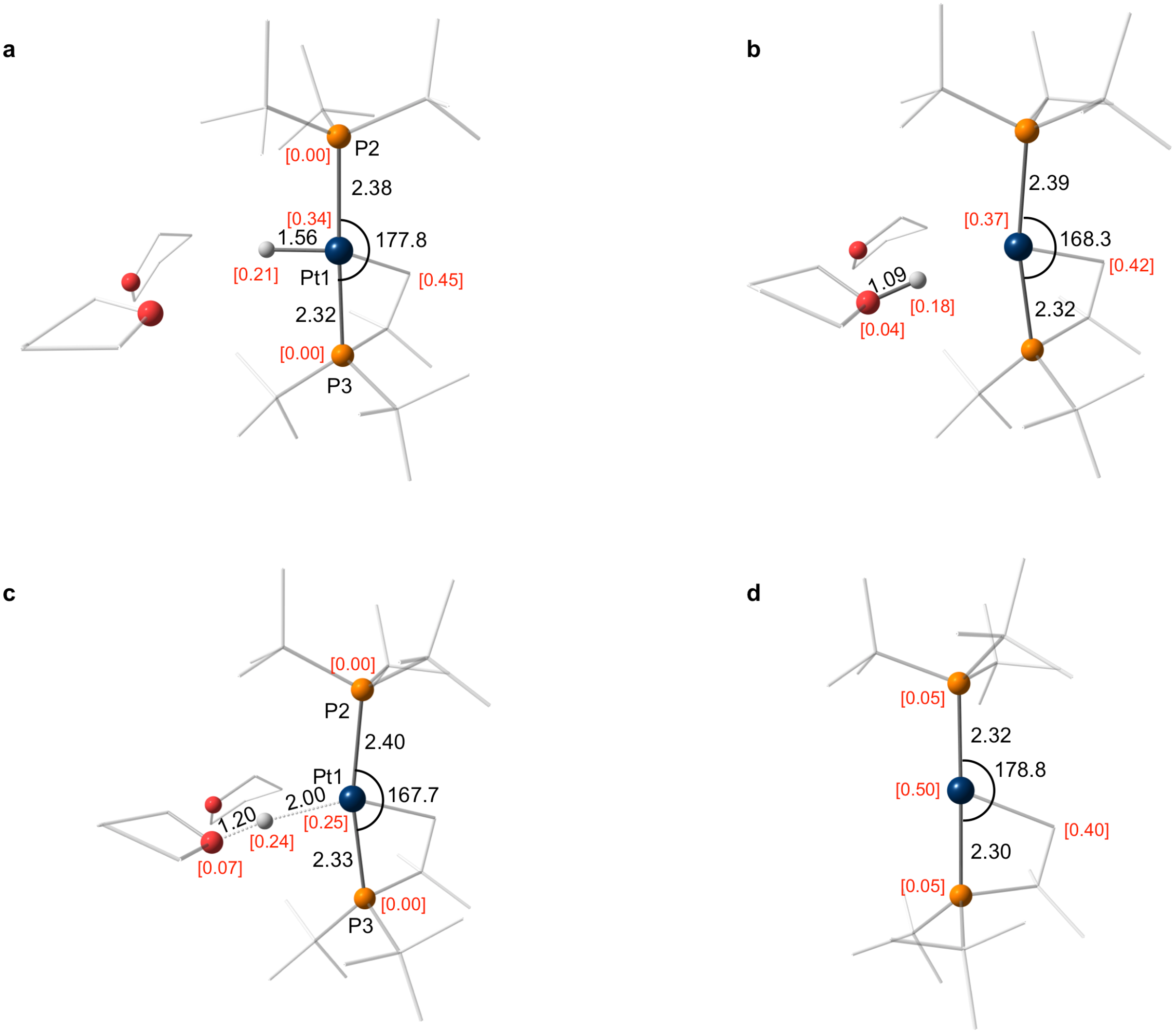

Figure S85. Optimised geometries of (a) precursor complex **[9][(THF)$_2$]** (S = ½), (b) product complex **[10][H$^+$(THF)$_2$]** (S = ½), (c) transition state **TS$_{deprot}$** (S = ½) and (d) **10** (S = ½) with key bond distances (Å), angles (°), and spin populations (red numbers in square brackets).

12.4 Structure and properties of [M(PAd$_3$)$_2$]$^+$ (M = Pd, 13; Pt, 14).

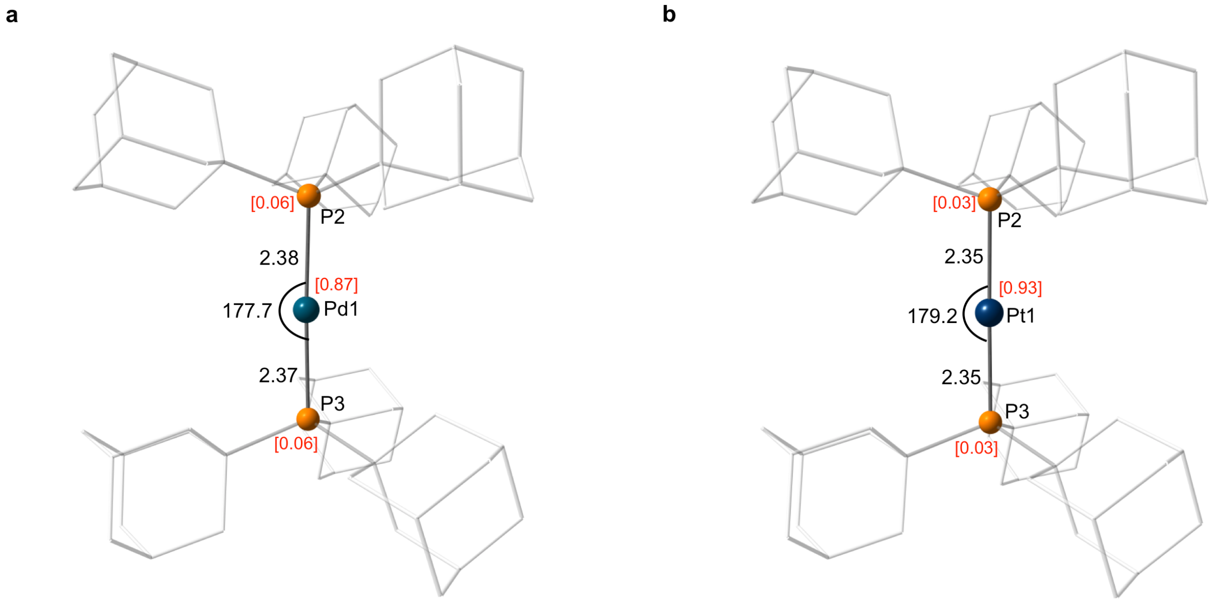

Figure S86. Optimised geometries of (a) **13** (S = ½) and (b) **14** (S = ½) at the PBEh-3c level of theory with key bond distances (Å), angles (°), and spin populations (red numbers in square brackets). Hydrogens on ligands omitted for clarity.

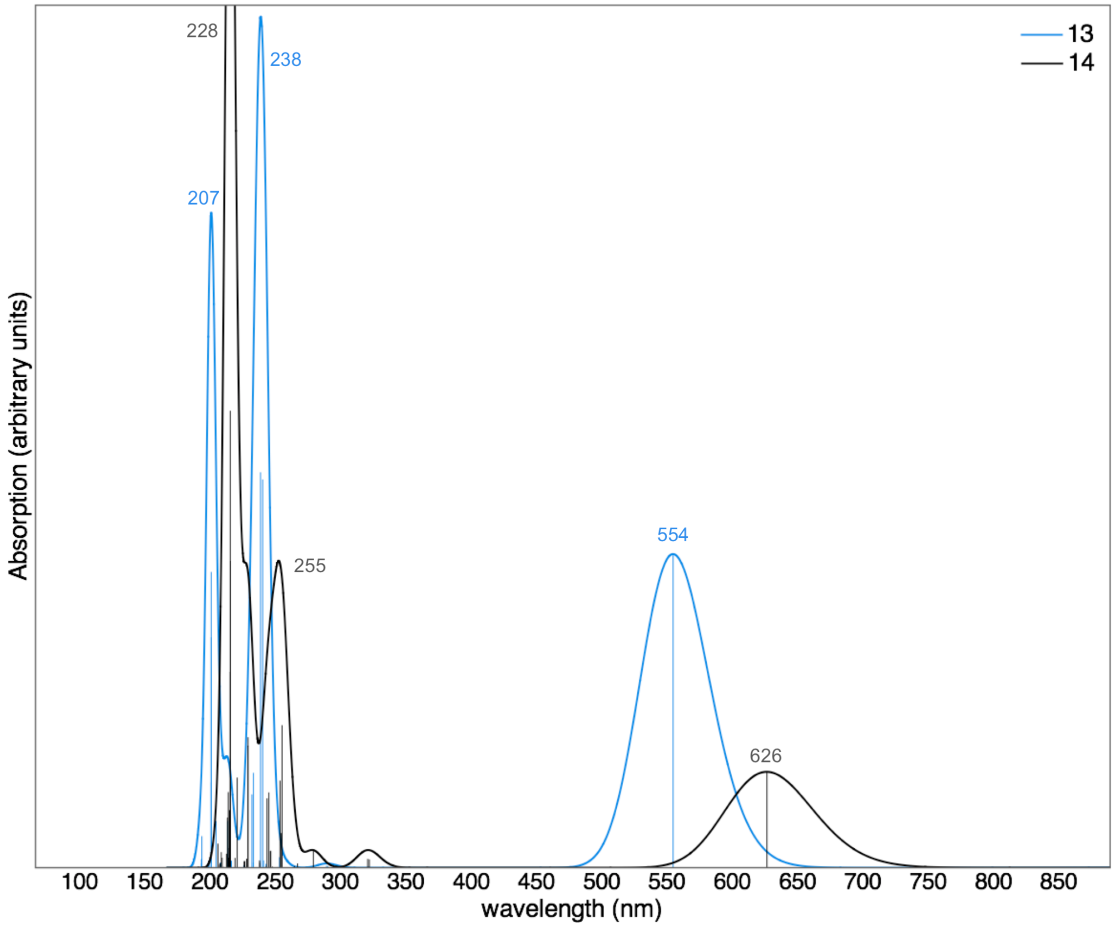

Figure S87. Calculated spectra of **13** and **14** at the CAM-B3LYP-D3(BJ)/def2-TZVP+def2-ECP(Pd/Pt) level of theory. Absorption energies include corrections due to effects from DFB solvent.

12.5 Reactions between $[M(PR_3)_2]^+$ and 9,10-dihydroantracene (9,10-AnH_2)

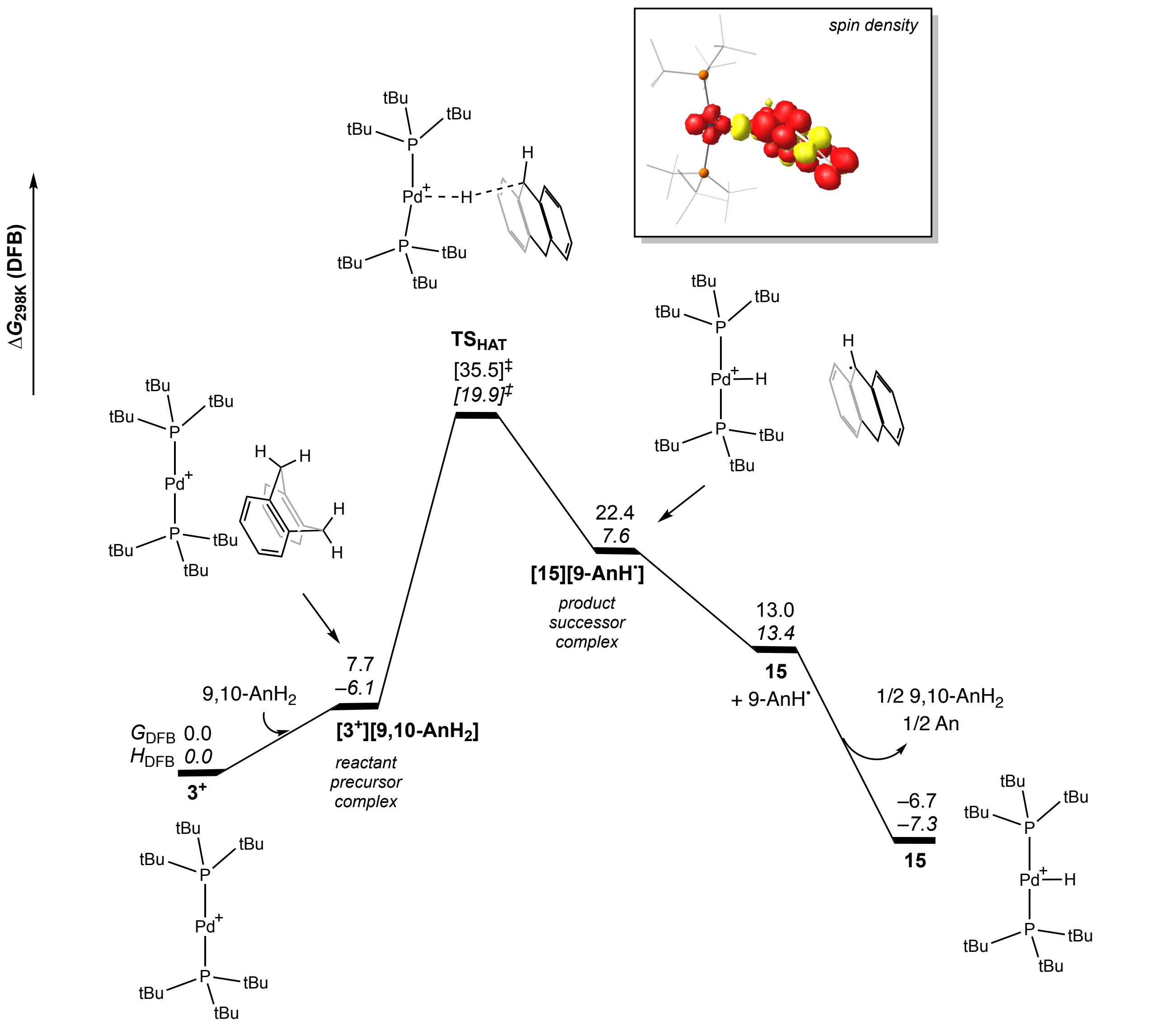

Figure S88. Computed reaction profile for carbon-to-metal H-atom transfer from 9,10-dihydroanthracene **3**$^+$ at the B2PLYP-D3(BJ)/def2-TZVPP+def-ECP(Pd)//PBEh-3c level of theory. Energies corrected for DFB solvent. The inset depicts the spin density of the transition state (isosurface 0.005 au).

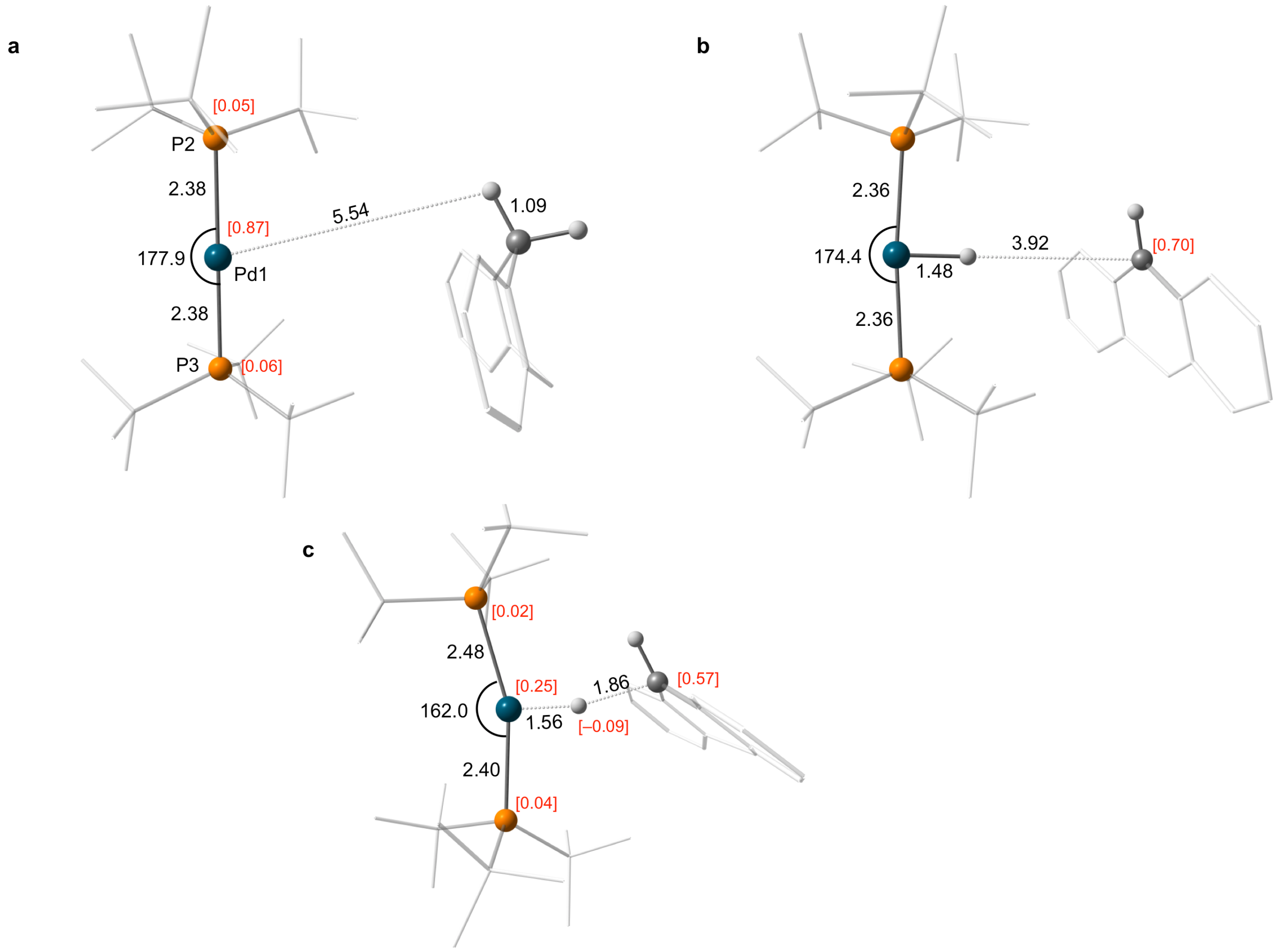

Figure S89. Optimised geometries of (a) precursor complex **[3⁺][9,10-AnH₂]** (*S* = ½), (b) product complex **[15][9-AnH]** (*S* = ½), and (c) transition state **TS$_{HAT}$** (*S* = ½) with key bond distances (Å), angles (°), and spin populations (red numbers in square brackets).

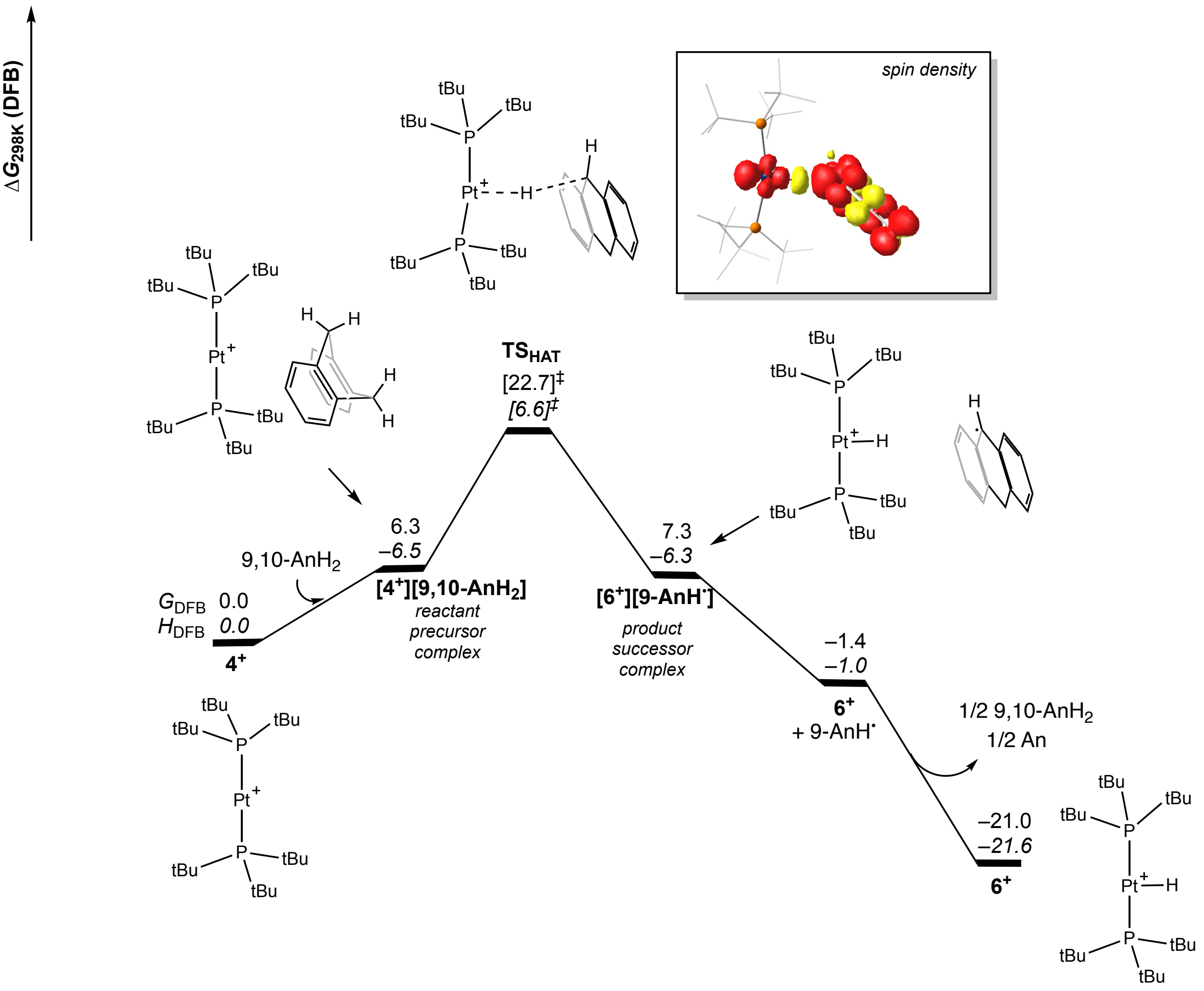

Figure S90. Computed reaction profile for carbon-to-metal H-atom transfer from 9,10-dihydroanthracene to **4⁺** at the B2PLYP-D3(BJ)/def2-TZVPP+def-ECP(Pt)//PBEh-3c level of theory. Energies corrected for DFB solvent. The inset depicts the spin density of the transition state (isosurface 0.005 au).

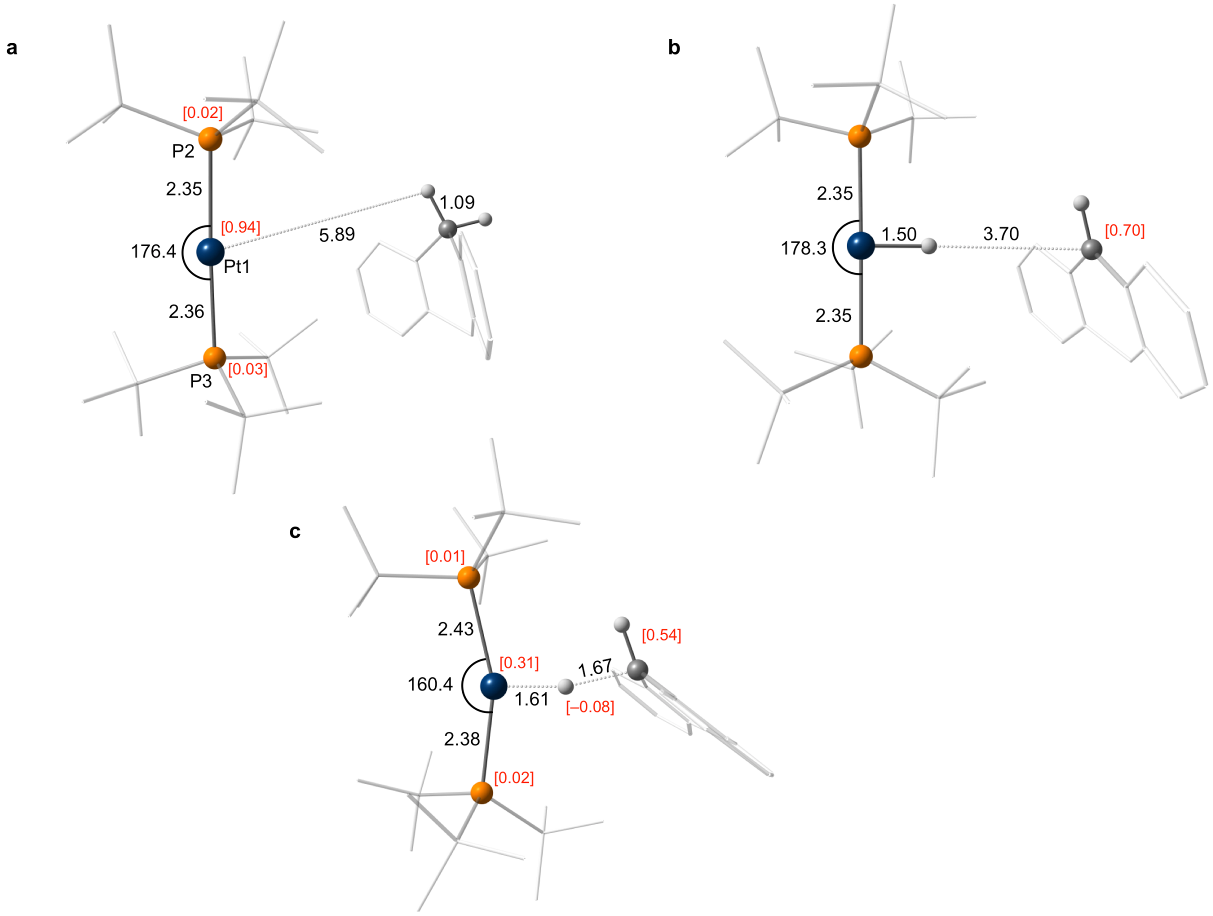

Figure S91. Optimised geometries of (a) precursor complex **[4$^+$][9,10-AnH$_2$]** (*S* = ½), (b) product complex **[6$^+$][9-AnH]** (*S* = ½), and (c) transition state **TS$_{HAT}$** (*S* = ½) with key bond distances (Å), angles (°), and spin populations (red numbers in square brackets).

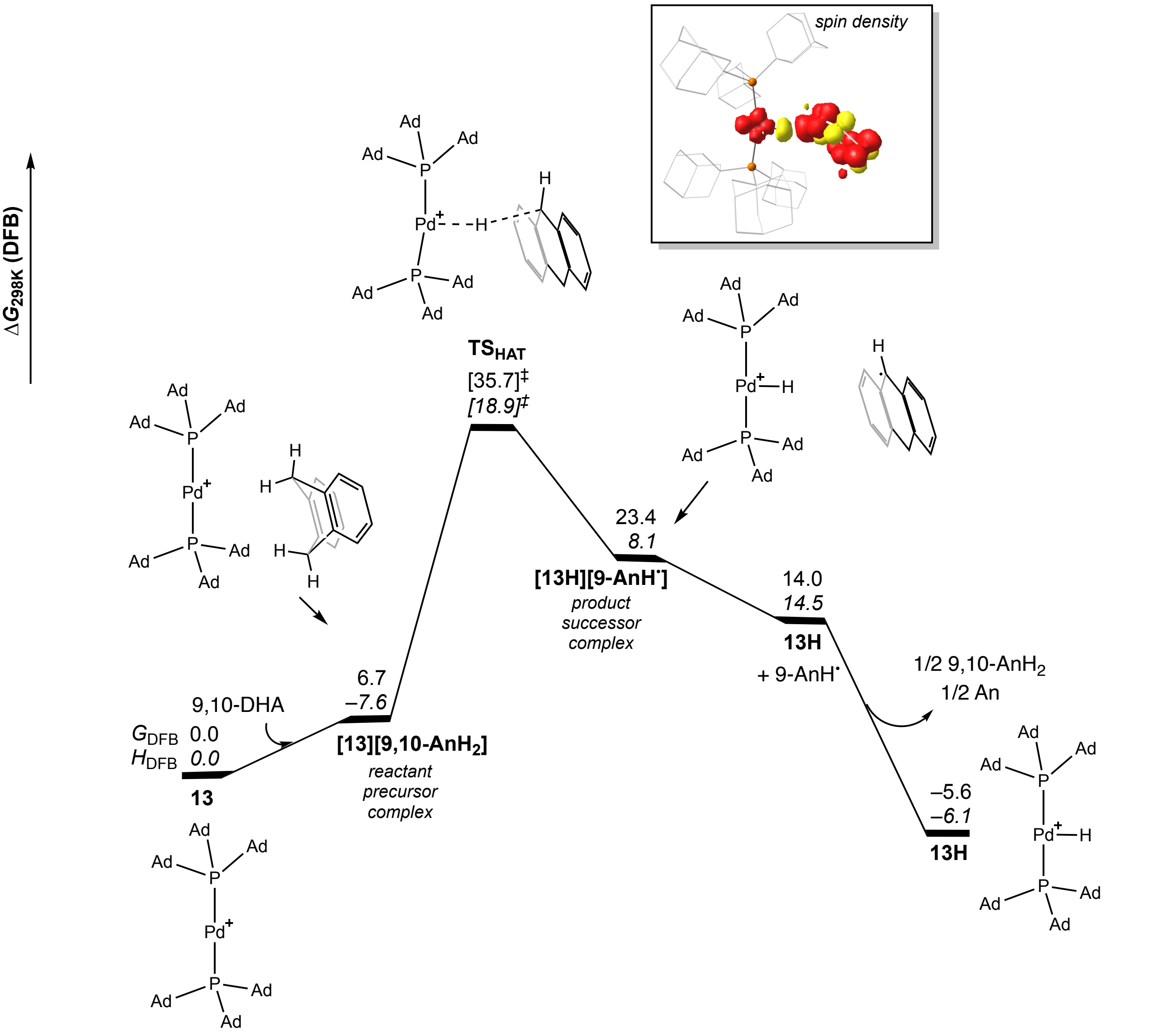

Figure S92. Computed reaction profile for carbon-to-metal H-atom transfer from 9,10-dihydroanthracene to **13** at the B2PLYP-D3(BJ)/def2-TZVPP+def-ECP(Pd)//PBEh-3c level of theory. Energies corrected for DFB solvent. The inset depicts the spin density of the transition state (isosurface 0.005 au).

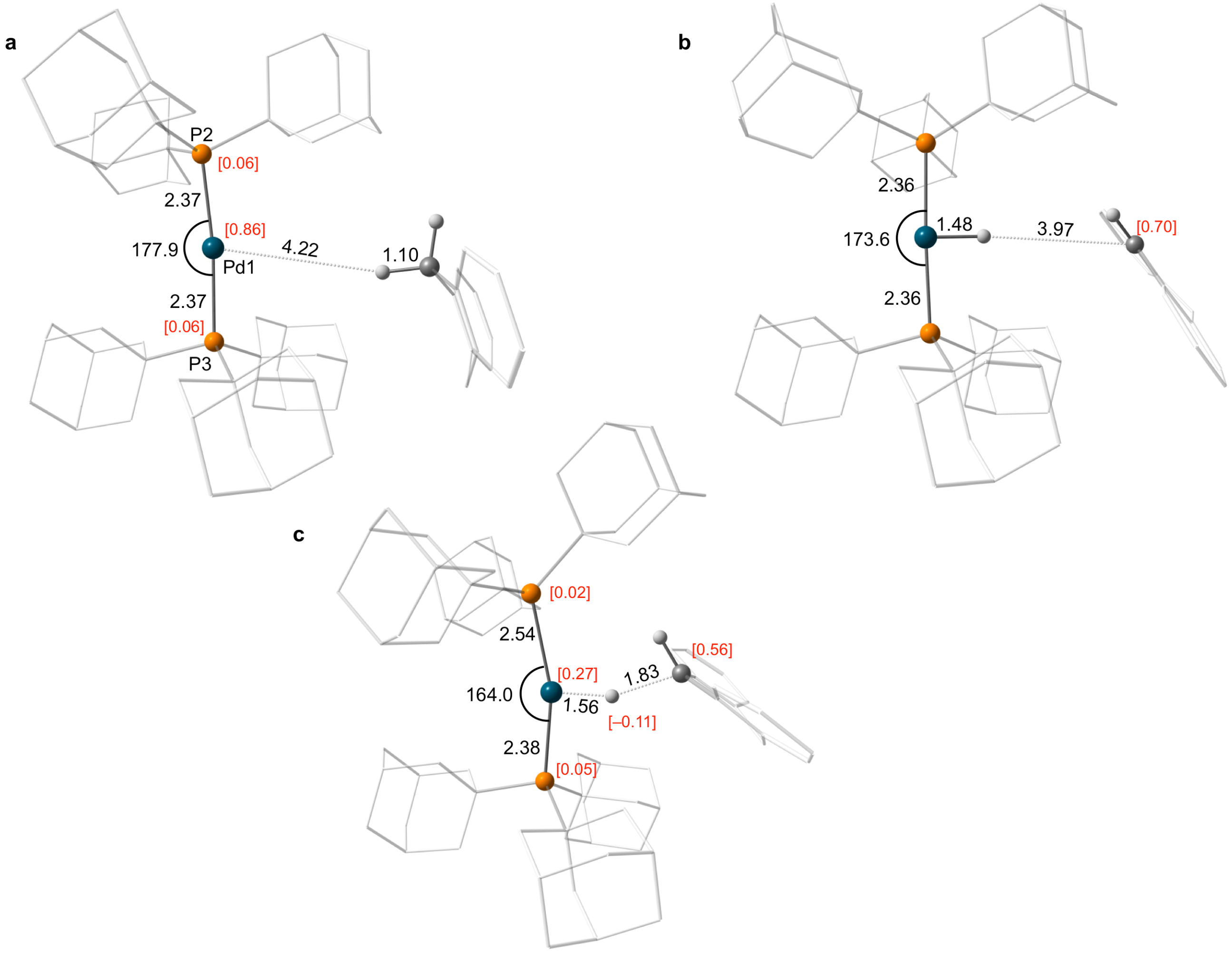

Figure S93. Optimised geometries of (a) precursor complex **[13][9,10-AnH$_2$]** (S = ½) (b) product complex **[13H][9-AnH]** (S = ½) and (c) transition state **TS$_{HAT}$** (S = ½) with key bond distances (Å), angles (°), and spin populations (red numbers in square brackets).

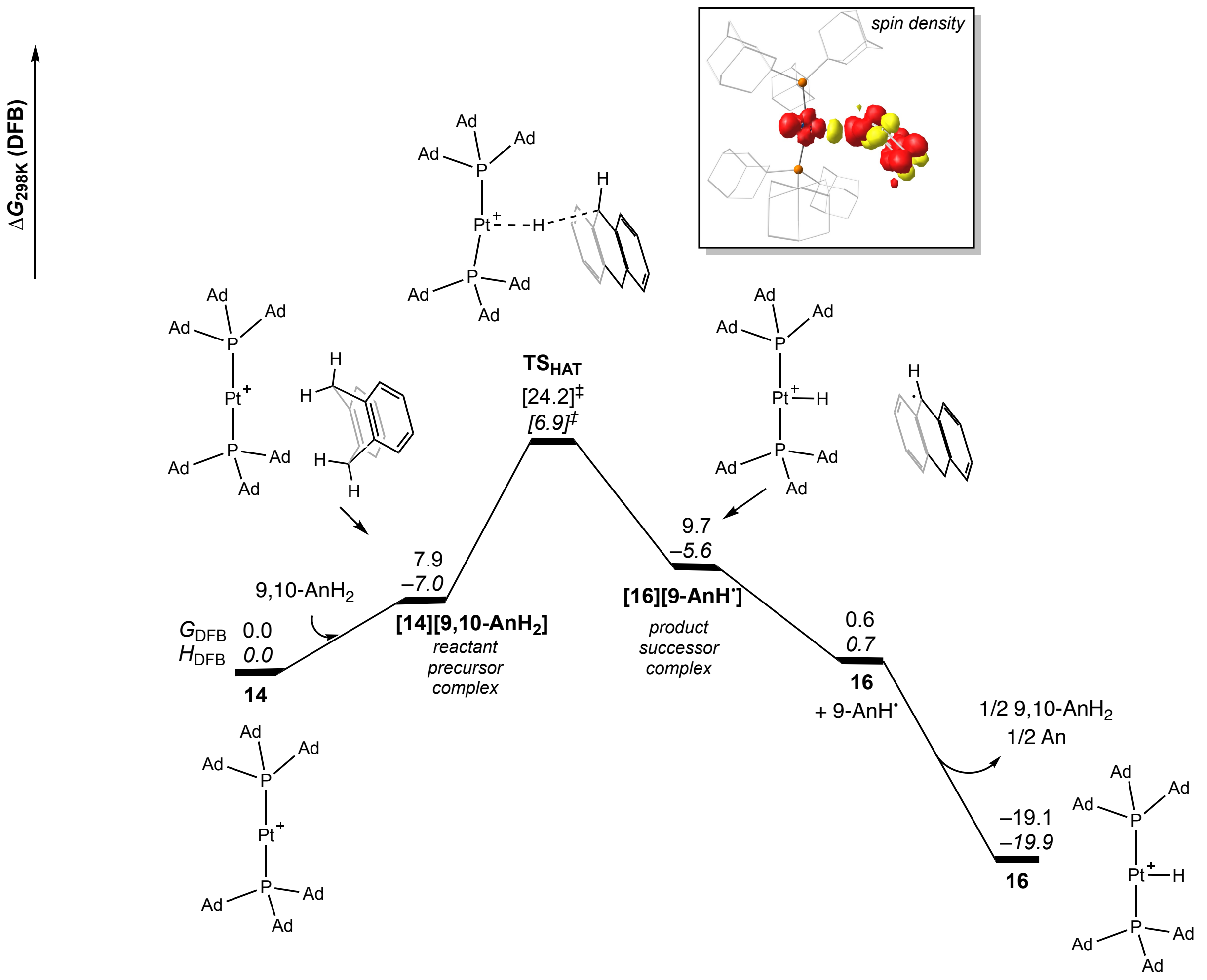

Figure S94. Computed reaction profile for carbon-to-metal H-atom transfer from 9,10-dihydroanthracene to **14** at the B2PLYP-D3(BJ)/def2-TZVPP+def-ECP(Pt)//PBEh-3c level of theory. Energies corrected for DFB solvent. The inset depicts the spin density of the transition state (isosurface 0.005 au).

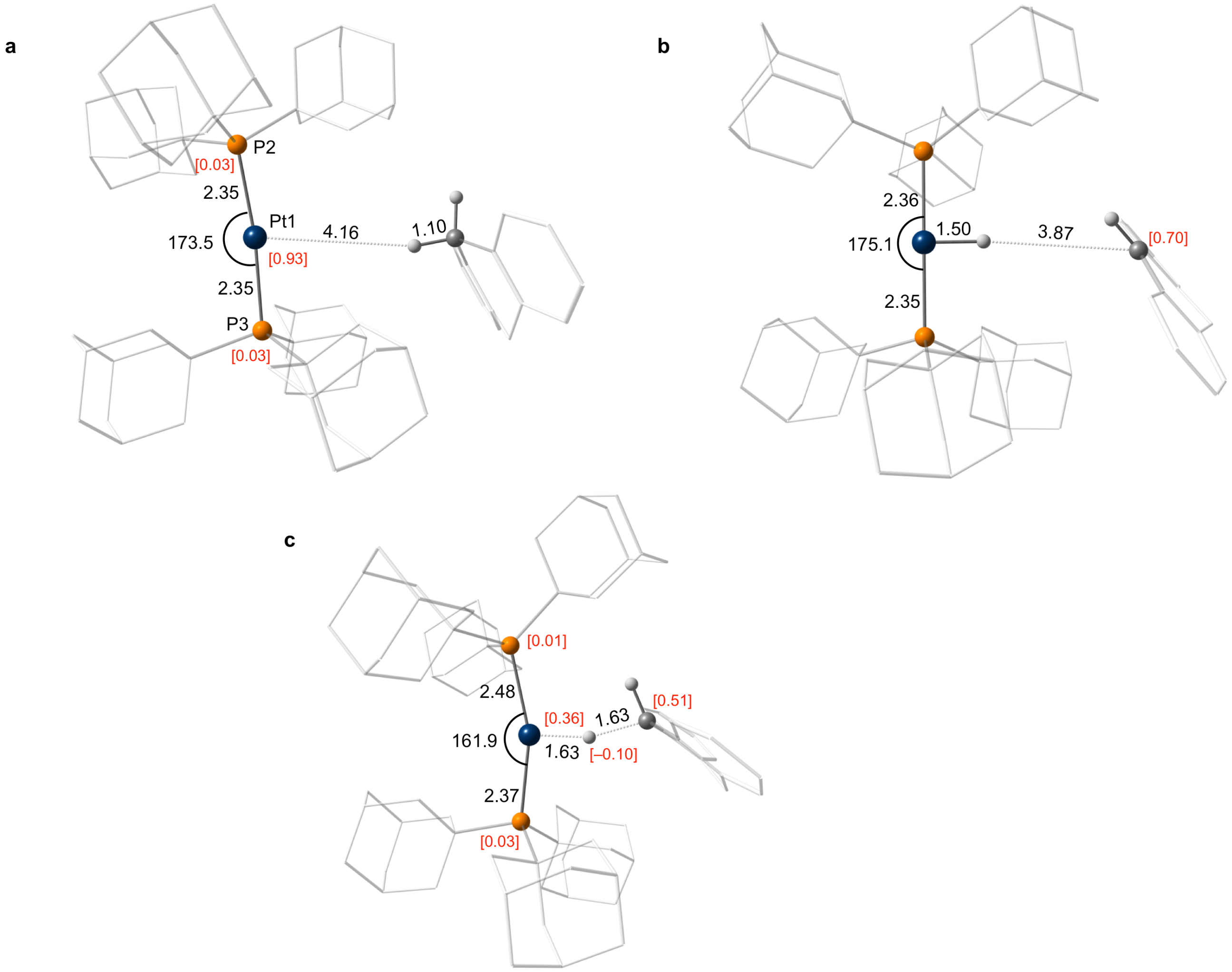

Figure S95. Optimised geometries of (a) precursor complex **[14][9,10-AnH$_2$]** (S = ½), (b) product complex **[16][9-AnH]** (S = ½), and (c) transition state **TS$_{HAT}$** (S = 0) with key bond distances (Å), angles (°), and spin populations (red numbers in square brackets).

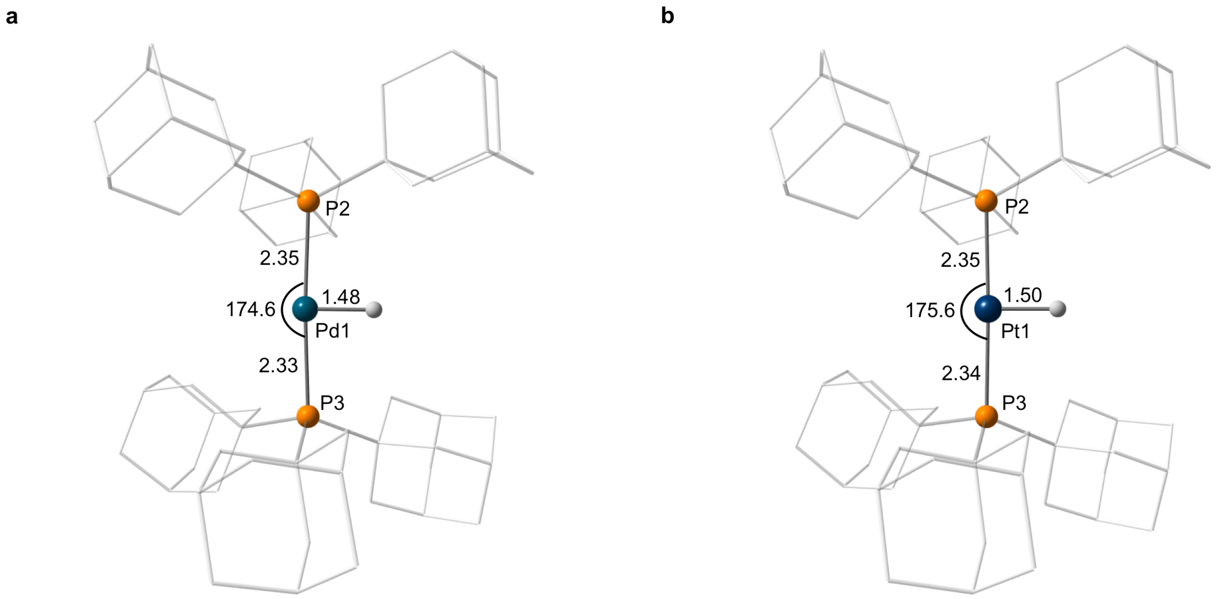

Figure S96. Optimised geometries of (a) $[Pd(PAd_3)_2H]^+$ (**13H**, *S* = 0) and (b) **16** (*S* = 0) at the PBEh-3c level of theory with key bond distances (Å) and angles (°).

12.6 Bond dissociation energies of $[M(PR_3)_2H]^+$

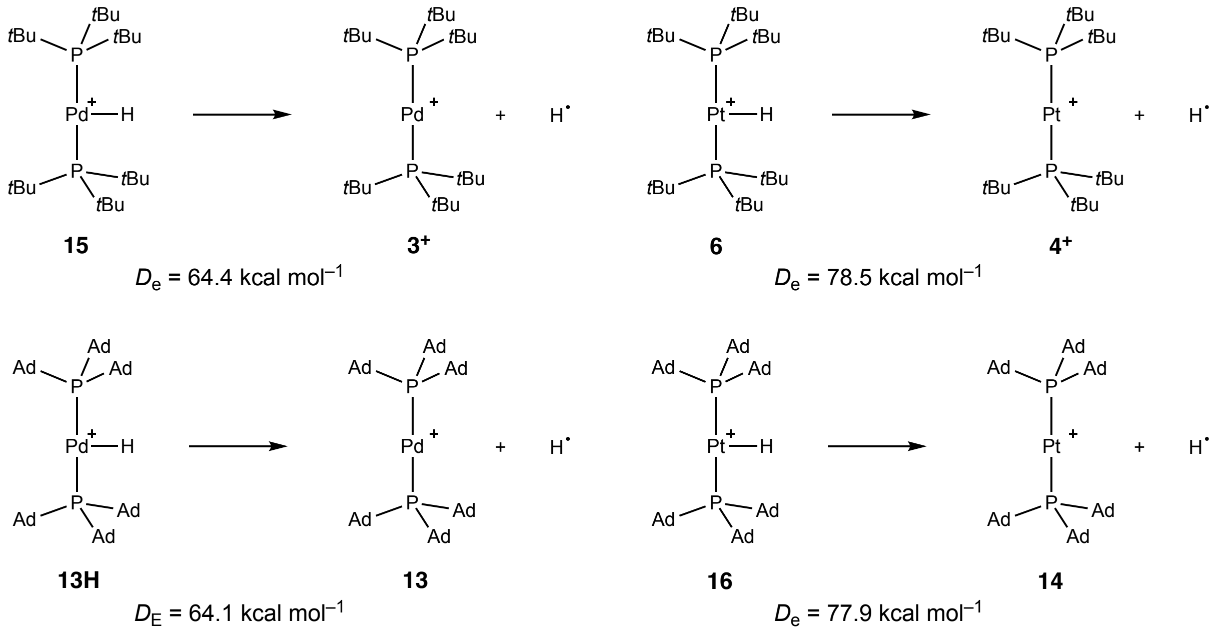

Figure S97. Gas phase M–H bond dissociation enthalpies calculated at the B2PLYP-D3(BJ)/def2-TZVPP+def-ECP(Pd/Pt)//PBEh-3c level of theory.

13 References

[1] Neese, F. Software update: The ORCA program system-Version 5.0. *WIREs Comput Mol. Sci*. **2022**, *12*, e1606.

[2] (a) Bursch, M.; Mewes, J.-M.; Hansen, A.; Grimme, S. Best-Practice DFT Protocols for Basic Molecular Computational Chemistry. *Angew. Chem. Int. Ed*., **2022**, *61*, e202205735; (b) Grimme, S. Exploration of Chemical Compound, Conformer, and Reaction Space with Meta-Dynamics Simulations Based on Tight-Binding Quantum Chemical Calculations. *J. Chem. Theory Comput*., **2019**, *15*, 2847–2862.

[3] Bannwarth, C.; Ehlert, S.; Grimme, S. GFN2-xTB–An Accurate and Broadly Parametrized Self-Consistent Tight-Binding Quantum Chemical Method with Multipole Electrostatics and Density-Dependent Dispersion Contributions. *J. Chem. Theory Comput.*, **2019**, *15*, 1652–1671.

[4] Grimme, S.; Brandenburg, J. G.; Bannwarth, C.; Hansen, A., Consistent structures and interactions by density functional theory with small atomic orbital basis sets. *J. Chem. Phys*., **2015**, *143*, 054107.

[5] Weigend, F., Accurate Coulomb-fitting basis sets for H to Rn. *Phys. Chem. Chem. Phys*., **2006**, *8*, 1057–1065.

[6] Andrae, D.; Häussermann, U.; Dolg, M.; Stoll, H.; Preuss, H. Energy-Adjusted Ab Initio Pseudopotentials for the Second and Third Row Transition Elements. *Theo. Chim. Acta*, **1990**, *77*, 123–141.

[7] Kruse, H.; Grimme, S. A geometrical correction for the inter- and intra-molecular basis set superposition error in Hartree-Fock and density functional theory calculations for large systems. *J. Chem. Phys*., **2012**, *136*,151101.

[8] (a) Grimme, S.; Ehrlich, S.; Goerigk, L. Effect of the Damping Function in Dispersion Corrected Density Functional Theory. *J. Comp. Chem*., **2011**, *32*, 1456–1465; (b) Grimme, S., Antony, J.; Ehrlich, S.; Krieg, H. A consistent and accurate ab initio parametrization of density functional dispersion correction (DFT-D) for the 94 elements H-Pu. *J. Chem. Phys*., **2010**, *132*, 154104.

[9] Bühl, M.; Reimann, C.; Pantazis, D. A.; Bredow, T.; Neese, F. Geometries of third-row transition-metal complexes from density-functional theory. *J. Chem. Theory Comp*., **2008**. *4*, 1449–1459

[10] Ishida, K.; Morokuma, K.; Komornicki, A. Intrinsic Reaction Coordinate - an Ab initio Calculation for HNC → HCN and H^{-} + CH_4 →CH_4 +H^{-}. *J. Chem. Phys.*, **1977**, *66*, 2153–2156.

[11] Grimme, S., Supramolecular Binding Thermodynamics by Dispersion-Corrected Density Functional Theory. *Chem. Eur. J*., **2012**, *18*, 9955–9964.

[12] Noodleman, L., Valence Bond Description of Anti-Ferromagnetic Coupling in Transition-Metal Dimers. J. Chem. Phys., **1981**, *74*, 5737–5743.

[13] (a) Becke, A. D., Density-Functional Thermochemistry 3. The Role of Exact Exchange. *J. Chem. Phys*., **1993**, *98*, 5648–5652; (b) Lee, C. T.; Yang, W. T.; Parr, R. G. Development of the Colle-Salvetti Correlation-Energy Formula into a Functional of the Electron-Density. *Phys. Rev. B*, **1988**, *37*, 785–789; (c) Vosko, S. H.; Wilk, L. Nusair, M. Accurate Spin-Dependent Electron Liquid Correlation Energies for Local Spin-Density Calculations - a Critical Analysis. *Can. J. Phys*., **1980**,

58, 1200–1211; (d) Stephens, P. J., Devlin, F. J.; Chabalowski, C. F.; Frisch, M. J. Ab Initio Calculation of Vibrational Absorption and Circular-Dichroism Spectra Using Density-Functional Force-Fields. *J. Phys. Chem.*, **1994**, *98*, 11623–11627.

[14] Chai, J. D.; Head-Gordon, M. Long-range corrected hybrid density functionals with damped atom-atom dispersion corrections. *Phys. Chem. Chem. Phys.*, **2008**, *10*, 6615–6620.

[15] Mardirossian, N.; Head-Gordon, M. ωB97X-V: A 10-parameter, range-separated hybrid, generalized gradient approximation density functional with nonlocal correlation, designed by a survival-of-the-fittest strategy. *Phys. Chem. Chem. Phys.*, **2014**, *16*, 9904–9924.

[16] Grimme, S. Semiempirical hybrid density functional with perturbative second-order correlation. *J. Chem. Phys.*, **2006**, *124*, 034108.

[17] (a) Riplinger, C.; Neese, F. An efficient and near linear scaling pair natural orbital based local coupled cluster method. J. Chem. Phys., **2013**, *138*, 034106; (b) Riplinger, C., Sandhoefer, B.; Hansen, A.; Neese F. Natural triple excitations in local coupled cluster calculations with pair natural orbitals. *J. Chem. Phys.*, **2013**, *139*, 134101; (c) Saitow, M.; Becker, U.; Riplinge, C.; Valeev, E. F., Neese, F. M. A new near-linear scaling, efficient and accurate, open-shell domain-based local pair natural orbital coupled cluster singles and doubles theory. *J. Chem. Phys.*, **2017**, *146*, 164105; (d) Guo, Y. Riplinger, C.; Becker, U.; Liakos, D. G., Minenkov, Y.; Cavallo, L.; Neese, F. Communication: An improved linear scaling perturbative triples correction for the domain based local pair-natural orbital based singles and doubles coupled cluster method [DLPNO-CCSD(T)]. *J. Chem. Phys.*, **2018**, *148*, 011101; (e) 29. Pinksi, P; Riplinger, C.; Valeev, E. F.; Neese, F. Sparse maps-A systematic infrastructure for reduced-scaling electronic structure methods. II. Linear scaling domain based pair natural orbital coupled cluster theory. *J. Chem. Phys.*, **2016**, *144*, 034108.

[18] Weigend, F.; Ahlrichs, R. Balanced basis sets of split valence, triple zeta valence and quadruple zeta valence quality for H to Rn: Design and assessment of accuracy. *Phys. Chem. Chem. Phys*, **2005**, *7*, 3297–3305.

[19] Hellweg, A.; Hättig, C.; Höfener, S.; Klopper, W. Optimized accurate auxiliary basis sets for RI-MP2 and RI-CC2 calculations for the atoms Rb to Rn. *Theor. Chem. Acc.*, **2007**, *117*, 587–597.

[20] Neese, F.; Wennmohs, F.; Hansen, A.; Becker, U. Efficient, approximate and parallel Hartree-Fock and hybrid DFT calculations. A 'chain-of-spheres' algorithm for the Hartree-Fock exchange. *Chem. Phys.*, **2009**, *356*, 98–109.

[21] Marenich, A. V.; Cramer, C. J.; Truhlar, D. G. Universal Solvation Model Based on Solute Electron Density and on a Continuum Model of the Solvent Defined by the Bulk Dielectric Constant and Atomic Surface Tensions. *J. Phys. Chem. B*, **2009**, *113*, 6378–6396.

[22] (a) Keith, J. A.; Carter, E. A. Quantum Chemical Benchmarking, Validation, and Prediction of Acidity Constants for Substituted Pyridinium Ions and Pyridinyl Radicals. *J. Chem. Theory Comput.*, **2012**, *8*, 3187–3206; (b) Tomanik, L.; Muchová, E.; Slaviček, P. Solvation energies of ions with ensamble cluster-continuum approach. *Phys. Chem. Chem. Phys.*, **2020**, *22*, 22357–22368.

[23] Yanai, T.; Tew, D. P.; Handy, N. C. A new hybrid exchange-correlation functional using the Coulomb-attenuating method (CAM-B3LYP). *Chem. Phys. Lett.*, **2004**, *393*, 51–57.

[24] Cammi, R.; Mennucci, B.; Tomasi, J. Fast evaluation of geometries and properties of excited molecules in solution: A Tamm-Dancoff model with application to 4-dimethylaminobenzonitrile. *J. Phys. Chem. A*, **2000**, *104*, 5631–5637.

[25] Zhurko, G. A. *Chemcraft – graphical software for visualization of quantum chemistry computations*, version 1.8. https://www.chemcraftprog.com (accessed 2023-05-16).
